# Trim21-mediated CCT2 ubiquitination suppresses malignant progression and promotes CD4^+^T cell activation in breast cancer

**DOI:** 10.1038/s41419-024-06944-8

**Published:** 2024-07-30

**Authors:** Xi Chen, Chenao Ma, Yaming Li, Yiran Liang, Tong Chen, Dianwen Han, Dan Luo, Ning Zhang, Wenjing Zhao, Lijuan Wang, Bing Chen, Hong Guo, Qifeng Yang

**Affiliations:** 1 Department of Breast Surgery, General Surgery, Qilu Hospital, Cheeloo College of Medicine, Shandong University, Jinan, Shandong China; 2 Biological Resource Center, Qilu Hospital of Shandong University, Jinan, Shandong China; 3 Shandong Desheng Bioengineering Company Limited, Jinan, Shandong China; 4 Research Institute of Breast Cancer, Shandong University, Jinan, Shandong China

**Keywords:** Breast cancer, Cancer microenvironment

## Abstract

Breast cancer remains a significant global health challenge, and its mechanisms of progression and metastasis are still not fully understood. In this study, analysis of TCGA and GEO datasets revealed a significant increase in CCT2 expression in breast cancer tissues, which was associated with poor prognosis in breast cancer patients. Functional analysis revealed that CCT2 promoted breast cancer growth and metastasis through activation of the JAK2/STAT3 signaling pathway. Additionally, the E3 ubiquitin ligase Trim21 facilitated CCT2 ubiquitination and degradation, significantly reversing the protumor effects of CCT2. Most interestingly, we discovered that exosomal CCT2 derived from breast cancer cells suppressed the activation and proinflammatory cytokine secretion of CD4^+^ T cell. Mechanistically, exosomal CCT2 constrained Ca^2+^-NFAT1 signaling, thereby reducing CD40L expression on CD4^+^ T cell. These findings highlight CCT2 upregulation as a potential driver of breast cancer progression and immune evasion. Our study provides new insights into the molecular mechanisms underlying breast cancer progression, suggesting that CCT2 is a promising therapeutic target and prognostic predictor for breast cancer.

## Introduction

Breast cancer has emerged as the most prevalent cancer and the second leading cause of cancer-related death among females globally [1]. Despite significant advancements in early detection and treatment, the persistent challenges of recurrence and metastasis continue to threaten patient survival [2]. Moreover, the clinical landscape is marked by substantial heterogeneity in metastatic potential, attributable to multifaceted genetic, epigenetic, and tumor microenvironmental factors [3]. Given the incomplete understanding of the molecular mechanisms of breast cancer, further research needed to identify novel pathogenic determinants and therapeutic targets.

Chaperonin-containing TCP1 subunit 2 (CCT2), a constituent of the CCT chaperonin complex alongside eight other subunits, is typically involved in facilitating the proper folding of nascent polypeptides [4]. Recent studies have implicated CCT2 in serving as an autophagy receptor, regulating the clearance of aggregation-prone proteins independently of ubiquitin-binding receptors or chaperone-mediated autophagy [5]. Furthermore, human umbilical cord-derived mesenchymal stem cells have been shown to mitigate liver ischemia/reperfusion injury through modulating CCT2 expression in extracellular vesicles [6]. While investigations into the expression and role of CCT2 in human malignancies are somewhat limited, upregulated CCT2 expression has been documented in various cancers, including neuroblastoma, colon cancer, and small cell lung cancer, and is correlated with an unfavorable prognosis [7–9]. Additionally, CCT2 has been implicated in regulating the folding of Gli-1 under hypoxic conditions in colorectal cancer [10]. However, its involvement in breast cancer progression and the underlying mechanisms remain unexplored.

Recent research has emphasized the crucial interplay between cancer cells and the tumor microenvironment, particularly involving stromal cells, in driving tumor progression [11]. In breast cancer, the stromal compartment comprises fibroblasts, endothelial cells, and tumor-infiltrating inflammatory cells [12]. Among these, CD4^+^ T cells, a type of inflammatory cell, are crucial in supporting and sustaining antitumor immune responses, which are predominantly mediated by the release of cytokines into the tumor microenvironment [13]. Activated CD4^+^ T cells exhibit high expression levels of CD40L, which in turn enhances the antitumor immune response [13]. While the baseline expression of CD40L in CD4^+^ T cells is typically low under normal circumstances, its expression is upregulated upon CD4^+^ T cells activation. However, the underlying mechanism by which breast cancer cells stimulate CD4^+^ T cells activation has not been extensively elucidated.

Exosomes, crucial mediators of intercellular communication, contain a large variety of biological components in small vesicular bodies (diameter: 30–200 nm) [14]. Cancer cell-derived exosomes have garnered attention for their involvement in cancer-stroma crosstalk, facilitating the transfer of oncogenic molecules and contributing to oncogenic phenotypes, immune evasion, and chemoresistance [15]. In this study, we investigated the potential of tumor-derived exosomes to modulate CD4^+^ T cells function, potentially compromising antitumor immunity.

Our findings revealed a significant association between high CCT2 expression and adverse outcomes in breast cancer patients. Moreover, functional assays uncovered the crucial role of CCT2 in breast cancer progression via the JAK2/STAT3 signaling pathway. We elucidated a regulatory mechanism whereby Trim21-mediated ubiquitination governs CCT2 levels. Furthermore, we discovered that CCT2 can be encapsulated within exosomes and transferred from breast cancer cells to CD4^+^ T cells, where it suppresses CD4^+^ T cell activation by downregulating CD40L expression, thereby promoting tumor immune evasion and progression.

## Materials and methods

### Patient samples and cell lines

The study included 93 individuals with breast cancer diagnoses who underwent surgery at Qilu Hospital of Shandong University between 2008 and 2016. The amount of time from the operation date to the last follow-up or death date was referred to as the survival time. The study was approved by the Ethical Review Committees of Qilu Hospital of Shandong University, and all the subjects provided informed consent.

Human breast cancer cell lines (MDA-MB-231 and MDA-MB-468), a mouse breast cancer cell line (4T1), and HEK293T cells were purchased from American Type Culture Collection (Manassas, VA, USA) and grown in Dulbecco’s modified Eagle’s medium (DMEM) supplemented with 10% fetal bovine serum (Hyclone). Short tandem repeat (STR) profiling was used to authenticate the cell lines, and it was determined that there was no mycoplasma contamination.

### Drugs, siRNAs and plasmids

MCE (NJ, USA) provided the proteasome inhibitor MG132, cycloheximide (CHX), chloroquine, and a JAK2/STAT3 inhibitor (WP1066). The cell stimulation cocktail (500X) was purchased from eBioscience (USA). Gemepharma (Shanghai, China) manufactured the siRNAs and shRNAs that targeted the indicated genes. The expression vectors containing the CCT2, Trim21, HA-tagged Wild-type (wt) ubiqution (Ub), HA-tagged K48 Ub and HA-tagged K63 Ub plasmids were purchased from Origene (USA).

### Cell proliferation assay

With the MTT assay, tests for cell proliferation were conducted. Briefly, 2 × 10^3^ transfected cells were plated into 96-well plates. The cells were treated with 20ul MTT for 4–6 h at predetermined intervals. After the culture supernatant was aspirated, 100 ul of DMSO was added. A microplate reader was used to measure the absorbance at 490 nm.

### EdU assay

In accordance with the manufacturer’s instructions, an EdU incorporation assay kit (RiboBio, Guangzhou, China) was used to measure the nucleic acid content and proliferation of the cells. Briefly, a 96-well plate was seeded with 2 × 10^4^ transfected cells and left overnight. After 2.5 h of incubation with 50 μM EdU, the cells were fixed for 30 min with 4% paraformaldehyde (PFA) and stained with Apollo Dye Solution. After nucleic acid was labeled with Hoechst, images were taken via a fluorescence microscope.

### Colony formation assay

A total of 1000 transfected cells were planted into 6 cm plates and grown for more than 2 weeks to perform colony formation assays. Following washing with PBS, the cells were fixed for 15 min with methanol and subsequently stained for 20 min with 0.2% crystal violet. Colonies were then counted and photographed.

### Cell cycle assays

In total 2 × 10^5^ transfected cells were plated into 6 cm plates and grown for 48 h to analyze the cell cycle. Following cell harvesting, propidium iodide (PI) staining was applied for 30 min in the dark, and flow cytometry was used to examine the samples.

### Apoptosis assay

Using the Annexin V-fluorescein isothiocyanate Apoptosis Detection Kit I (BD, USA), apoptosis experiments were carried out post-transfection and evaluated via flow cytometry.

### Transwell assays

In total 200 ul of serum-free DMEM was used to seed 1 × 10^5^ breast cancer cells per well into transwell chambers. Subsequently, 700ul of DMEM containing 20% FBS was added to the lower compartment of the transwell chamber. The cells were allowed to migrate for 24 h or invade through Matrigel for 48 h. After 15 min of methanol fixation and 20 min of 0.2% crystal violet staining, the migrated cells were then photographed and counted.

### RNA extraction and qPCR

With the use of TRIzol reagent (Invitrogen, USA), RNA was extracted from cells or frozen tissues. A PrimeScript Reverse Transcriptase (RT) Reagent Kit (Takara, Kyoto, Japan) was used to generate complementary DNA (cDNA). TB Green^TM^ Advantage ® qPCR Premix (Takara, Kyoto, Japan) and a LightCycler 480 Detection System (Roche, Germany) were used to conduct real-time quantitative-PCR (qPCR). The 2^-△△^Ct method was utilized to evaluate the relative expression of the listed genes, with β-actin serving as the normalization control. Supplementary Table S1 lists the primer sequences and displays the means ± SDs of three independent experiments.

### Immunofluorescence staining

For 24 h, 5 × 10^5^ breast cancer cells were seeded into 24-well culture plates. After being rinsed with PBS, the cells were fixed for 30 min with 4% PFA, permeabilized for 10 min with 0.5% Triton X-100, then blocked for 1 h at room temperature with 10% goat serum in PBS. Next, the cells were incubated overnight at 4 °C with certain primary antibodies. The nuclei were stained with DAPI for 15 min at room temperature following a 2 h dark incubation period with secondary antibodies. The stained cells were observed and photographed via a fluorescence microscope. Supplementary Table S2 lists the antibodies that were used.

### Western blot analysis

The protocol for Western blot assays was followed. Briefly, protease inhibitor (PMSF) and phosphatase inhibitor (NaF)-supplemented RIPA buffer (Beyotime, Shanghai, China) was used to extract total protein from the cells. After being separated on SDS-PAGE gels, the protein samples were transferred to PVDF membranes, blocked for 1 h at room temperature with 5% skim milk in TBST, and then incubated with primary antibodies overnight at 4 °C. The target proteins were visualized via an enhanced chemiluminescence (ECL) kit (Affinity, Jiangsu, China) after being incubated with secondary antibodies for 1 h at room temperature. Supplementary Table S2 lists the antibodies that were employed.

### Coimmunoprecipitation (co-IP)

IgG and the designated antibodies were used for Co-IP in accordance with the manufacturer’s instructions. Briefly, the cell lysates were preincubated with antibodies on a rotator for 1 h at 4 °C. Then, protein A/G plus agarose (Santa, USA) was added to the samples, which was incubated overnight at 4 °C. The complexes were released by boiling for 5 min in 2×SDS-PAGE loading buffer, followed by five washes in immune precipitation assay lysis buffer supplemented with a protease inhibitor mixture.

### Isolation of CD4^+^ T cells

Leukocyte-enriched buffy coats were used to isolate human peripheral blood monocytes (PBMCs) using density gradient centrifugation and Ficoll-Paque Plus (Sigma, USA). Following the manufacturer’s protocol, CD4^+^ T cells were isolated from PBMCs via immunomagnetic selection using a CD4^+^ T cell isolation kit (Miltenyi Biotec, Germany). Flow cytometry verified that the purity was >95%. The use of PBMCs from healthy donors was approved by the Human Investigation Committee of Qilu Hospital, Shandong University, and informed consent was obtained from each participant.

Mouse CD4^+^ T cells were purified from spleens of BALB/c mice using a CD4^+^ T cell isolation kit (Miltenyi Biotec, Germany) and autoMACS following the manufacturer’s instructions.

### Exosome isolation and identification

By using differential ultracentrifugation, exosomes were isolated from the conditioned medium of breast cancer cells. To remove dead cells, the conditioned medium was centrifuged at 300 × g and 2000 × g for 10 min at 4 °C. The cell debris and large vesicles were then removed via centrifugation at 10,000 × g for 70 min at 4 °C. To collect the exosomes in the pellet, the supernatant was centrifuged at 100,000 × g for 70 min at 4 °C. Finally, the pellet was washed with 1×PBS to eliminate protein interference, a 1× PBS suspension was used, and a 0.22 μm filter was used. The final supernatant was filtered through a 0.22 μm filter to obtain exosome-depleted conditioned medium.

Transmission electron microscopy (TEM), a NanoSlight LM10 instrument, and nanoparticle tracking analysis (NTA) software were used to further assess the size and quality of the exosomes. To determine the expression of exosome markers for characterization, Western blot analysis was conducted.

### Fluorescent labeling and transfer of exosomes

To assess the uptake of breast cancer-derived exosomes by CD4^+^ T cells, the exosomes were labeled according to the manufacturer’s protocol with 10^-6^M PKH26 cell membrane labeling Dye(Sigma USA). To eliminate any remaining dye, labeled exosomes were centrifuged at 100,000 × g for 1 h after being washed with PBS. The labeled exosomes were incubated with CD4^+^ T cells and cocultured for 2 h, after which they were washed twice. The cells were subsequently washed twice with PBS, fixed with 4% PFA for 30 min at 4 °C, and subsequently examined under a fluorescence microscope (Olympus, Japan).

### In vivo animal study

Female BALB/c mice aged 6 weeks were obtained from GemPharmatech Co.Ltd (Nanjing, China). 1 × 10^6^ 4T1-shCCT2 cells or negative control cells were resuspended in 200ul PBS and subcutaneously implanted for subcutaneous inoculation. Every two days, the tumor size was measured with calipers, and the tumor volume was calculated via the following formula: volume = length × (width)^2^/2. At the conclusion of the experiments, the mice were sacrificed, and the tumors were weighed and photographed. Tumors from 4T1-shCCT2 or control cells on Day 14 were utilized, as explained below, for flow cytometry analysis. All animal procedures were approved by the Shandong University Animal Care and Use Committee.

### Flow cytometry analysis

To analyze the infiltration of immune cells into tumors, tumor tissues were isolated from sacrificed mice. After the tissues were finely minced, they were digested for 2 h at 37 °C with collagenase (100 μg/ml) and DNase (0.1 mg/ml). After digestion, the tissues were filtered through a mesh to obtain cell suspensions. Red blood cells were then removed from the suspensions using RBC lysis buffer. For human CD4^+^ T cells, the cells were directly collected to obtain single-cell suspensions. For surface staining, the cells were treated with matching antibodies for 30 min. A fixation/permeabilization kit (Ebioscience, USA) was used for intracellular staining. The cells were stimulated for 14-16 h at 37 °C with a cell stimulation cocktail (Ebioscience, USA), to stain them intracellularly for cytokines (IFNγ and IL-4). Using the matching antibodies mentioned in Supplementary Table S2, tumor-infiltrating immune cells were stained to detect CD4^+^ T cells (CD3^+^CD4^+^), CD8^+^ T cells (CD3^+^CD8^+^), macrophages (CD11b^+^F4/80^+^), and myeloid-derived suppressor cells (MDSCs) (CD11b^+^Gr-1^+^). A BD FACS Calibur flow cytometer (USA) was used to determine the fluorescence of the cells.

### Subcellular fraction

NE-PER Nuclear and Cytoplasmic Extraction Reagents (Thermo Scientific, USA) were used in accordance with the manufacturer’s instructions to extract nuclear and cytosolic proteins.

### Intracellular Ca^2+^ detection

The Ca^2+^ concentration in CD4^+^ T cells was measured to detect any slight changes among the groups. Following treatment with different types of exosomes, CD4^+^ T cells were washed twice and incubated for 30 min at 37 °C in Fluo4 AM staining solution (Beyotime, China). Using a fluorescence microscope (Olympus, Japan), fluorescence images of the cells were acquired and photographed. For flow cytometry analysis, differentially treated CD4^+^ T cells were loaded with 2.5 μM Fluo4 AM in Ca^2+^-free medium for 60 min at 37 °C, washed twice in Ca^2+^-free medium, and subjected to baseline measurements. At 60 s, ionomycin plus PMA was added to the cultures. T cells were harvested for another 9 min by flow cytometry.

### Immunohistochemistry (IHC)

Before use, the tumor tissues were embedded in paraffin and sectioned at a thickness of 4 μm. The sections were subjected to a series of procedures, including xylene deparaffinization, gradient alcohol dehydration, 3% hydrogen peroxide treatment to remove endogenous peroxidase, and microwave antigen retrieval. The sections were blocked with BSA and then incubated with primary antibodies overnight at 4 °C. The sections were subsequently incubated for 2 h at room temperature with horseradish peroxidase (HRP)-conjugated secondary antibodies. Hematoxylin counterstaining was performed after the antigen locations were marked with DAB solution. Images were captured via a light microscope (Olympus). Stained IHC sections were then assessed and imaged, as previously described [16].

### Statistical analysis

The experiments were repeated at least three times. GraphPad Prism 8.0 software was used to conduct the analyses. The data are shown as the mean ± standard deviation (SD). Student’s t-test or ANOVA was employed for two or multiple group comparisons. Pearson’s correlation coefficient analysis was used to assess correlations. Univariate and multivariate Cox proportional hazard regression models were utilized to identify independent predictors of patient prognosis. The Kaplan-Meier method was used to plot the survival curves, and the log-rank test was used to compare them. Statistics were deemed significant if *P* < 0.05.

## Results

### CCT2 is upregulated in human breast cancer tissues and is associated with poor prognosis in breast cancer patients

**Fig. 1:**
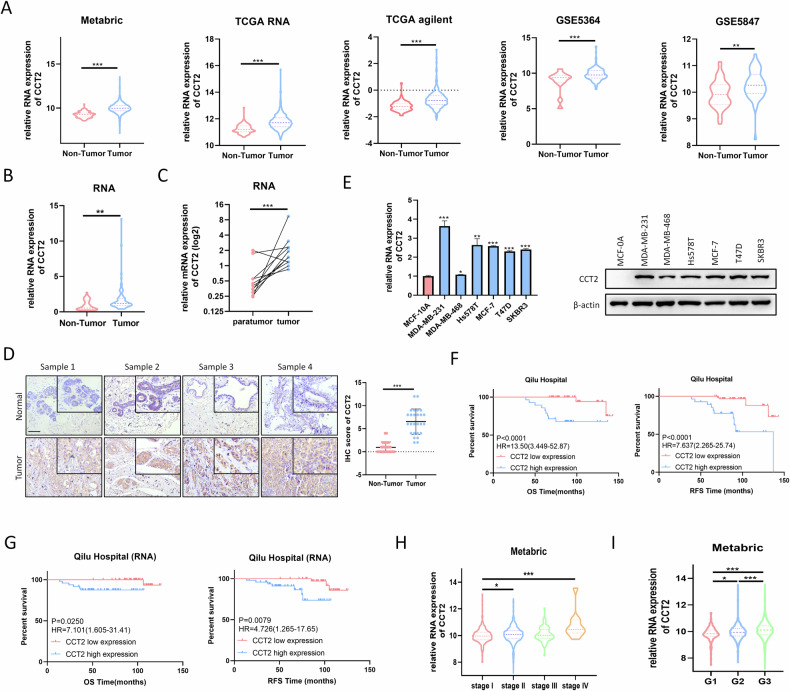
Expression of CCT2 in breast cancer tissues with its clinical significance. **A** The expression of CCT2 was elevated in breast cancer tissues compared with normal tissues according to Metabric, TCGA and GEO database. **B** the RNA expression of CCT2 was upregulated in breast cancer tissues compared with normal tissues based on Qilu cohort. **C** The RNA expression of CCT2 in breast cancer tissues and paired normal mammary tissues (*n* = 11). **D** IHC staining of CCT2 in primary breast cancer tissues (*n = 26*) and non-tumor tissues (*n = 26*) based on Qilu cohort. Scale bar = 100 μm. **E** the RNA and protein expression of CCT2 was upregulated in most breast cancer cells compared with that in normal cell (MCF-10A). **F**, **G** The correlation between CCT2 expression and overall survival or disease-free survival in breast cancer tissues based on Qilu cohort was calculated with the log-rank test. **H** CCT2 expression levels at different tumor stages of breast cancer cohorts in the metabric database (Stage1:*n* = 371, Stage2:*n* = 571, Stage3:*n* = 90, Stage4:*n* = 10). **I** CCT2 expression levels at different grades of breast cancer cohorts in the metabric database (G1:*n* = 170, G2:*n* = 770, G3:*n* = 592). (*P < 0.05, **P < 0.01, ***P < 0.001).

We initially investigated CCT2 expression in human breast cancer using publicly available datasets. Analysis of the Gene Expression Omnibus series revealed consistently elevated CCT2 expression in human breast cancer tissues compared with normal breast tissues (Fig. 1A). Furthermore, increased CCT2 expression was observed in several other cancers (supplementary Fig. 1A), including colon cancer (COAD), large B-cell lymphoma (DLBC), glioblastoma (GBM), pancreatic cancer (PAAD), thymoma (THYM), and uterine carcinosarcoma (UCS). We subsequently assessed CCT2 expression in a cohort of human breast cancer tissues obtained from Qilu Hospital, alongside normal tissues. Our analysis revealed that, compared with normal tissues, breast cancer tissues presented greater CCT2 mRNA expression (Fig. 1B). Moreover, we observed elevated mRNA expression levels of CCT2 in breast cancer tissues compared with their paired normal counterparts (Fig. 1C). Consistent with these findings, IHC staining revealed upregulated protein expression of CCT2 in breast cancer tissues relative to normal tissues within the Qilu cohort (Fig. 1D). Additionally, compared with normal cells (MCF-10A), most breast cancer cells exhibited higher CCT2 expression (Fig. 1E), providing additional evidence for the oncogenic function of CCT2 in breast cancer. We also assessed the clinical relevance of CCT2 expression, revealing a correlation between elevated CCT2 expression and worse overall survival (OS) and recurrence-free survival (RFS) in the Qilu cohort (Fig. 1F, G). Similarly, analysis of publicly available gene expression datasets demonstrated that increased CCT2 expression was correlated with inferior OS, RFS, and distant metastasis-free survival (DMFS) (Supplementary Fig. 1B–D). Additionally, elevated CCT2 levels were significantly associated with more advanced stages and higher histological grades (Fig. 1H, I).

Further evaluation of the clinical significance of CCT2 expression was conducted on the basis of the clinicopathological characteristics of breast cancer patients, with a focus on OS (supplementary Table S3). The results revealed significant associations between CCT2 expression and lymph node (LN) metastasis and distant metastasis in patients with breast cancer, although CCT2 expression was not significantly associated with age, tumor size, histological grade, ER status, PR status, HER2 status, or Ki67 expression. Univariate and multivariate Cox regression analyses confirmed that >3 LN metastases and CCT2 expression were independent prognostic indicators for overall survival in patients with breast cancer (supplementary Table S4). In summary, these findings collectively demonstrate that CCT2 expression is elevated in breast cancer and is associated with poor prognosis.

### CCT2 promotes the malignant progression of breast cancer cells in vitro

**Fig. 2:**
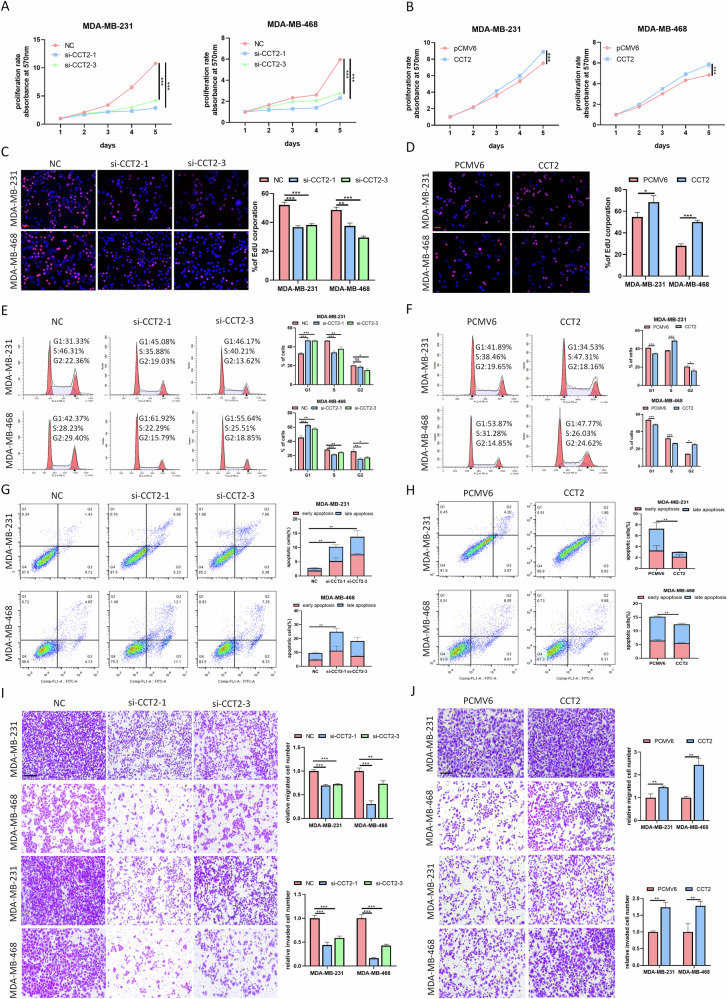
CCT2 promotes breast cancer cells malignant progression in vitro. MDA-MB-231 and MDA-MB-468 were transfected with siCCT2 or CCT2 plasimd for functional experiments. The effect of CCT2 on cell proliferation was evaluated by MTT **A**, **B** and EdU assay **C**, **D**. Scale bar = 100 μm. **E**, **F** cell cycle distributions in CCT2 knockdown or overexpressing cells were presented by flow cytometry. **G**, **H** Flow cytometry apoptosis analysis of CCT2 knockdown or overexpressing cells. **I**, **J** cell migration and invasion abilities of CCT2 knockdown or overexpressing cells were evaluated by the transwell assays. Scale bar =200μm. (*P < 0.05, **P < 0.01, ***P < 0.001).

To further substantiate the oncogenic role of CCT2 in breast cancer, we investigated its biological functions by transfecting breast cancer cells with CCT2 overexpression plasmids and siRNAs. The efficiency of gain/loss-of-function was verified at the RNA and protein levels (supplementary Fig. 2A, B). Subsequently, we observed that CCT2 knockdown markedly inhibited cell proliferation, whereas CCT2 overexpression promoted cell proliferation, as evidenced by the results of the MTT, EdU, and colony formation assays (Fig. 2A–D, supplementary Fig. 2C, D). Cell cycle analysis revealed that CCT2 knockdown induced cell cycle arrest at the G1 phase, whereas CCT2 overexpression increased the frequency of cells in the S or G2 phase (Fig. 2E, F). Flow cytometry assays demonstrated that CCT2 silencing markedly increased the cell apoptosis rate, whereas CCT2 overexpression had the opposite effect (Fig. 2G, H). Western blot analysis consistently revealed that CCT2 upregulation increased the protein levels of cell cycle-related proteins and suppressed the levels of apoptotic-related proteins, whereas CCT2 knockdown had the opposite effect (Supplementary Fig. 2G, H). To evaluate the impact of CCT2 on the metastatic potential of breast cancer cells, we conducted transwell and wound-healing assays. The results indicated that knockdown of CCT2 significantly inhibited cell migration and invasion, whereas overexpression of CCT2 increased these abilities (Fig. 2I, J, Supplementary Fig. 2E, F). As epithelial-mesenchymal transition (EMT) is closely associated with cell migration and invasion, we assessed the effect of CCT2 on the expression of EMT markers. Western blot assays revealed that CCT2 knockdown decreased the expression of mesenchymal markers (N-cadherin and vimentin) and increased the expression of an epithelial marker (E-cadherin). Conversely, CCT2 overexpression upregulated N-cadherin and vimentin while downregulating E-cadherin expression (Supplementary Fig. 2I, J). Immunofluorescence analysis further confirmed the alterations in E-cadherin and N-cadherin expression at the protein level (Supplementary Fig. 2K, L). Taken together, our findings demonstrate that CCT2 promotes oncogenic behavior in breast cancer cells.

### CCT2 activates the JAK2/STAT3 signaling pathway to promote the malignant progression of breast cancer cells

We subsequently investigated the mechanisms underlying the CCT2-mediated promotion of breast cancer progression. To identify differentially expressed genes between CCT2-knockdown cells and control cells, we performed RNA sequencing. Gene Ontology and Kyoto Encyclopedia of Genes and Genomes analyses revealed that the differentially expressed genes were associated with focal adhesion and the JAK/STAT signaling pathway(supplementary Fig. 3A). Notably, the JAK2/STAT3 signaling pathway is known to play a crucial role in tumorigenesis and development [17]. Therefore, we examined the expression of key proteins in the JAK2/STAT3 pathway. As depicted in supplementary Fig. 3B, CCT2 upregulation markedly increased, while CCT2 knockdown greatly decreased, the protein levels of p-STAT3 and p-JAK2, with no obvious effects on the total expression of STAT3 and JAK2. Moreover, we utilized the JAK2/STAT3-specific inhibitor WP1066 to determine whether activation of this pathway is essential for the CCT2-mediated progression of breast cancer cells. Through MTT, EdU, flow cytometry, and transwell assays, we observed that WP1066 attenuated the pro-proliferative, anti-apoptotic, pro-migrative and invasive effects of CCT2 (supplementary Fig. 3C–G). Western blot analysis further demonstrated that WP1066 inhibited the activity of p-JAK2 and p-STAT3 and reduced the expression of cell cycle-related and EMT-related markers (Supplementary Fig. 3H). These findings collectively confirm that CCT2 promotes the progression of breast cancer cells by activating the Jak2/STAT3 signaling pathway.

### Mutual binding between CCT2 and Trim21 in breast cancer cells

**Fig. 3:**
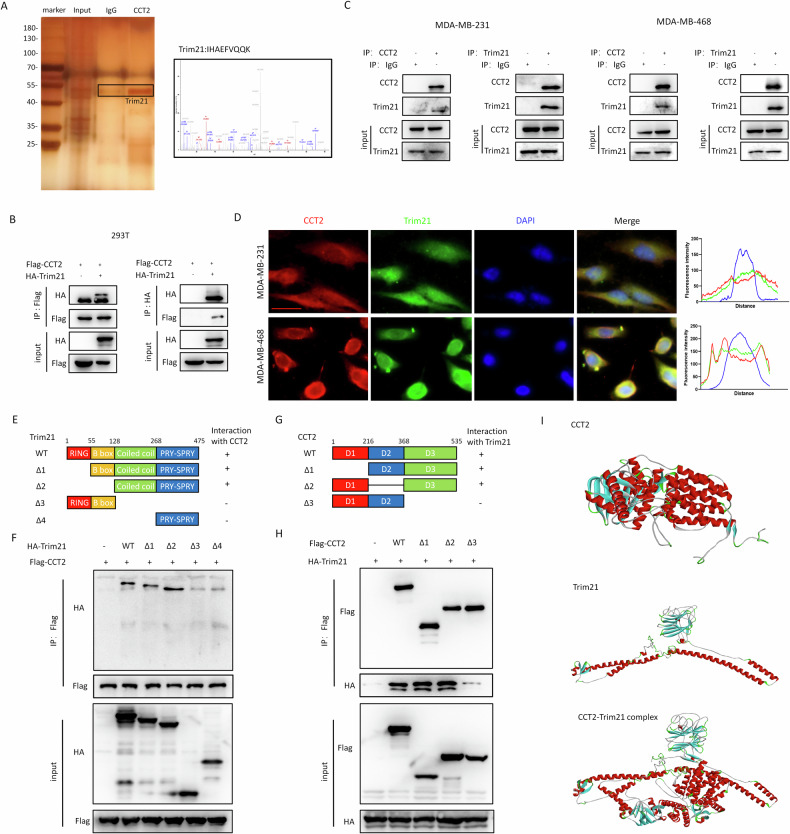
Mutual binding between CCT2 and Trim21 in breast cancer cell. **A** CCT2-Flag plasmid was transfected into MDA-MB-231 cells for CO-IP. The protein pulled down were analyzed by silver staining. The images of silver staining were displayed. Trim21 was identified as a binding partner of CCT2, and the representative spectra were included. **B** HEK293T cells were transfected with CCT2-Flag and Trim21-HA, after which immunoprecipitation (IP) was performed to assess interaction between CCT2 and Trim21 using anti-Flag or anti-HA antibodies. **C** validation of biochemical interaction between CCT2 and Trim21 in MDA-MB-231 and MDA-MB-468 cells by coimmunoprecipitation of endogenous CCT2 and Trim21. **D** Co-localization of CCT2 and Trim21 in MDA-MB-231 and MDA-MB-468 cells were examined by using a fluorescence microscope. Scale bar=20μm. **E** schematic diagram of human Trim21 domains and strategy to engineer a series of Trim21 deletion mutants. **F** The interactions between CCT2 and Trim21 fragments were examined by coimmunoprecipitation experiments in HEK293T cells. **G** Schematic diagram of human CCT2 domains and strategy to engineer a series of CCT2 deletion fragments. **H** The interactions between Trim21 and CCT2 fragments were examined by coimmunoprecipitation experiments in HEK293T cells. **I** I-TASSER was used to generate 3D structures for CCT2 and Trim21, the CCT2-Trim21 complex was predicted using ZDOCK.

To identify potential regulators of CCT2 protein abundance, we employed tandem affinity purification coupled with mass spectrometry analysis. Our results revealed an interaction between CCT2 and the ubiquitin E3 ligase Trim21 (Fig. 3A). This interaction was further validated in HEK293T cells (Fig. 3B) and endogenously in MDA-MB-231, MDA-MB-468 and 4T1 breast cancer cells (Fig. 3C, supplementary Fig. 4A). Immunofluorescence colocalization analysis further supported the interaction between CCT2 and Trim21 (Fig. 3D). To delineate the domains responsible for the CCT2-Trim21 interaction, we generated a series of Flag-tagged CCT2 and HA-tagged Trim21 truncation mutants. Trim21 comprises a RING finger, B-box, coiled-coil, and PRY/SPRY domains (Fig. 3E). To determine which of these domains enable Trim21 to interact with CCT2, a series of Trim21 truncation mutants were generated (Fig. 3E). Through molecular mapping, we identified amino acid residues 128-268 on Trim21(coiled-coil domain) as crucial region for mediating its binding with CCT2 (Fig. 3F). Similarly, mapping of CCT2 revealed amino acid stretches 368-535 as the region facilitating interaction with Trim21 (Fig. 3G, H). Three-dimensional models of CCT2, Trim21, and their complexes are depicted in Fig. 3I.

### Trim21 mediates the ubiquitination and degradation of CCT2

**Fig. 4:**
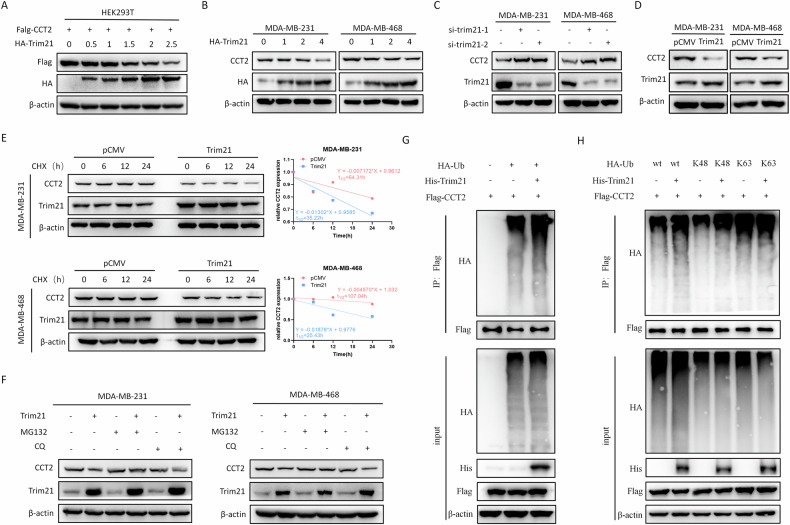
Trim21 mediates the ubiquitination and degradation of CCT2. **A**, **B** Immunoblotting was performed to analyze lysates prepared from HEK293T cells **A**, MDA-MB-231 and MDA-MB-468 **B** following transfection with Flag-CCT2 and different doses of the HA-Trim21 expression plasmid. **C**, **D** Immunoblotting were performed to evaluate the expression of CCT2 in Trim21-silenced **C** or Trim21- overexpressing **D** breast cancer cells. **E** MDA-MB-231 and MDA-MB-468 cells were transfected with indicated plasmid, followed by cycloheximide (CHX) treatment for indicated duration. Immunoblotting was then performed using lysates prepared from these cells, with the ImageJ software being used to quantify CCT2 expression and β-actin being used for normalization. **F** Following transfection with the CCT2 and Trim21 plasmids and treatment with CQ, and MG132 for 4 h, breast cancer cell lysates were subjected to western blotting. **G** Anti-flag was used to immunoprecipitated lysates prepared from 293 T cells following transient HA-Ub, Flag-CCT2 and His-Trim21 co-transfection, after which anti-HA was used for immunoblotting. **H** immunoprecipitation analyses were performed for lysates from 293 T cells following the transient co-transfection of Flag-CCT2, His-Trim21, and total-Ub or K48-Ub or K63-Ub mutant. (*P < 0.05, **P < 0.01, ***P < 0.001).

The overexpression of Trim21 in HEK293T, MDA-MB-231 and MDA-MB-468 cells caused a dose-dependent reduction in the CCT2 protein level (Fig. 4A, B, Supplementary Fig. 5A, B). Conversely, reduced Trim21 expression resulted in elevated CCT2 protein levels, whereas Trim21 overexpression had the opposite effect without affecting CCT2 mRNA levels (Fig. 4C, D, supplementary Fig. 5C–E). These results were further confirmed in 4T1 cells (supplementary Fig. 4B). Our results clearly suggest that Trim21 primarily affects posttranslational modifications rather than the mRNA transcription of CCT2. Moreover, CHX chase assays demonstrated that Trim21 overexpression promoted CCT2 protein degradation in breast cancer cells (Fig. 4E), whereas Trim21 knockdown extended the half-life of the CCT2 protein (supplementary Fig. 5F). The proteasome inhibitor MG132 effectively reversed Trim21-induced CCT2 degradation, whereas chloroquine (CQ), an inhibitor of lysosomal activity and the autophagy pathway, had no effect on degradation (Fig. 4F, Supplementary Fig. 4C). These results imply that Trim21 likely mediates the ubiquitination and subsequent degradation of the CCT2 protein. Trim21 expression increased the degree of CCT2 ubiquitination in the environment of MG132, but Trim21 silencing decreased the degree of CCT2 ubiquitination, as shown by the results of the ubiquitination experiments (Fig. 4G, Supplementary Fig. 5G). Moreover, we examined the specific ubiquitin chain linkages induced by Trim21 on CCT2 by using K48 and K63 ubiquitin mutant isoforms. The results indicated that Trim21 significantly enhanced the K48-linked polyubiquitination of CCT2 but had no effect on K63-linked polyubiquitination (Fig. 4G). Additionally, deletion of Trim21 selectively inhibited the K48-linked polyubiquitination of CCT2 but not affect K63-linked polyubiquitination (Supplementary Fig. 5H). These findings suggest that Trim21 promotes K48-polyubiquitination and subsequent degradation of CCT2.

### Trim21 suppresses the oncogenic properties of breast cancer cells in a CCT2-dependent manner

**Fig. 5:**
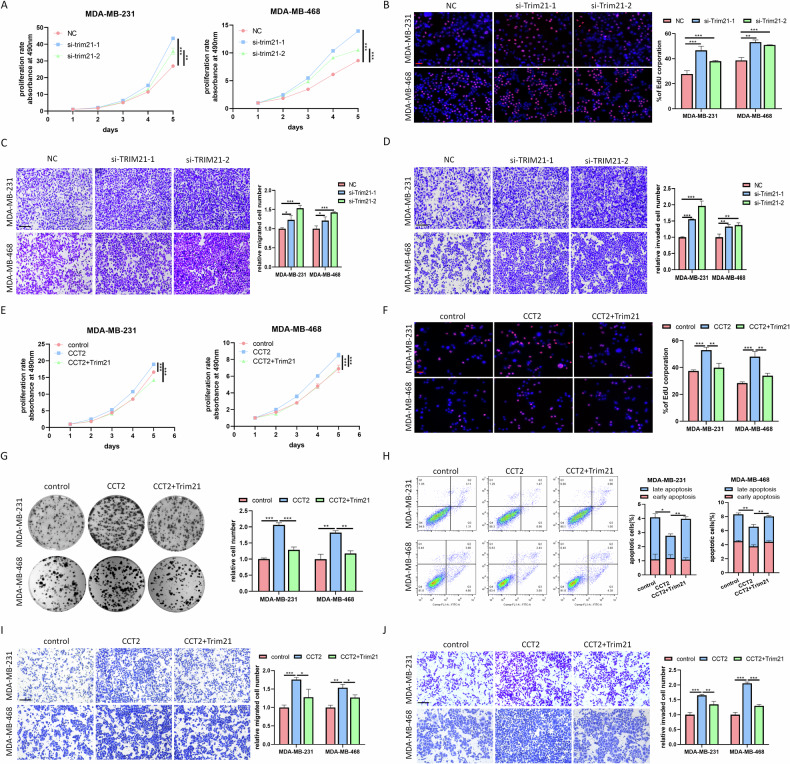
Trim21 suppresses oncogenic properties of breast cancer cells through a CCT2 dependent manner. MTT **A** and EdU **B** assays indicated the increased proliferative ability in breast cancer cells transfected with Trim21 siRNAs. Scale bar = 100 μm.The migration **C** and invasion **D** capabilities of breast cancer cells were promoted by Trim21 knockdown. Scale bar = 200 μm. Breast cancer cells were transfected with CCT2 and Trim21 alone or simultaneously. Then the ability of cell proliferation, apoptosis, migration, and invasion was respectively assessed by MTT **E**, EdU **F**, colony assay **G**, flow cytometry assay **H**, and transwell assays **I**, **J**. (*P < 0.05, **P < 0.01, ***P < 0.001).

To assess the impact of Trim21 on breast cancer cell function, we first analyzed several publicly available datasets, and revealed that elevated Trim21 expression was associated with better OS, RFS, and DMFS in breast cancer patients (Supplementary Fig. 6A–C). The knockdown of Trim21 resulted in an increased proliferation rate and a decreased apoptotic rate in breast cancer cells (Fig. 5A, B, supplementary Fig. 6D, E). Additionally, Trim21 knockdown enhanced cell migration and invasion (Fig. 5C, D). Importantly, functional assays revealed that Trim21 overexpression not only countered CCT2-induced pro-proliferative and anti-apoptotic effects but also mitigated CCT2-induced pro-migrative and invasive abilities (Fig. 5E–J). These findings collectively suggest that Trim21 suppresses the oncogenic properties of breast cancer cells through a CCT2-mediated mechanism.

### Loss of CCT2 impedes breast cancer growth and promotes the CD4^+^ T cells activation

**Fig. 6:**
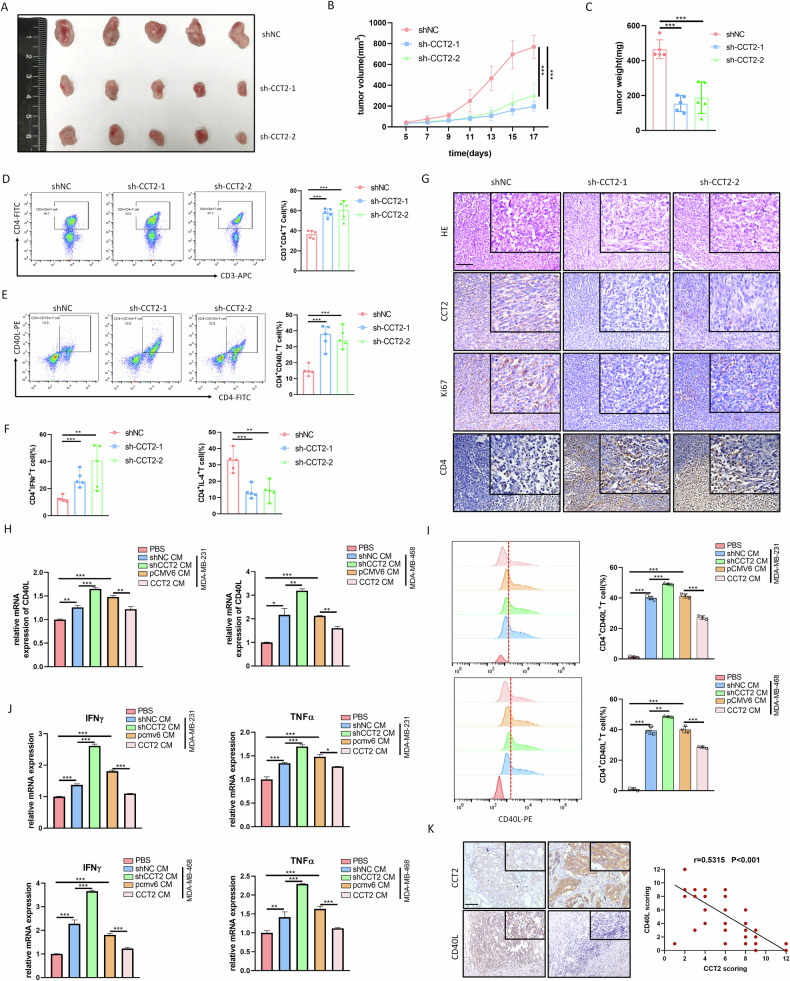
Loss of CCT2 impedes breast cancer growth and promotes the CD4^+^T cells activation. **A** Tumors were obtained from 4T1-shCCT2 and 4T1-shNC group mice sacrificed on day 17(n = 5, Scale bar =1 cm). **B** The tumor volumes were measured every two days. **C** Tumor weights were recorded after sacrifice of the mice. Flow cytometry analysis of CD3^+^CD4^+^T cells **D**, CD3^+^CD4^+^CD40L^+^T cells **E**, CD3^+^CD4^+^IFNγ^+^T cells and CD3^+^CD4^+^IL-4^+^T cells **F** from 4T1 tumors harvested from mice in three different groups (*n* = 5). **G** H&E staining showed the tissue morphology of transplanted tumors. Representative pictures of IHC staining of CCT2, Ki67, and CD4 in the tumor tissues. Scale bar =100μm. qPCR (**H**) and flow cytometry **I** analysis were used to detect CD40L expression on CD4^+^T cells under different conditions: normal media, shCCT2 or shNC breast cancer cell CM, and pCMV6 or CCT2 overexpressing breast cancer cell CM. **J** the levels of mRNA expression of IFNγ and TNFα in CD4^+^T cells from each group were determined by qPCR. **K** Immunohistochemical staining depicted the correlation between the expression of CCT2 and CD40L in breast cancer tissues (*N* = 93) based on Qilu cohort (*P < 0.05, **P < 0.01, ***P < 0.001).

We further investigated the function and regulatory mechanism of CCT2 in the TME of breast cancer using a tumor-bearing model. 4T1-sh-CCT2 or 4T1-shNC cells were generated via lentiviral-mediated transfection and subcutaneously injected into the flanks of BALB/c mice randomly. The knockdown efficiency of sh-CCT2 was confirmed by qPCR (supplementary Fig. 7A). The tumors in the shCCT2 group were significantly smaller and lighter in volume than those in the shNC group were, indicating that the knockdown of CCT2 in breast cancer cells delayed tumor growth in vivo (Fig. 6A–C). Next, we assessed the impact of CCT2 suppression on the quantity of immune cells that infiltrate tumors. Flow cytometry analysis revealed a significant increase in the quantity of CD4^+^ T cells among all the immune cells analyzed in the 4T1-shCCT2 tumors (Fig. 6D) but not in the number of CD8^+^ T cells, tumor-associated macrophages (TAMs), or myeloid-derived suppressor cells (MDSCs) upon CCT2 suppression (supplementary Fig. 7B–E). Furthermore, we assessed the activation and cytokine secretion of CD4^+^ T cells in tumors. The results showed that shCCT2 enhanced the expression of CD40L in CD4^+^ T cells (Fig. 6E). Moreover, the intracellular expression of IFNγ (CD3^+^CD4^+^IFNγ^+^) was significantly increased, whereas the expression of IL-4 (CD3^+^CD4^+^IL-4^+^) was significantly reduction in the shCCT2 group (Fig. 6F). IHC staining further confirmed that CCT2 knockdown suppressed ki67 expression while enhancing CD4 expression within xenograft tumors (Fig. 6G).

With the aid of CD4^+^ T cell separation kit, CD4^+^ T cells were separated from PBMCs and refined to better clarify the impact of CCT2 on the activation of CD4^+^ T cells and the generation of cytokines in vitro. The purity was greater than 97%, as shown by flow cytometry (supplementary Fig. 7F). We then exposed CD4^+^ T cells to culture medium (CM) from CCT2-knockdown or CCT2-overexpressing breast cancer cells to compare their ability to modulate the T-cell response. shCCT2 CM dramatically raised CD40L expression on CD4^+^ T cells, whereas CCT2 CM had the opposite effect, according to the results of the qPCR and flow cytometry assays (Fig. 6H, I). Furthermore, we quantified the secretion of cytokines by CD4^+^ T cells. The findings demonstrated that in the CD4^+^ T cells, shCCT2 CM dramatically increased the expression of IFNγ and TNFα and reduced the expression of IL-4 compared with those in the shNC CM group (Fig. 6J). Notably, overexpression of CCT2 in breast cancer cells had the opposite effect (Fig. 6J, Supplementary Fig. 7G). Consistent with these findings, IHC analysis further verified increased infiltration of CD40L-positive immunocytes in CCT2-low-expressing breast cancer tissues (Fig. 6K). Spearman Correlation analysis revealed a negative correlation between CCT2 and CD40L expression (Fig. 6K). These findings indicate that CCT2 regulates CD40L expression and cytokine production, which negatively influences CD4^+^ T cells activity and plays a critical role in the evolution of breast cancer.

### Exosomal CCT2 suppresses CD4^+^ T cells activation and inflammatory cytokine production

**Fig. 7:**
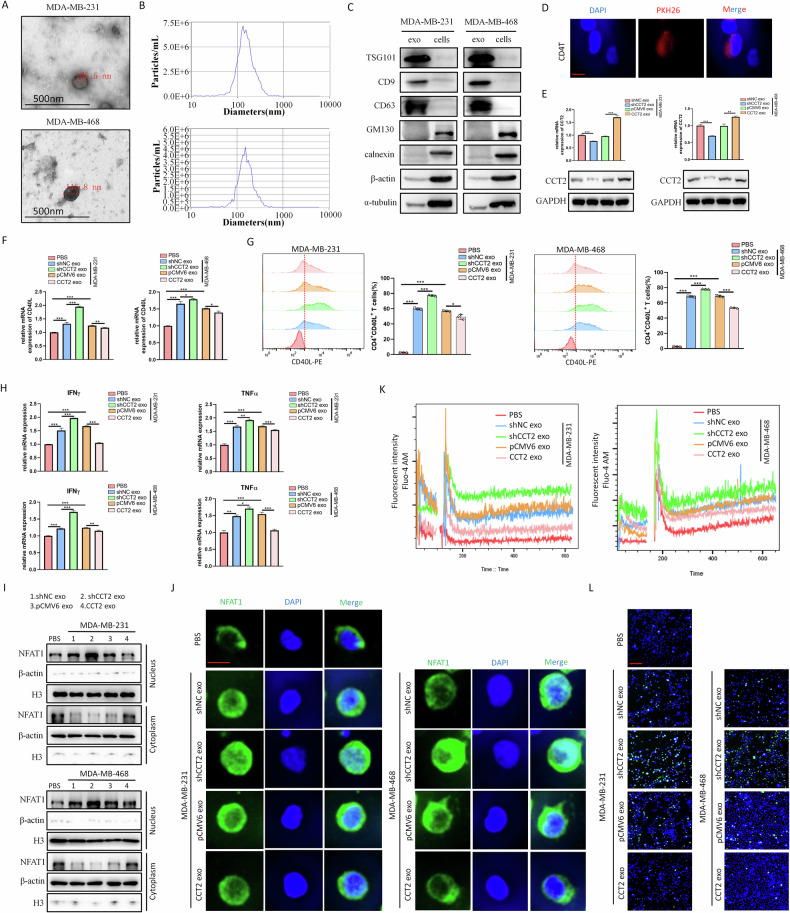
Exosomal CCT2 suppresses CD4^+^T cells activation and inflammatory cytokine production. Characterization of harvested breast cancer exos. **A** Transmission electron microscope was used to detect the spheroid morphology and size of breast cancer exos. Scale bar = 500 nm. **B** Nanoparticle tracking analysis was performed to detect the diameter quantitation of breast cancer exos. **C** Western blot analysis of exosomal markers in the exos isolated from breast cancer cells. **D** A representative fluorescence image of the internalization of fluorescently labeled breast cancer exos in CD4^+^T cells. Scale bar = 5 μm. **E** CCT2 expression of CD4^+^T cells treated with exos released by indicated cells were detected by qPCR and western blot. qPCR **F** and flow cytometry **G** analysis were used to detect CD40L expression on CD4^+^T cells under different conditioned breast cancer exos: PBS, shCCT2 or shNC breast cancer cell exos, and pCMV6 or CCT2 overexpressing breast cancer cell exos. **H** the levels of mRNA expression of IFNγ and TNFα in CD4^+^T cells from each group were determined by qPCR. **I** The expression levels of NFAT1 in the cytosolic and nuclear fractions of CD4^+^T cells from each group were detected by western blot. **J** The location of NFAT1 (fluorescent green) in CD4^+^T cells from each group were visualized using a fluorescent microscope. The nucleus was stained with DAPI (fluorescent blue). Scale bar =5μm. **K** Representative curves of Ca^2+^ influx dynamics in CD4^+^T cells from each group were depicted using Fluo-4AM fluorescence. **L** The fluorescence of Fluo-4AM (fluorescent green) was investigated to measure the level of Ca^2+^ influx in CD4^+^T cells from each group. Scale bar = 40 μm. (*P < 0.05, **P < 0.01, ***P < 0.001).

Since tumor culture medium affects CD4^+^ T cells activation, we explored the exact mediators that are responsible for this effect via fractionation of the components of the breast cancer cell culture media. Following ultracentrifugation (supplementary Fig. 8A), we found that different treatment supernatants containing cytokines and metabolites had no significant effects on CD4^+^ T cells activation (Supplementary Fig. 8B). Therefore, we hypothesized that breast cancer cells primarily suppress CD4^+^ T cells activation through exosomal forms. Exosomes (Exos) were harvested from stable CCT2-knockdown or CCT2-overexpressing breast cancer cell culture medium via ultracentrifugation and characterized using nanoparticle tracking analysis (NTA), transmission electron microscopy (TEM) and western blot analysis. Round particles covered in a bilayer membrane that resembled exosomes were observed via TEM (Fig. 7A). These vesicles were between 30 and 150 nm in size (Fig. 7B). The presence of exo-related markers, such as TSG101, CD9, and CD63, and the absence of cellular markers, such as GM130, calnexin, β-actin, and α-tubulin, were verified by western blot analysis (Fig. 7C). PKH26-labled exos were cultured with CD4^+^ T cells for 6 h to determine whether breast cancer cell exos could be taken up by CD4^+^ T cells. The results revealed that breast cancer exos were internalized by CD4^+^ T cells, with red fluorescent spots observed in the cytoplasm (Fig. 7D). Furthermore, compared with that in the control group, the expression of CCT2 in CD4^+^ T cells decreased following treatment with exos derived from CCT2-knockdown breast cancer cells, whereas CCT2-overexpressing exos had the opposite effect (Fig. 7E). Subsequent qPCR and flow cytometry analyses revealed that the shCCT2 exo group presented considerably greater CD40L expression on CD4^+^ T cells than the shNC exo group did (Fig. 7F, G). Additionally, compared with shNC exos, CD4^+^ T cells treated with shCCT2 exos expressed more IFN-γ and TNF-α and less IL-4 (Fig. 7H, supplementary Fig. 8C). These findings were further confirmed via intracellular cytokine analysis using flow cytometry, which showed that shCCT2 exo promoted IFN-γ production and inhibited IL-4 production in CD4^+^ T cells (supplementary Fig. 8D, E). Conversely, CCT2 exos had the opposite effects (Fig. 7F–H, supplementary Fig. 8D, E).

**Fig. 8:**
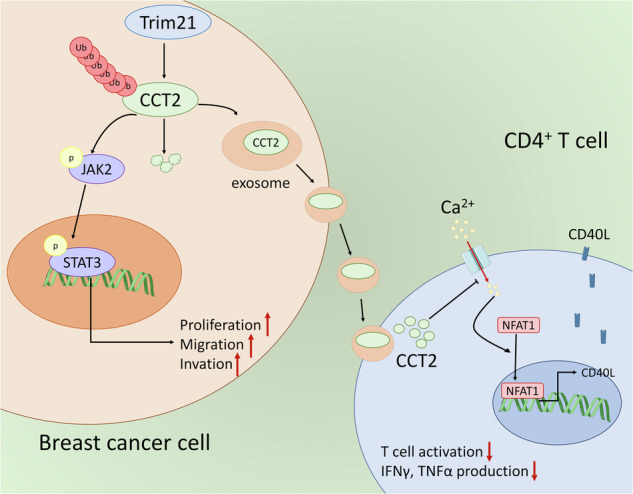
Schematic model on the proposed role of CCT2 in breast cancer progression and immune evasion. CCT2 promotes breast cancer tumorigenesis through JAK2/STAT3 signaling pathway. Trim21, functioning as an E3 ligase, interacts with CCT2 and promotes its ubiquitination and degradation, partially counteracting the pro-tumor effect of CCT2. Furthermore, exosomal CCT2 derived from breast cancer cells suppresses the activation and pro-inflammatory cytokine secretion of CD4^+^T cell through modulating Ca^2+^-NFAT1 signaling.

Additionally, we investigated the possible mechanism by which CD4^+^ T cells activation was regulated by CCT2, which was derived from breast cancer cell exos. Previous studies demonstrated that the ability of STAT5 to bind the CD40L transcriptional promoter or regulate NFAT1 signaling was necessary for activation-induced CD40L expression on CD4^+^ T cells [18, 19]. Western blot analysis revealed that the expression of p-STAT5 did not significantly change in the shCCT2 exos- or CCT2 exos-treated groups compared with the control exos-treated groups (Supplementary Fig. 8F). However, we observed increased nuclear translocation of NFAT1 in CD4^+^ T cells in the shCCT2 exos group than in those in the shNC exos group, which was inhibited by CCT2 exos treatment (Fig. 7I). Immunofluorescence analysis confirmed these results (Fig. 7J). NFAT1 is activated by the calcineurin-dependent pathway, which is sensitive to changes in the intracellular Ca^2+^ concentration [20, 21]. Therefore, we explored whether breast cancer cell exos-derived CCT2 regulated Ca^2+^ influx in CD4^+^ T cells. Fluo-4AM fluorescent dye was used to measure the intracellular Ca^2+^ concentration, revealing that shCCT2 exos markedly increased the intracellular Ca^2+^ content of CD4^+^ T cells, while CCT2 exos decreased Ca^2+^ influx (Fig. 7L, supplementary Fig. 8G). The flow cytometry results further validated these findings (Fig. 7K). These results were further confirmed in mouse breast cancer cells (4T1) (supplementary Fig. 9A–E). Taken together, the results demonstrate that breast cancer cell exos-derived CCT2 inhibits CD4^+^ T cells activation by limiting CD40L expression via the Ca^2+^-NFAT1 signaling pathway. On the basis of these collective data, we conclude that CCT2 is essential for tumor development, metastasis, and immune evasion (Fig. 8).

## Discussion

Breast cancer, characterized by genetic alterations governing cell growth and proliferation, remains a formidable challenge in women’s health and is the most prevalent cancer globally with diverse molecular subtypes and clinical manifestations. Despite therapeutic advancements, numerous lives continue to be claimed, emphasizing the need for a deeper understanding of its molecular intricacies to enhance prognostication and therapeutic strategies.

CCT2, an isoform of heat shock protein 60 in eukaryotic cells, is highly expressed in numerous malignant tissues and plays a role in cell metabolism. CCT2 is strongly linked to the development, prognosis and genesis of tumors [22–24]. We used a variety of datasets and a dedicated cohort, which included tissues from our cohort and more evidence of higher CCT2 expression in breast cancer tissues, to confirm our findings. Furthermore, elevated levels of CCT2 were correlated with worse prognoses in breast cancer patients. Subsequent findings revealed the role of CCT2 in fostering breast cancer proliferation, migration, and invasion, highlighting its importance in cancer progression. Central to cell signaling, the JAK2/STAT3 pathway assumes paramount importance in tumorigenesis, orchestrating invasion and metastasis processes [17]. In breast cancer, this pathway is pivotal for the development of human CD44^+^CD24^-^ stem cell-like cancer cells, and the inhibition of JAK2 prevents xenograft growth [25]. The EMT and metastasis of human breast cancer cells are positively linked with abnormal JAK2/STAT3 signal activation [25, 26]. Consistent with these observations, our study revealed increased levels of p-JAK2 and p-STAT3 in CCT2-overexpressing breast cancer cells, indicating that CCT2 drives breast cancer progression via JAK2/STAT3 pathway activation.

The ubiquitination-deubiquitination process of proteins play a crucial role in modulating protein dynamics, impacting their location, stability, activity, and interactions with other proteins [27, 28]. Depending on their substrate specificity, E3 ligases can significantly affect this process as tumor suppressors or oncogenes [27, 29–31]. While our investigation did not detect a notable increase in CCT2 mRNA levels upon Trim21 overexpression, our findings suggested that Trim21 likely regulated CCT2 through posttranslational modifications. Remarkably, breast cancer cells overexpressing Trim21 exhibited accelerated degradation of the CCT2 protein upon treatment with CHX, implicating Trim21 in modulating CCT2 stability. We established an interaction between CCT2 and Trim21, with Trim21 positively regulating CCT2 ubiquitination. Moreover, the presence of seven lysine(K) residues in ubiquitin can result in different biological outcomes depending on the specific linkages that are formed [32]. The most common ubiquitin mutant isoforms are K48 and K63. K48-linked polyubiquitin chains typically target proteins for degradation, whereas K63-linked polyubiquitin chains serve as scaffolds for signaling or protein-protein interactions [33]. Intriguingly, our exploration unveiled that Trim21 only regulates the K48-linked ubiquitination of CCT2 and has no influence on K63-linked ubiquitination. These findings delineate a novel regulatory mechanism governing CCT2 ubiquitination and degradation, underscoring the potential of Trim21-mediated CCT2 modulation as a promising therapeutic target across multiple diseases.

Immunotherapy and cancer immunology are pivotal areas of research in oncology, with a recent emphasis on understanding the role of CD8^+^ T cells and the tumor microenvironment. However, the importance of CD4^+^ T cells, known for their role as central players in coordinating innate and antigen-specific immune responses, is increasingly recognized [13, 34]. Furthermore, CD4^+^ T cells have emerged as powerful independent antitumor effector cells [35, 36]. To produce antitumor effects in vivo, tumor-associated antigen-specific CD4^+^ T cells activation is necessary [37–39]. These findings emphasize the need for additional exploration and development of CD4^+^ T cells biology. The membrane glycoprotein CD40L, which belongs to the tumor necrosis factor superfamily, has a molecular weight between 32 and 39 kDa [40]. Its expression significantly rises on activated CD4^+^ T cells, where it binds to and activates the adaptive and innate immune systems via interaction with its cognate receptor CD40 [41]. In this mechanism, CD4^+^ T cells expressing CD40L activate primarily classical type 1 DCs (cDC1) for CD8^+^ T cell priming [42]. Both CD40 and MHC class II on these cDC1s are required to receive these CD4^+^T cell for efficient CD8^+^ T cell priming during antitumor immune control in mice [42]. Interestingly, the CD40-CD40L interaction is also crucial for B cell activation by CD4^+^ T cells, particularly CD4^+^ follicular helper T cells [43]. CD40-activated B cells subsequently stimulate cytotoxic T cell responses. Thus, CD4^+^ T cell help via CD40L supports the priming of antitumor immune responses, possibly both by DCs and B cells [13]. In our study, we observed that CCT2 silencing in breast cancer cells led to enhanced activation (CD40L) and the proinflammatory cytokine (IFN-γ and TNFα) production in CD4^+^ T cells within the tumor microenvironment. Notably, the activation of T cells can occur through classical antigen-presenting cell-induced immunostimulatory mechanisms as well as through a distinct “direct exosome interaction” [44]. Our investigation revealed the presence of CCT2 in breast cancer cell-derived exosomes, which contributed to the suppression of CD4^+^ T cells activation and proinflammatory cytokine secretion. Furthermore, we delineated the potential mechanism underlying CD4^+^ T cells activation. Previous research has shown that the activation of NFAT1 signaling is primarily responsible for the elevated expression of CD154 on CD4^+^ T cells in systemic lupus erythematosus [45]. NFAT1 is an essential transcription factor that controls T cell activation, differentiation, fatigue, and self-tolerance [46, 47]. Dephosphorylation activates NFAT1, which then translocates to the nucleus to cause the production of effector cytokines and T cell activation markers [47]. Here, we demonstrated that breast cancer derived shCCT2 exosomes promoted NFAT1 activation in CD4^+^ T cells, whereas CCT2 exosomes had the opposite effect. Importantly, NFAT1 activation is calcium-dependent, with increased cytoplasmic Ca^2+^ levels triggering NFAT1 nuclear translocation during the activation process of CD4^+^ T cells [48, 49]. Fascinatingly, the results of this study indicate that breast cancer derived shCCT2 exosomes promote the Ca^2+^ influx in CD4^+^ T cells, while CCT2 exosomes inhibit Ca^2+^ influx. Together, these results suggest that CCT2 in breast cancer cells-derived exosomes modulate CD4^+^ T cells activation through the Ca^2+^-NFAT1 pathway, ultimately compromising antitumor immunity.

In conclusion, our research elucidates the critical function of CCT2 in breast cancer progression, primarily via the initiation of the JAK2/STAT3 signaling cascade. Additionally, we demonstrated that Trim21 modulates CCT2 ubiquitination and degradation, thereby influencing its impact on breast cancer progression. Moreover, we revealed that CCT2 contributes to breast cancer immune evasion by packaging into breast cancer cell-derived exosomes, where it regulates CD4^+^ T cell activation through the Ca^2+^-NFAT1 pathway. These insights into the function and mechanism of CCT2 highlight its potential as a therapeutic target for breast cancer.

### Supplementary information

Supplemental Material

supplementary WB

## Data Availability

Source data and reagents are available from the corresponding author upon reasonable request.
