# Supplementary material for: Trim21-mediated CCT2 ubiquitination suppresses malignant progression and promotes CD4+T cell activation in breast cancer
Source: Cell Death Dis. 2024 Jul 30;15(7):542. doi: 10.1038/s41419-024-06944-8 (PMC11289294; doi:10.1038/s41419-024-06944-8)
Supplement: Supplementary file 1 — Supplemental Material [file 41419_2024_6944_MOESM1_ESM.docx]

**Supplementary tables**

**Supplementary Table 1**

Table 1. Primer sets used for qRT-PCR

| **Primer set** | **Primers** | **Sequence (5’-3’)** |
| --- | --- | --- |
| **CCT2(human)** | Forward | GCTCACAGTGAAGGCAATACCAC |
|  | Reverse | GCACTCAGAAGAACCTGTCGCT |
| **CCT2(mouse)** | Forward | TGACCAACGACGGTGCTACCAT |
|  | Reverse | ACAGAGGTAGTGCCATCACCAAC |
| **Trim21** | Forward | CAGAACTCAGGAGTGTGTGCCA |
|  | Reverse | TCCAAGCCTCACTTGTCTCCGA |
| **CD40L(human)** | Forward | GCGGCACATGTCATAAGTGAGG |
|  | Reverse | GTCCTTGTCTTTTAACGGTCAGC |
| **CD40L(mouse)** | Forward | GAACTGTGAGGAGATGAGAAGGC |
|  | Reverse | TGGCTTCGCTTACAACGTGTGC |
| **IFNγ** | Forward | GAGTGTGGAGACCATCAAGGAAG |
|  | Reverse | TGCTTTGCGTTGGACATTCAAGTC |
| **TNFα** | Forward | CTCTTCTGCCTGCTGCACTTTG |
|  | Reverse | ATGGGCTACAGGCTTGTCACTC |
| **IL-4** | Forward | CCGTAACAGACATCTTTGCTGCC |
|  | Reverse | GAGTGTCCTTCTCATGGTGGCT |
| **β-actin(human)** | Forward | GGGAAATCGTGCGTGACATT |
|  | Reverse | GGAACCGCTCATTGCCAAT |
| **β-actin(mouse)** | Forward | CATTGCTGACAGGATGCAGAAGG |
|  | Reverse | TGCTGGAAGGTGGACAGTGAGG |

**Supplementary Table 2**

Table 2. The antibodies used in this study

| **Antibody** | **Supplier (Catalog No.)** |
| --- | --- |
| CCT2 Rabbit Monoclonal antibody | Abcam (ab92746) |
| Trim21 Mouse Monoclonal antibody | Proteintech (67136-1-Ig) |
| BCL2 Rabbit Polyclonal antibody | Proteintech (12789-1-AP) |
| BAX Rabbit Polyclonal antibody | Proteintech (50599-2-Ig) |
| cyclinD1 Rabbit Polyclonal antibody | Proteintech (26939-1-AP) |
| CDK6 Rabbit Polyclonal antibody | Proteintech (14052-1-AP) |
| CDK4 Rabbit Polyclonal antibody | Proteintech (11026-1-AP) |
| N-Cadherin Rabbit Polyclonal antibody | Proteintech (22018-1-AP) |
| E-Cadherin Rabbit Monoclonal antibody | Proteintech (20874-1-AP) |
| Vimentin Rabbit Monoclonal antibody | Proteintech (60330-1-Ig) |
| MMP2 Rabbit Polyclonal antibody | Proteintech (10373-2-AP) |
| MMP9 Rabbit Polyclonal antibody | Proteintech (10375-2-AP) |
| ZEB1 Rabbit Polyclonal antibody | Proteintech (21544-1-AP) |
| HA tag Rabbit Polyclonal antibody | Proteintech (51064-2-AP) |
| FLAG Mouse Monoclonal antibody | Proteintech (66008-4-Ig) |
| His tag Mouse Monoclonal antibody | Proteintech (66005-1-Ig) |
| p-JAK1 Rabbit Monoclonal antibody | CST (74129S) |
| JAK1 Mouse Monoclonal antibody | Proteintech (66466-1-Ig) |
| p-JAK2 Rabbit Monoclonal antibody | CST (3776S) |
| JAK2 Rabbit Monoclonal antibody | CST (3230S) |
| p-STAT3 Rabbit Monoclonal antibody | CST (9145S) |
| STAT3 Rabbit Monoclonal antibody | CST (12640S) |
| p-STAT5 Rabbit Monoclonal antibody | CST (4322S) |
| STAT5 Rabbit Monoclonal antibody | CST (94205S) |
| NFAT1 Rabbit Polyclonal antibody | Proteintech (22023-1-AP) |
| H3 Rabbit Polyclonal antibody | Proteintech (17168-1-AP) |
| TSG101 Rabbit Polyclonal antibody | Proteintech (28283-1-AP) |
| CD9 Rabbit Polyclonal antibody | Proteintech (20597-1-AP) |
| CD63 Rabbit Polyclonal antibody | Proteintech (25682-1-AP) |
| GM130 Rabbit Polyclonal antibody | Proteintech (11308-1-AP) |
| Calnexin Rabbit Polyclonal antibody | Proteintech (10427-2-AP) |
| α-tubulin Rabbit Polyclonal antibody | Proteintech (11224-1-AP) |
| Ki67 Rabbit Polyclonal antibody | Proteintech (27309-1-AP) |
| CD4 Mouse Monoclonal antibody | Proteintech (67786-1-Ig) |
| CD154 Rabbit Polyclonal antibody | Proteintech (16668-1-AP) |
| β-actin Rabbit Polyclonal antibody | Affinity (AF7018) |
| APC anti-mouse CD3 antibody | Biolegend (100236) |
| PE anti-mouse CD8 antibody | Biolegend (100707) |
| FITC anti-mouse CD4 antibody | Biolegend (116003) |
| PE anti-mouse CD40L antibody | Biolegend (157003) |
| PE anti-mouse IL-4 antibody | Biolegend (504103) |
| PE/Cy7 anti-mouse IFNγ antibody | Biolegend (505825) |
| APC anti-mouse CD11b antibody | Biolegend (101212) |
| PE anti-mouse F4/80 antibody | Biolegend (111604) |
| PE anti-mouse Gr-1 antibody | Biolegend (108408) |
| FITC anti-human CD4 antibody | Biolegend (300506) |
| PE anti-human CD40L antibody | Biolegend (310806) |
| PE anti-human IL-4 antibody | Biolegend (500810) |
| PE anti-human IFNγ antibody | Biolegend (502509) |

**Supplementary Table 3**

Table 3.Correlations between CCT2 expression and clinicopathologic parameters in 93 breast cancer patients.


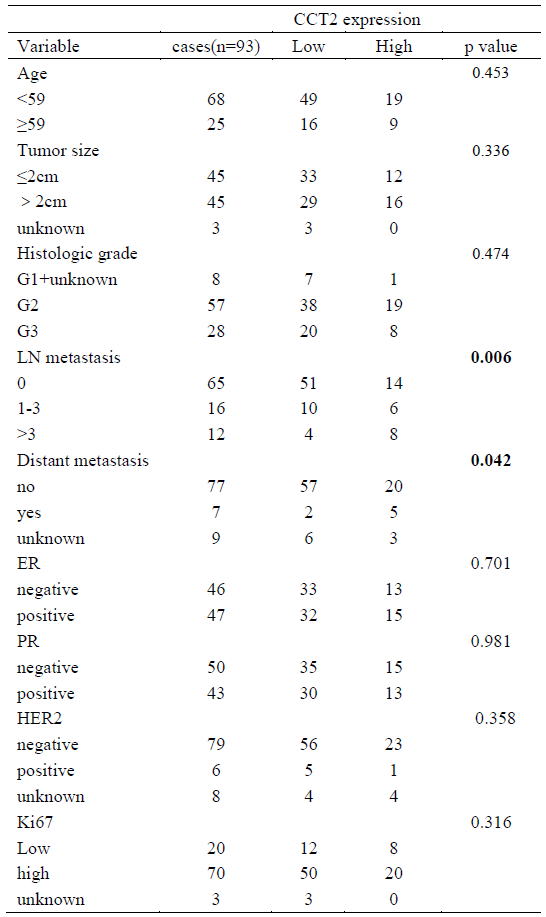


Abbreviation: LN=lymph nodes; ER=estrogen receptor; PR=progesterone receptor; HER2=human epidermal growth factor receptor-2; P value <0.05 marked in bond font to show statistically significant.

**Supplementary Table 4**

Table 4. Univariate and multivariate Cox regression analyses for OS of 93 breast cancer patients.

**
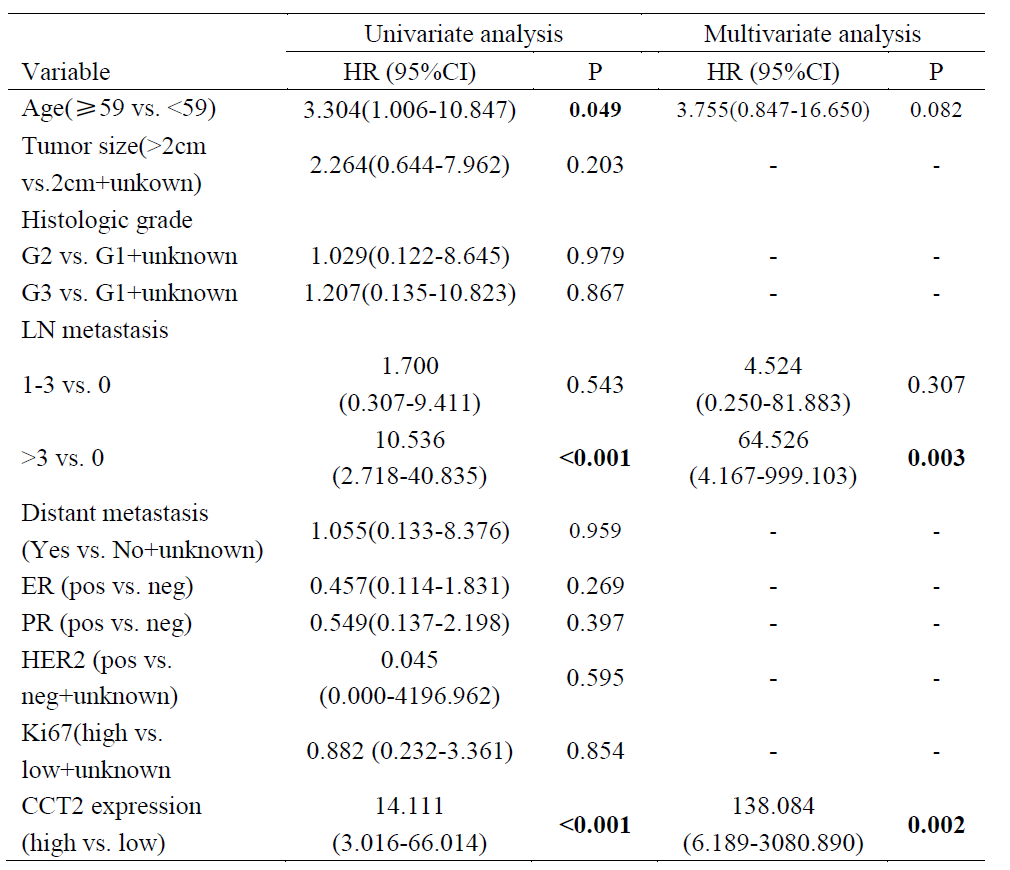
**

Abbreviation:95%CI= 95% confidence interval; HR= hazard ratio; LN=lymph nodes; ER=estrogen receptor; PR=progesterone receptor; HER2=human epidermal growth factor receptor-2; P value <0.05 marked in bond font to show statistically significant.

**Supplementary figure**

**Supplementary figure 1**

**
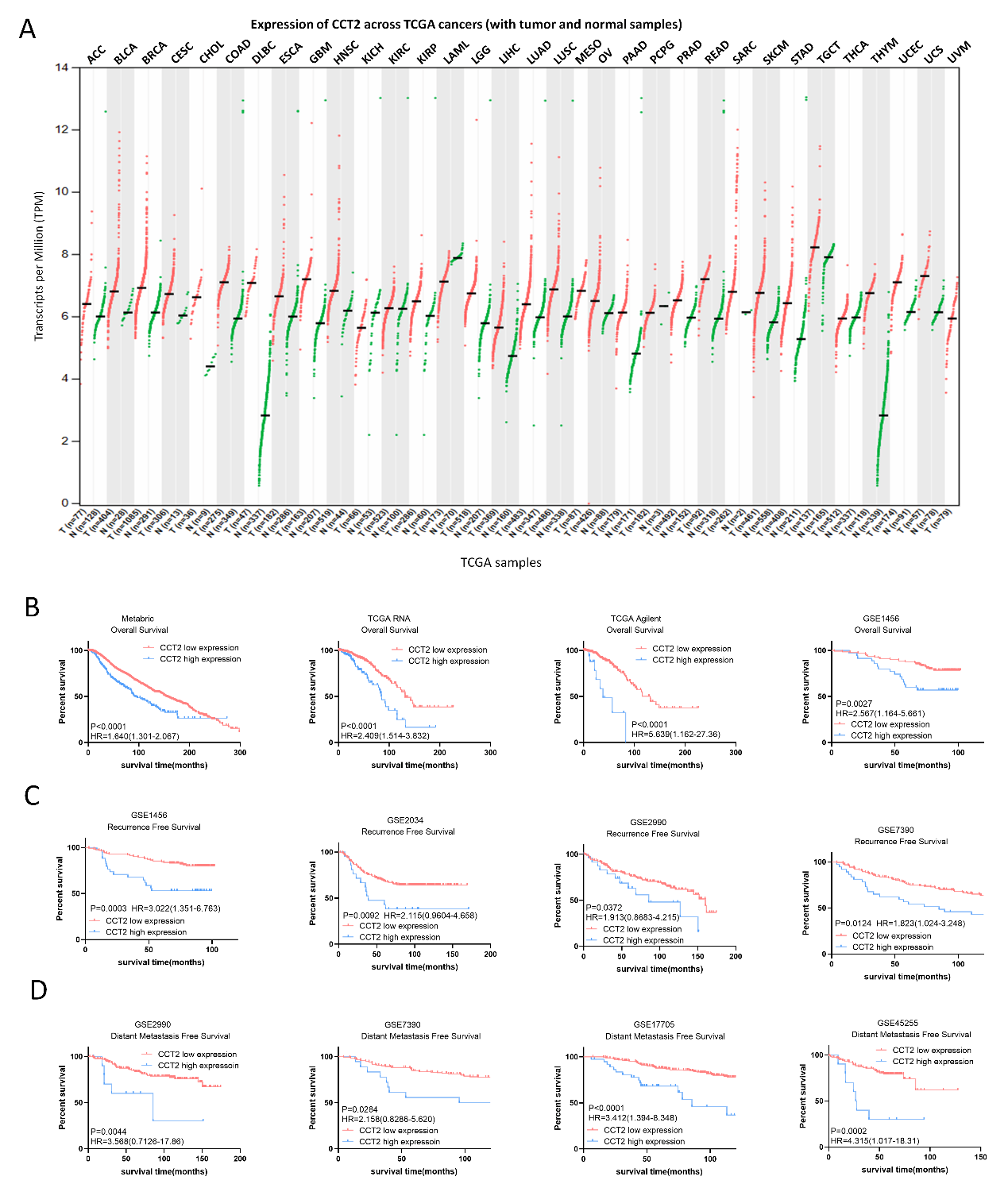
**

**Figure S1. The expression of CCT2 was upregulated in breast cancer tissues and associated with poor prognosis of breast cancer patients. A** The RNA expression of CCT2 was upregulated in various cancers according to TCGA database. **B** high expression of CCT2 was associated with poor OS of breast cancer patients. **C** high expression of CCT2 was associated with poor RFS of breast cancer patients. **D** high expression of CCT2 was associated with poor DMFS of breast cancer patients. (**P*<0.05, ***P*<0.01, ****P*<0.001).

**Supplementary figure 2**

**
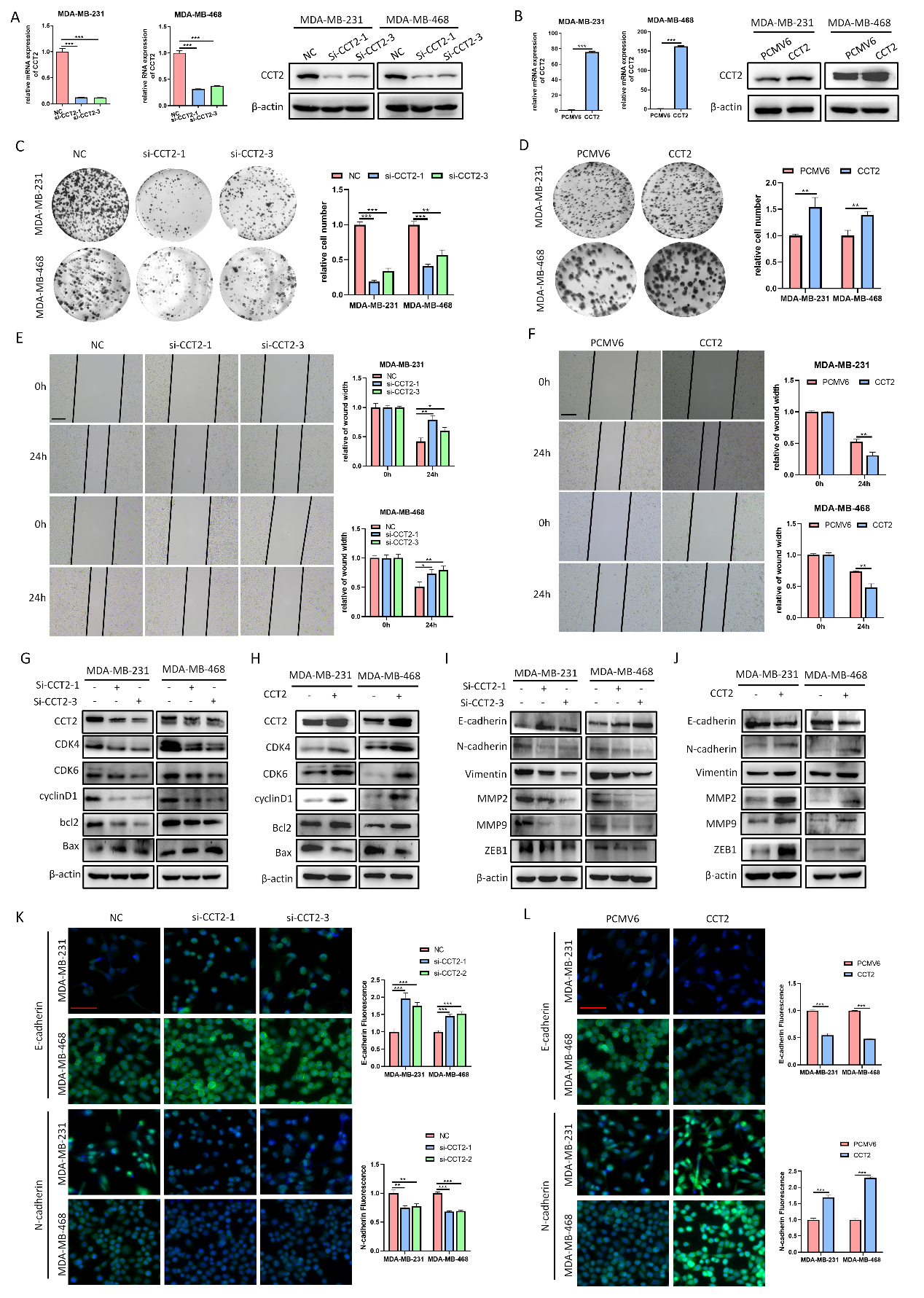
**

**Figure S2. CCT2 promoted breast cancer cells malignant progression in vitro. A** The interfering efficacies of si-CCT2 was measured by qPCR and western blot in MDA-MB-231 and MDA-MB-468. **B** CCT2 overexpression efficiency was confirmed by qPCR and western blot in MDA-MB-231 and MDA-MB-468. **C,D** Colony formation assays were performed to evaluate the effect of CCT2 on cell proliferative ability. **E,F** Wound healing assays was used to evaluated the effect of CCT2 on the migration ability of breast cancer cells. Scale bar =200μm. **G,H** Western blot was used to examine the expression of cell cycle-related and apoptotic related proteins after CCT2 knockdown or overexpression. **I,J** Western blot showed the effect of CCT2 on the expression of EMT-related proteins. **K,L** immunofluorescence assay of E-cadherin and N-cadherin in MDA-MB-231 and MDA-MB-468 after CCT2 knockdown or overexpression. Scale bar =50μm. (**P*<0.05, ***P*<0.01, ****P*<0.001).

**Supplementary figure 3**

**
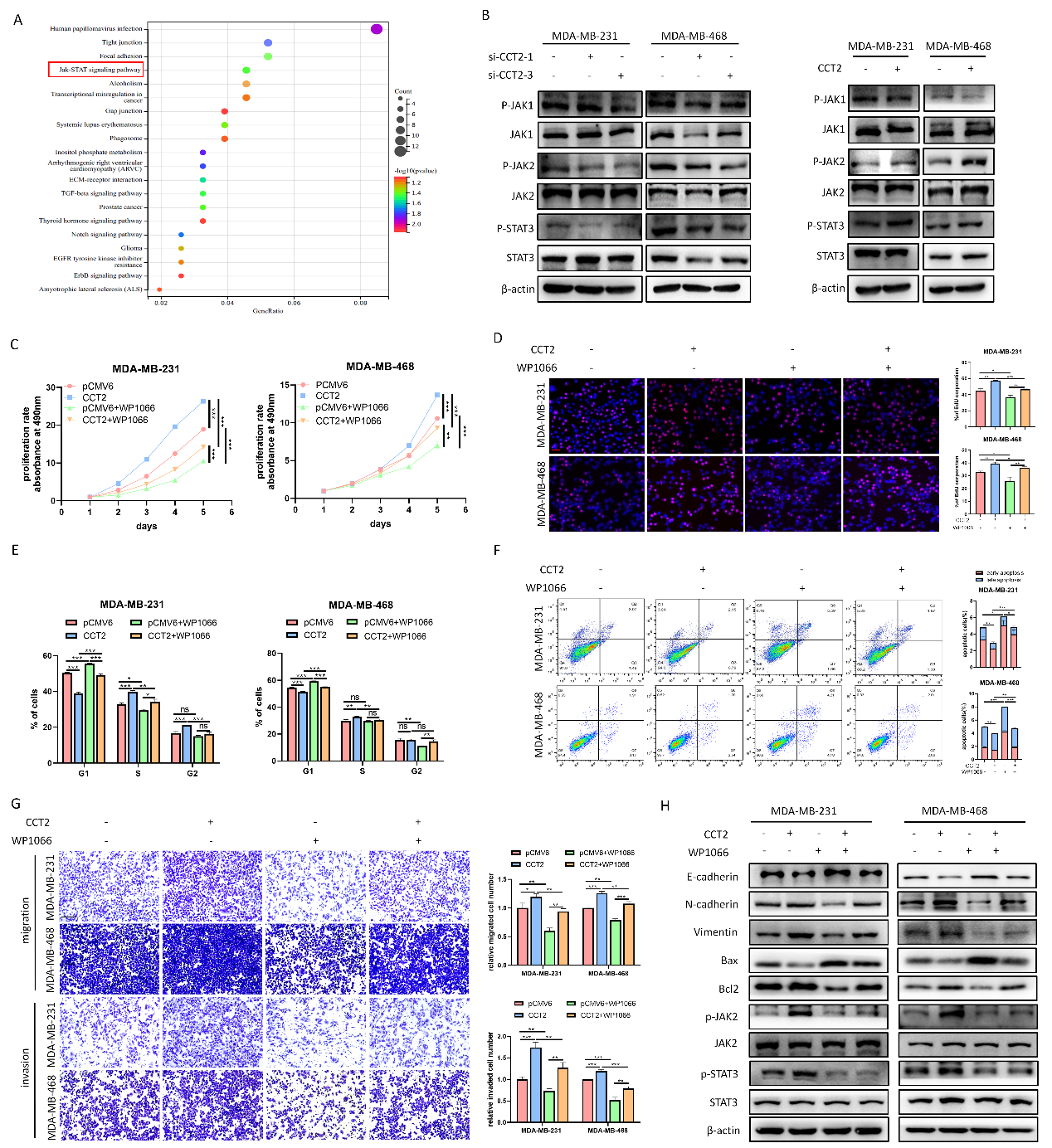
**

**Figure S3.** **CCT2 activated the JAK2/STAT3 signaling pathway to promote breast cancer cells malignant progression. A** Scatter plot of top 20 KEGG pathways enrichment of DEGs after CCT2 knockdown. Rich factor is the ratio of the DEG number to the background number in a certain pathway. The size of the dots represents the number of genes, and the color of the dots represents the range of the q-value. **B** Total and phosphorylated JAK1, JAK2 and STAT3 protein expression levels were analyzed in CCT2 knockdown and overexpression in breast cancer cells by western blot. The proliferative potential of CCT2-overexpressing breast cancer cells treated with WP1066 or DMSO were analyzed by the MTT(**C**) and EdU(**D**) assay. Scale bar =100μm. **E** Cell cycle distribution in CCT2-overexpressing breast cancer cells treated with WP1066 or DMSO were presented by flow cytometry. **F** Flow cytometry apoptosis analysis of CCT2-overexpressing breast cancer cells treated with WP1066 or DMSO. **G** The migratory and invasive potential of CCT2-overexpressing breast cancer cells treated with WP1066 or DMSO were analyzed by the transwell cell migration and invasion assay. Scale bar =200μm. **H** The expression levels of E-cadherin, N-cadherin, vimentin, Bax, Bcl2, JAK2, p-JAK2, STAT3, and p-STAT3 protein in CCT2-overexpressing breast cancer cells treated with WP1066 or DMSO were detected by western blot.

**Supplementary figure 4**

**
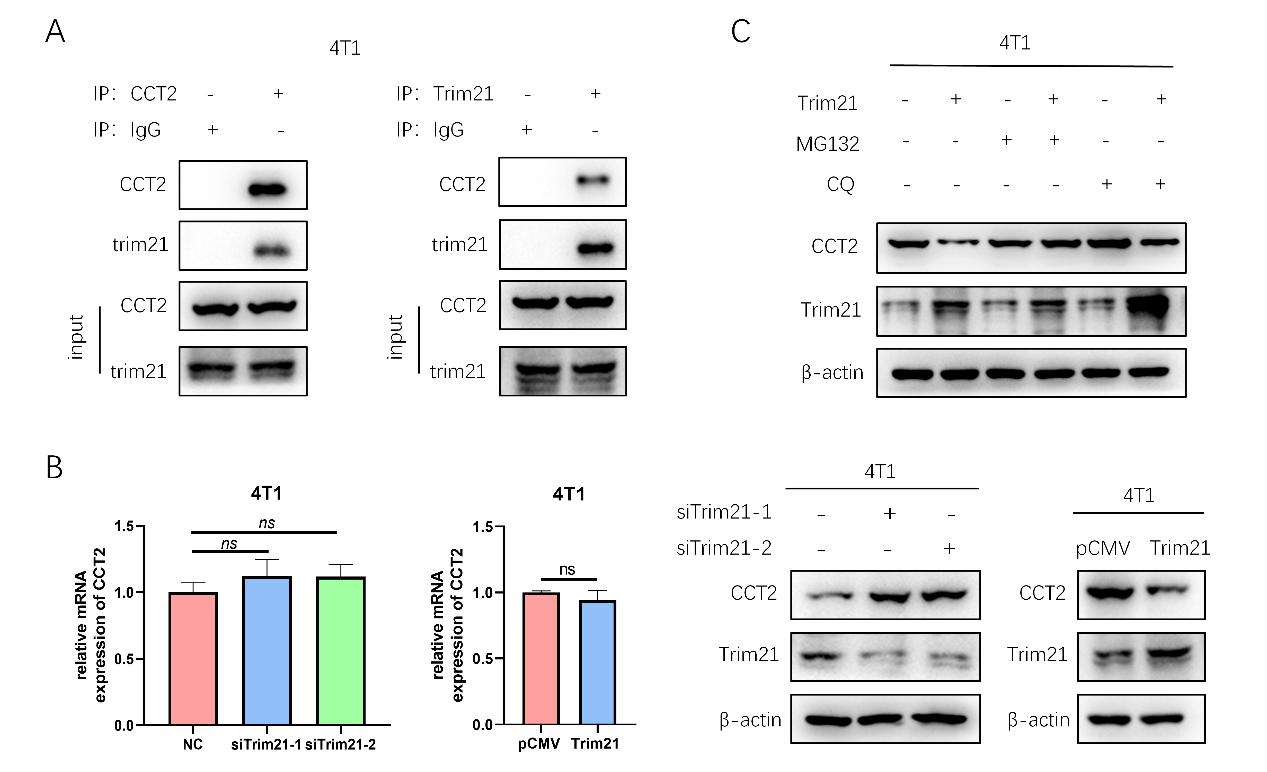
**

**Figure S4.** **Trim21 mediates the degradation of CCT2 in 4T1 cells. A** validation of biochemical interaction between CCT2 and Trim21 in 4T1 cells by coimmunoprecipitation of endogenous CCT2 and Trim21. **B** qPCR and immunoblotting were performed to evaluate the expression of CCT2 in Trim21-silenced or Trim21- overexpressing 4T1 cells. **C** Following transfection with the CCT2 and Trim21 plasmids and treatment with CQ, and MG132 for 4h, 4T1 cell lysates were subjected to western blotting. (**P*<0.05, ***P*<0.01, ****P*<0.001).

**Supplementary figure 5**

**
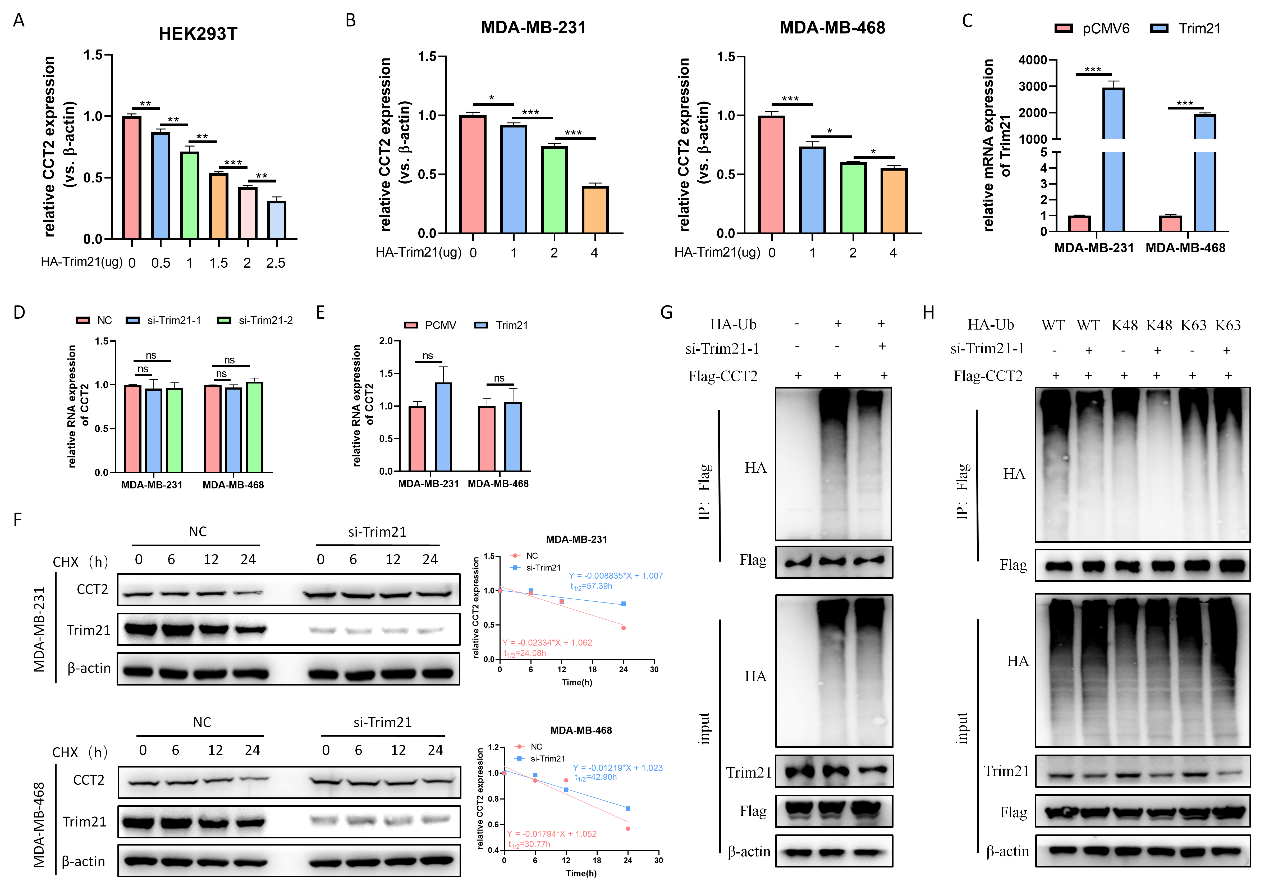
**

**Figure S5. Knockdown of Trim21 inhibited the ubiquitination and degradation of CCT2. A,B** the statistical analysis showed overexpression of Trim21 suppressed the protein levels of CCT2 in a dose dependent manner. C The overexpressing efficacies of Trim21 was measured by qPCR in MDA-MB-231 and MDA-MB-468. qPCR was performed to evaluate the expression of CCT2 in Trim21-silenced (**D**) or Trim21-overexpressing (**E**) breast cancer cells. **F** MDA-MB-231 and MDA-MB-468 cells were transfected with si-Trim21, followed by cycloheximide (CHX) treatment for indicated duration. Immunoblotting was then performed using lysates prepared from these cells, with the ImageJ software being used to quantify CCT2 expression and β-actin being used for normalization. **G** Anti-flag was used to immunoprecipitated lysates prepared from 293T cells following transient HA-Ub, Flag-CCT2 and si-Trim21 co-transfection, after which anti-HA was used for immunoblotting. **H** immunoprecipitation analyses were performed for lysates from 293T cells following the transient co-transfection of Flag-CCT2, si-Trim21, and total-Ub or K48-Ub or K63-Ub mutant. (**P*<0.05, ***P*<0.01, ****P*<0.001).

**Supplementary figure 6**

**
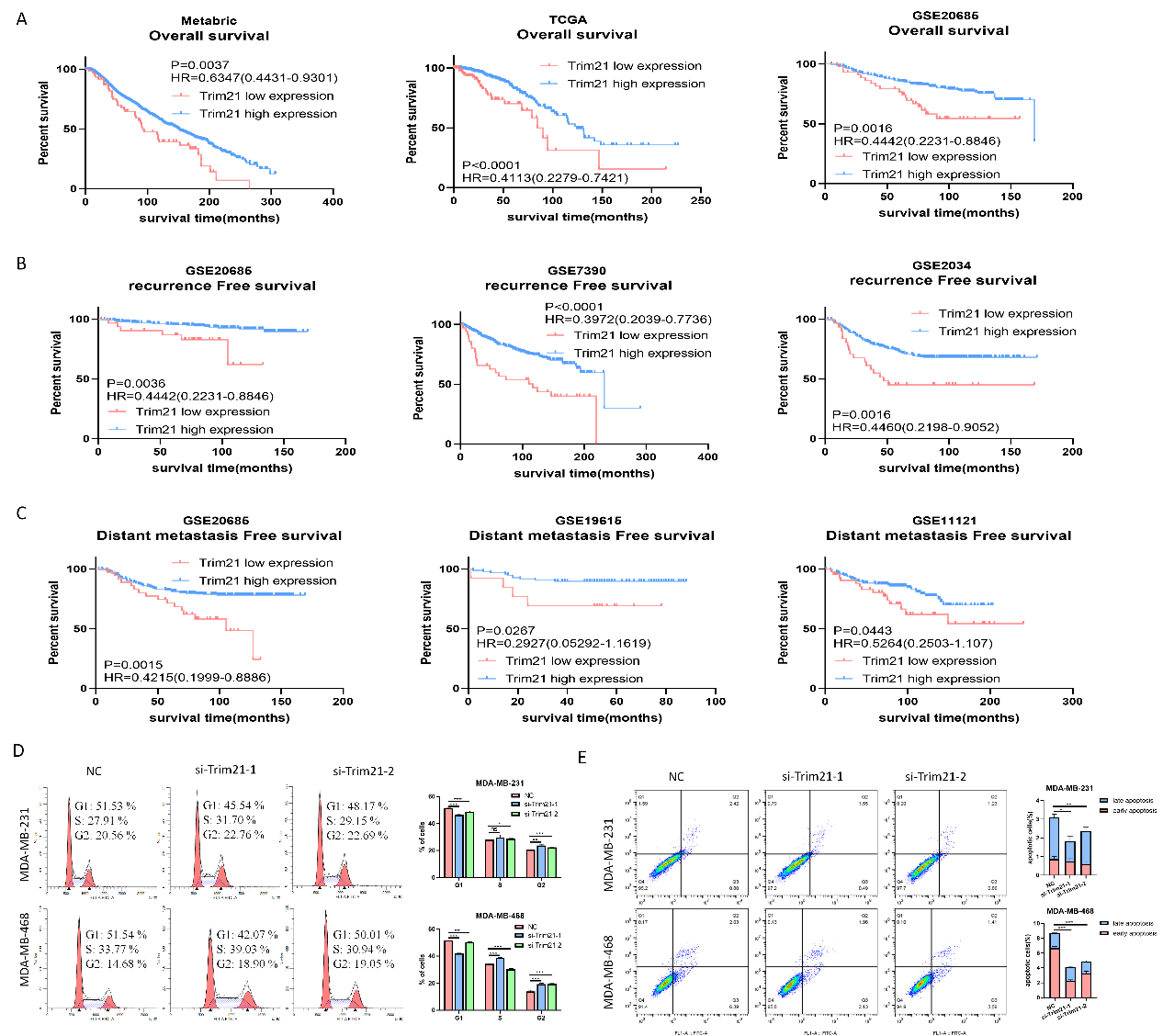
**

**Figure S6. Trim21 was correlated with better prognosis of breast cancer patients and Trim21 knockdown promoted proliferation and inhibited apoptosis of breast cancer cells. A** higher expression of Trim21 was associated with better OS of breast cancer patients. **B** higher expression of Trim21 was associated with better RFS of breast cancer patients. **C** higher expression of Trim21 was associated with better DMFS of breast cancer patients. **D** Cell cycle distribution in Trim21 knockdown breast cancer cells were presented by flow cytometry. **E** Flow cytometry apoptosis analysis of Trim21 knockdown breast cancer cells. (**P*<0.05, ***P*<0.01, ****P*<0.001).

**Supplementary figure 7**

**
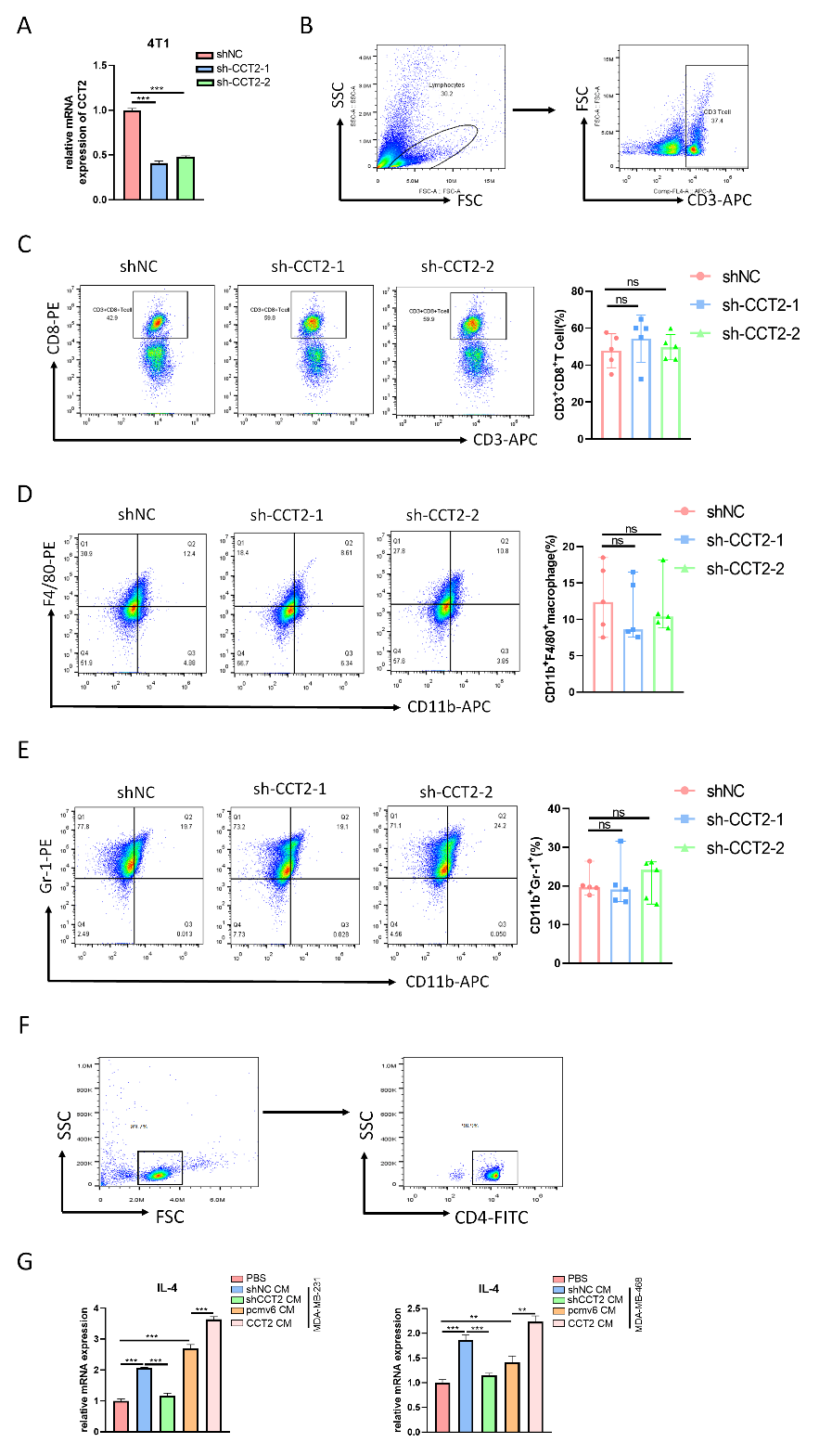
**

**Figure S7. CCT2 had no effect on tumor infiltrating immune cells in vivo and inhibited the anti-inflammatory cytokine production in CD4^+^T cells. A** The knockdown efficiency of CCT2 in 4T1 was analyzed by qPCR. B the specific steps of flow cytometry for sorting CD3^+^T cells. Flow cytometry analysis of CD3^+^CD8^+^T cells (**C**), CD11b^+^F4/80^+^ macrophages (**D**), and CD11b^+^Gr-1^+^myeloid-derived suppressor cells (**E**) from 4T1 tumors harvested from mice in three different groups (n=5). **F** The purity of CD4^+^T cells that were isolated from PBMC of human donors was determined by flow cytometry. **G** the levels of mRNA expression of IL-4 in CD4^+^T cells from each group were determined by qPCR. (**P*<0.05, ***P*<0.01, ****P*<0.001).

**Supplementary figure 8**

**
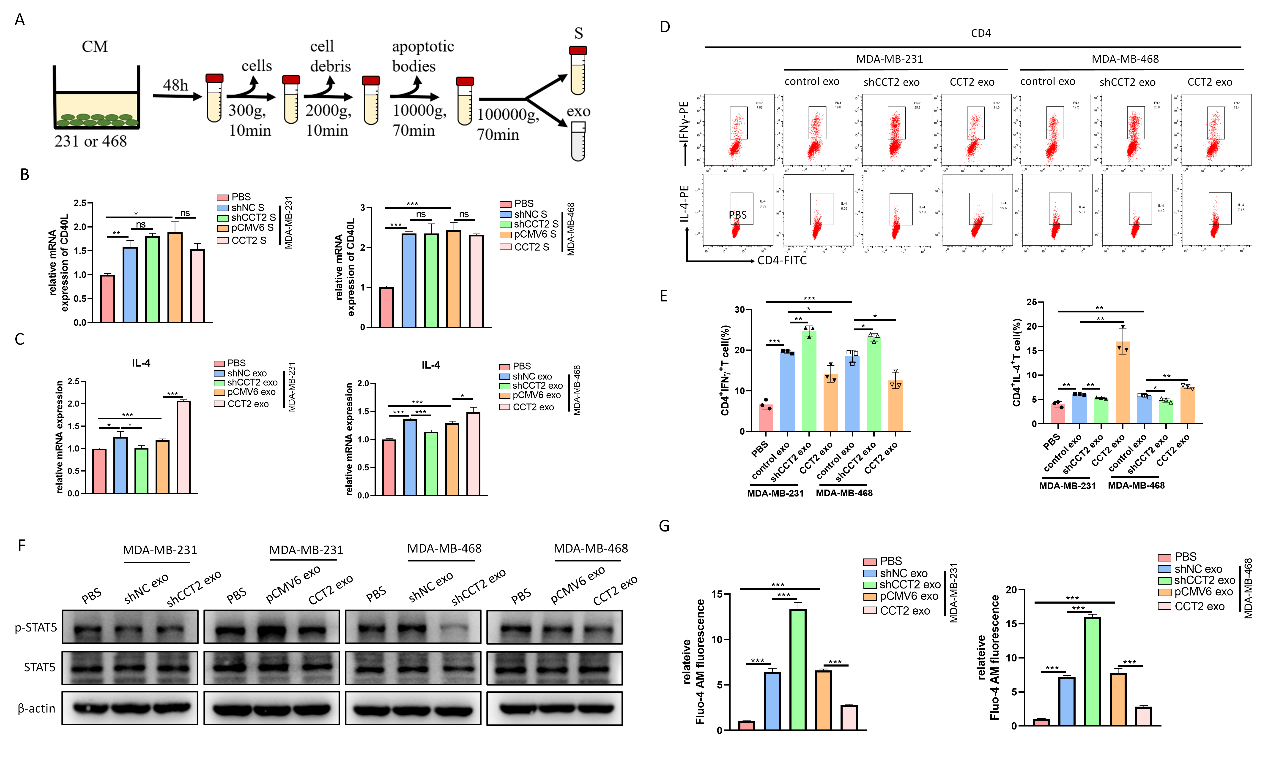
**

**Figure S8. Different breast cancer cell supernatants had no significant changes on the activation of CD4^+^T cells. A** The methodological procedure for obtaining breast cancer conditioned medium (CM), breast cancer supernatant (S), and breast cancer exosomes (exo) from different conditioned breast cancer for 48h. **B** qPCR analysis were used to detect CD40L expression on CD4^+^T cells under different conditioned breast cancer supernatant: PBS, shCCT2 or shNC breast cancer cell supernatant, and pCMV6 or CCT2 overexpressing breast cancer cell supernatant. **C** the levels of mRNA expression of IL-4 in CD4^+^T cells from each exos group were determined by qPCR. **D,E** flow cytometry analysis was used to detect cytokine secretion on CD4^+^T cells under different conditioned breast cancer exos. **F** the activation of STAT5 (p-STAT5) in CD4^+^T cells from different exos group were detected by western blot. **G** quantitative analysis of fluo-4 AM fluorescence assay to measure the level of Ca2^+^ influx in CD4+T cells from each group. (**P*<0.05, ***P*<0.01, ****P*<0.001).

**Supplementary figure 9**
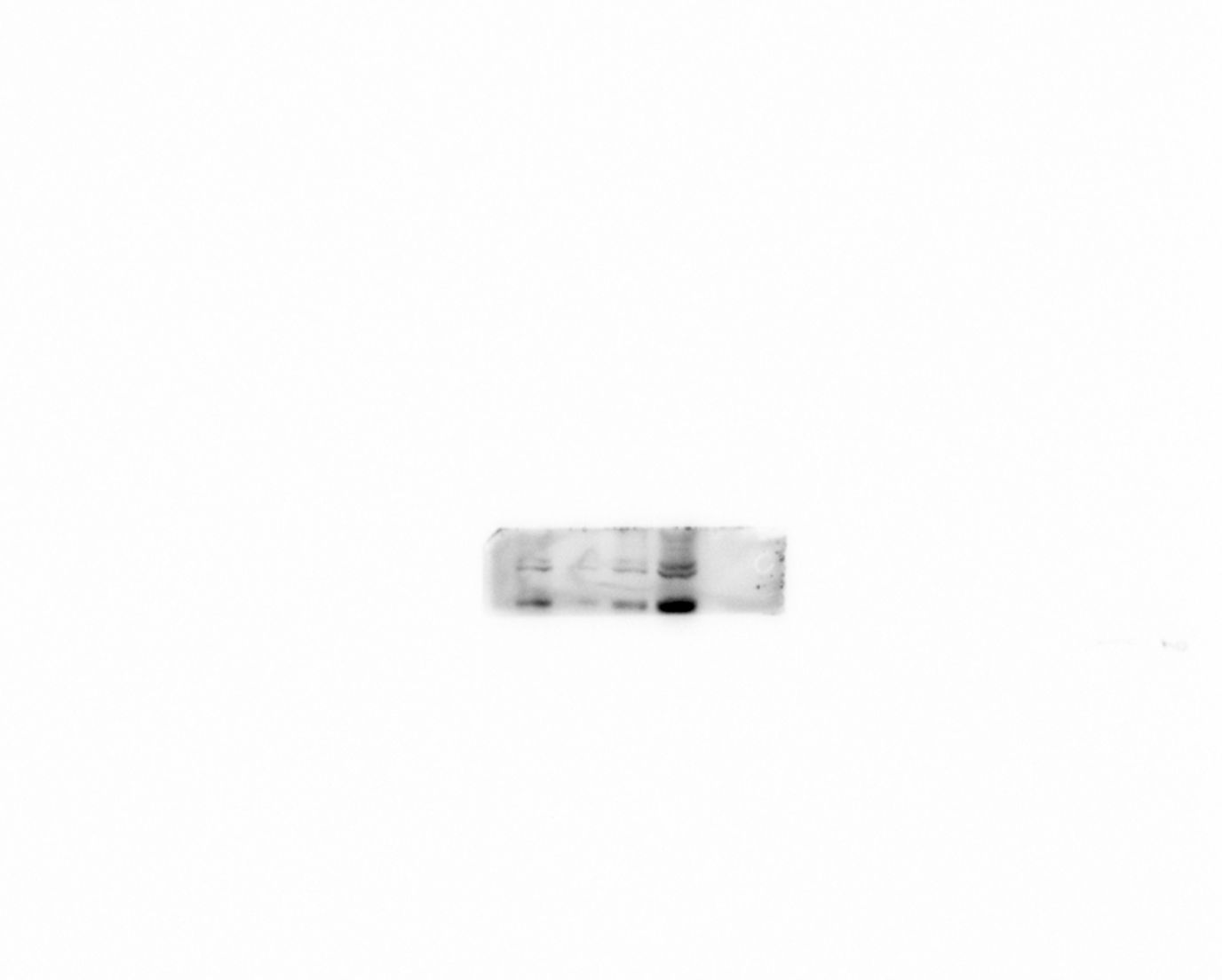


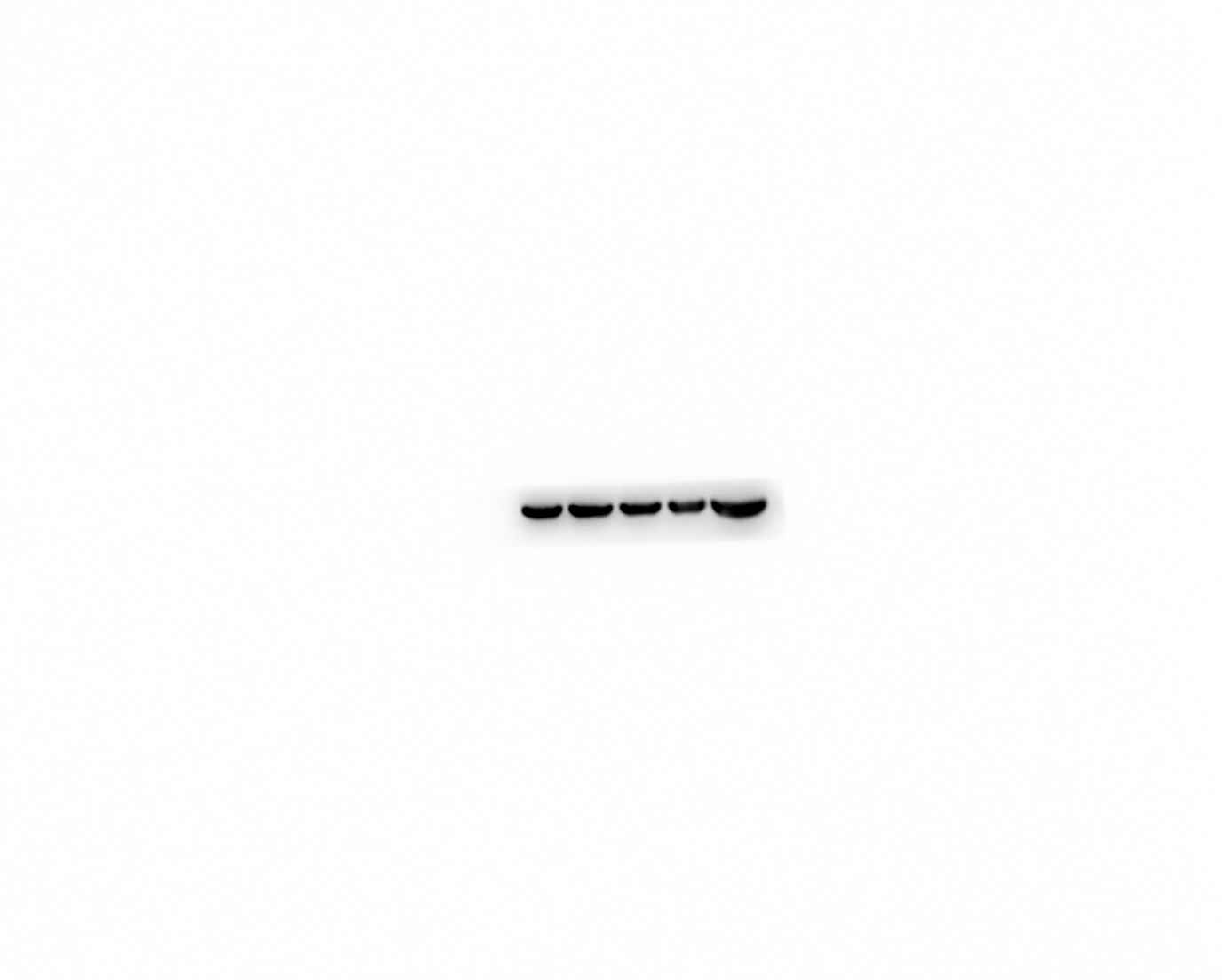


β-actin


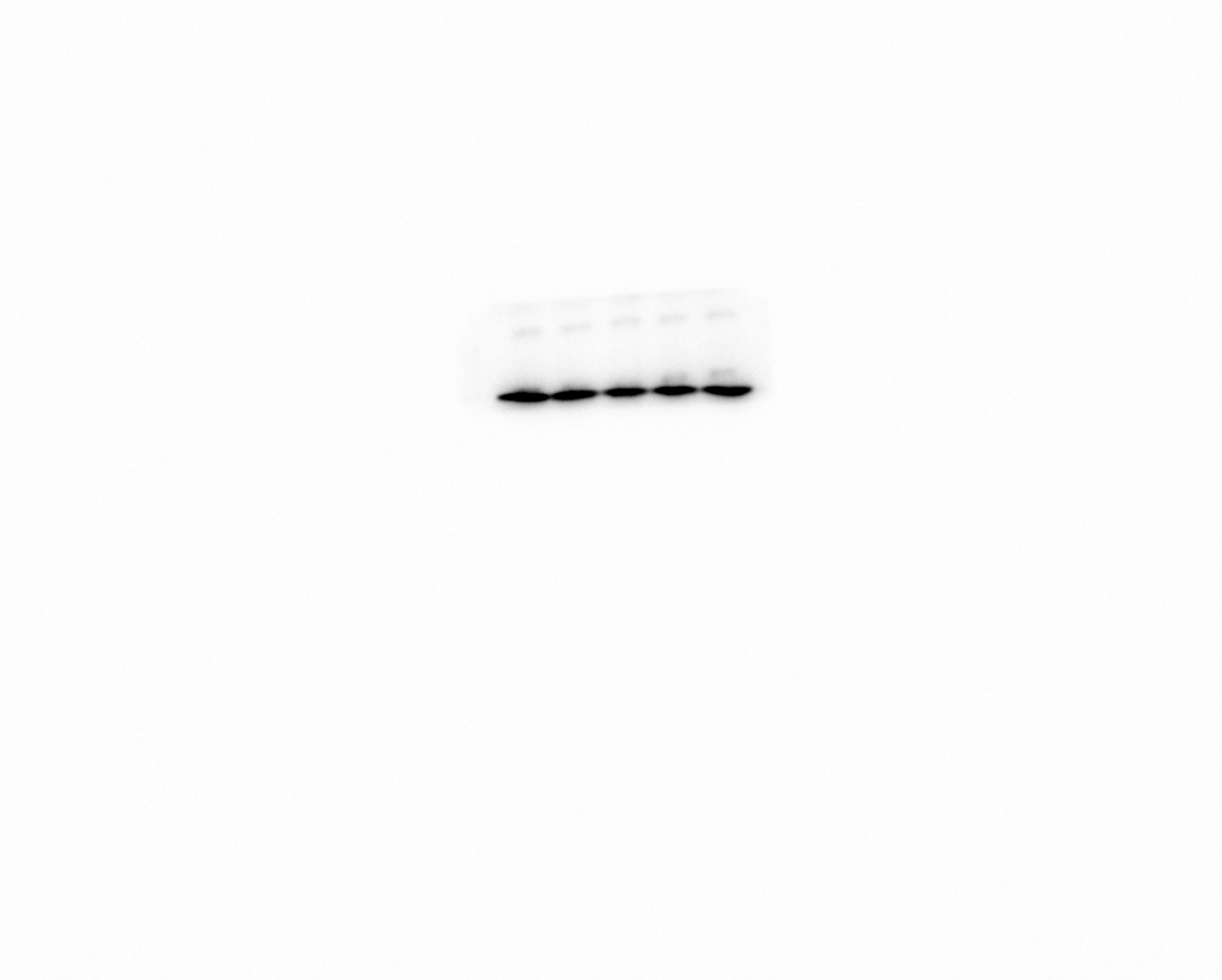


H3

H3


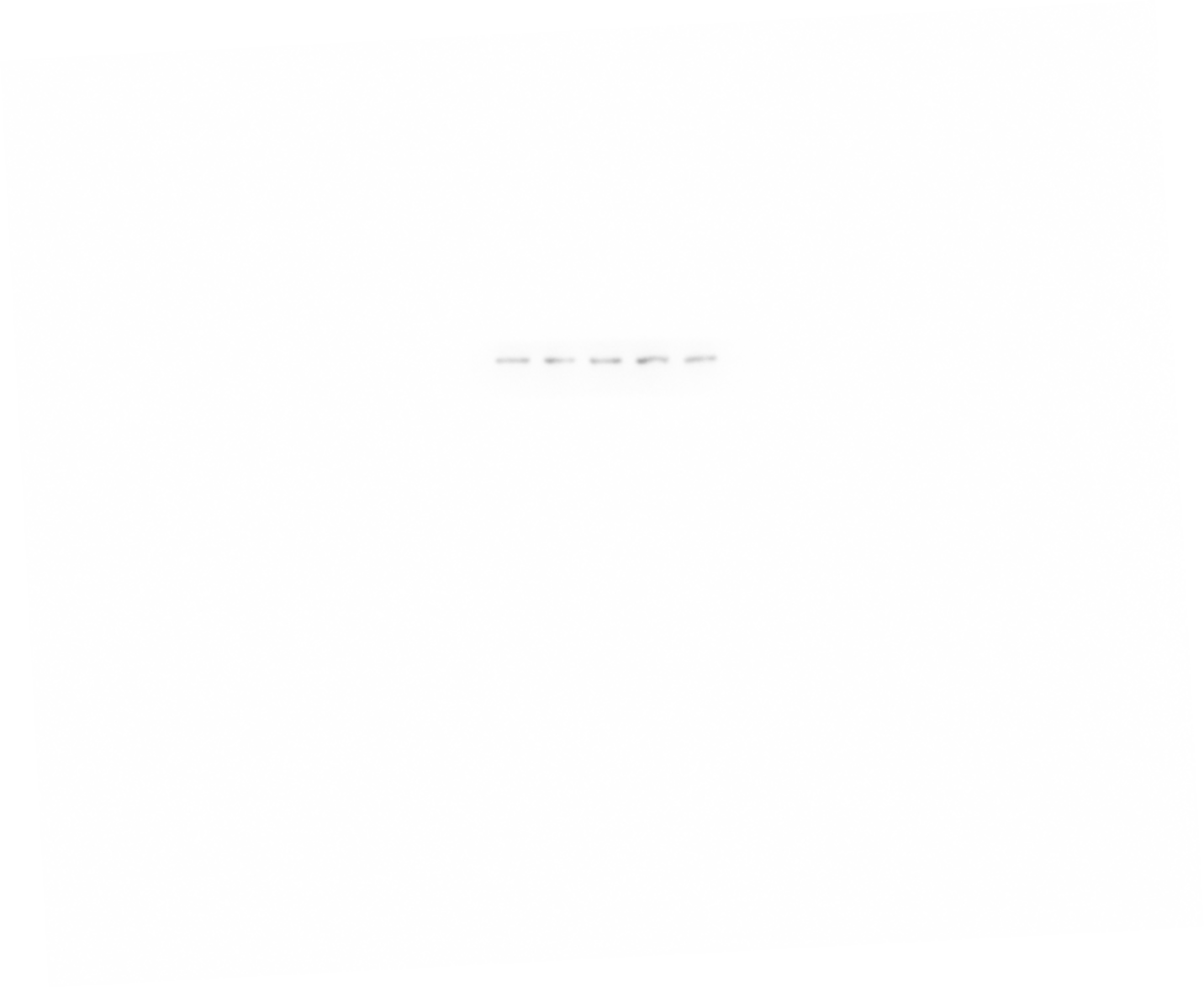


β-actin

NFAT1


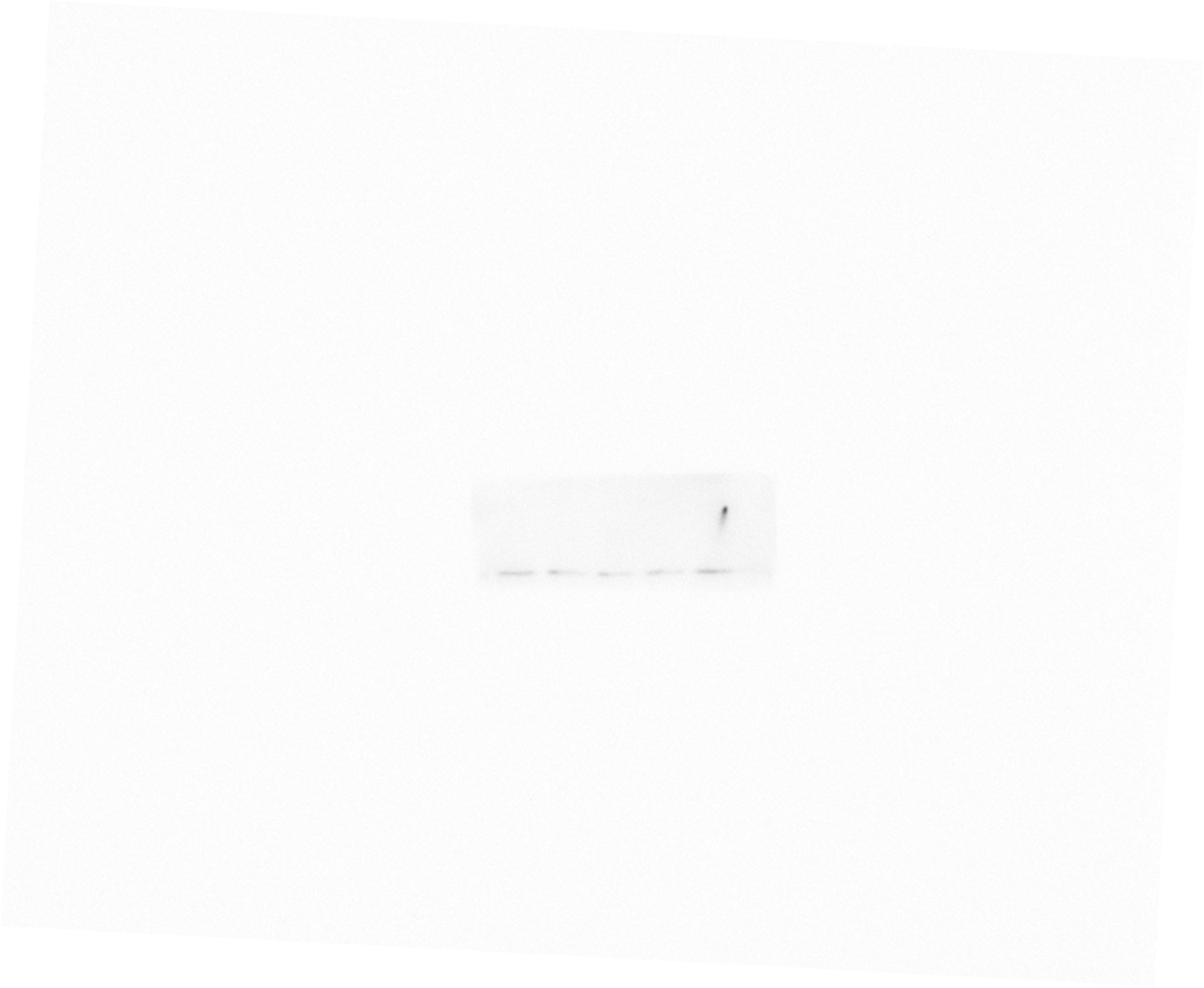

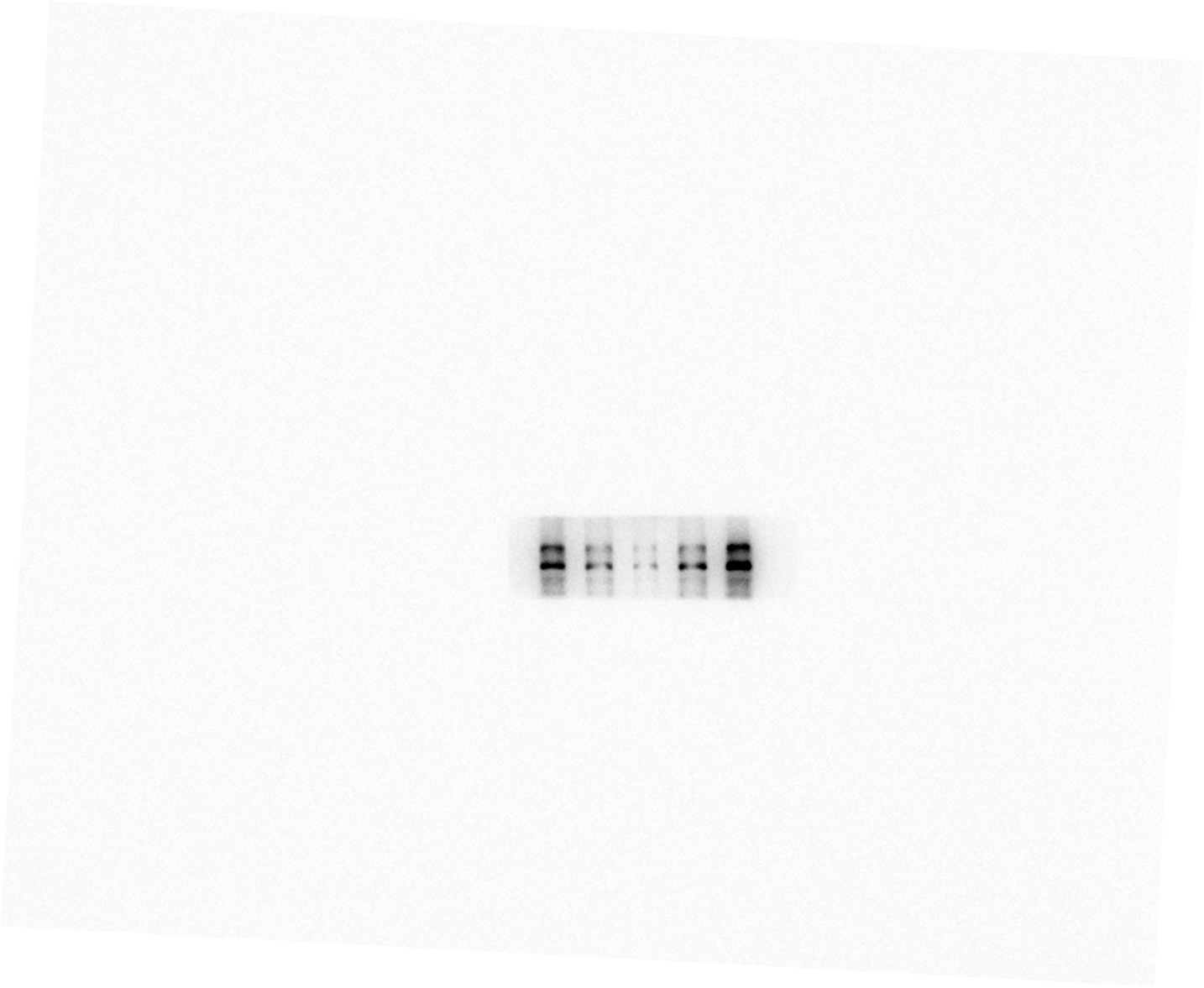

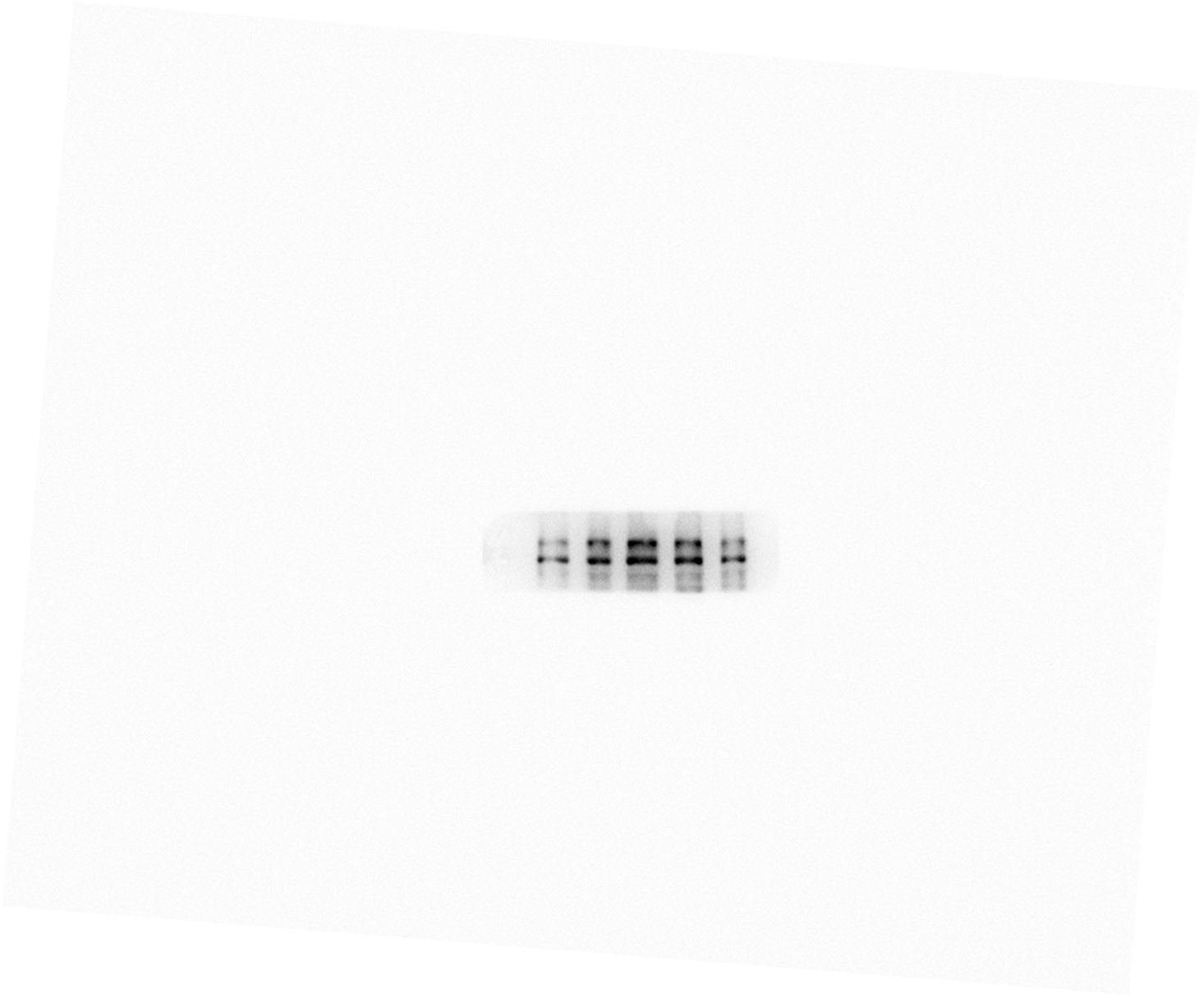


NFAT1

PBS 1 2 3 4

4T1

1.shNC exo 2.shCCT2 exo

3.pCMV6 exo 4.CCT2 exo

B

C

D

E


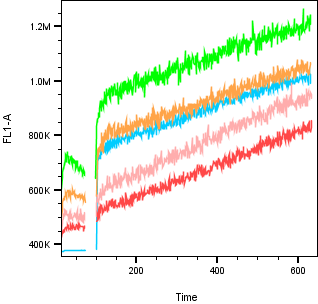


PBS

shNC exo

shCCT2 exo

pCMV6 exo

CCT2 exo

4T1

Fluorescent intensity

Fluo-4 AM

CCT2

**
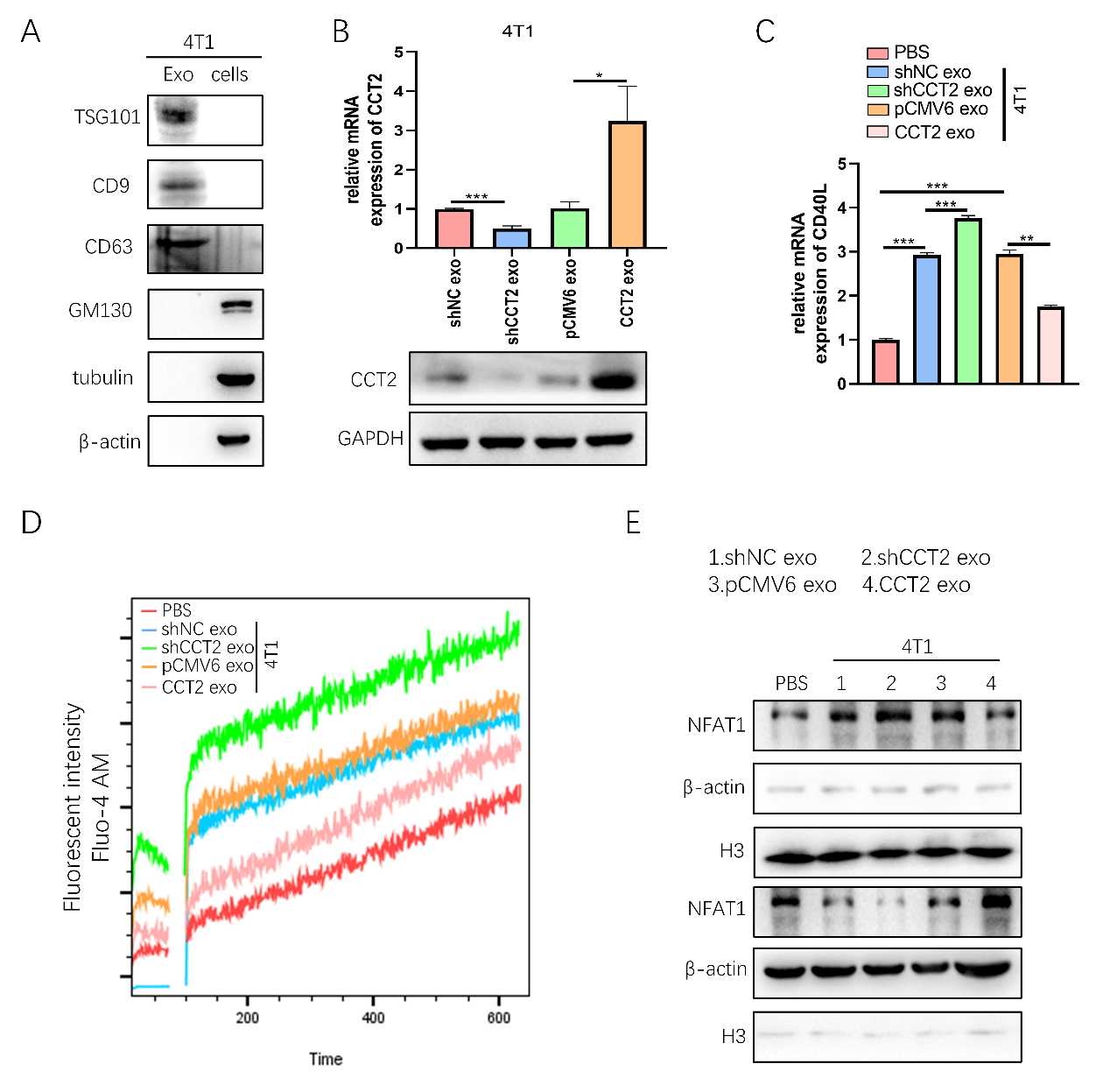
**

**Figure S9 Mouse exosomal CCT2 suppresses CD4^+^T cells activation. A** Western blot analysis of exosomal markers in the exos isolated from 4T1 cells. **B** CCT2 expression of mouse CD4^+^T cells treated with exos released by indicated cells were detected by qPCR and western blot. **C** qPCR analysis was used to detect CD40L expression on mouse CD4^+^T cells under different conditioned 4T1 exos: PBS, shCCT2 or shNC 4T1 cell exos, and pCMV6 or CCT2 overexpressing 4T1 cell exos. **D** Representative curves of Ca^2+^ influx dynamics in mouse CD4^+^T cells from each group were depicted using Fluo-4AM fluorescence. **E** The expression levels of NFAT1 in the cytosolic and nuclear fractions of mouse CD4^+^T cells from each group were detected by western blot.
