# Supplementary material for: Trim21-mediated CCT2 ubiquitination suppresses malignant progression and promotes CD4+T cell activation in breast cancer
Source: Cell Death Dis. 2024 Jul 30;15(7):542. doi: 10.1038/s41419-024-06944-8 (PMC11289294; doi:10.1038/s41419-024-06944-8)

Fig1E

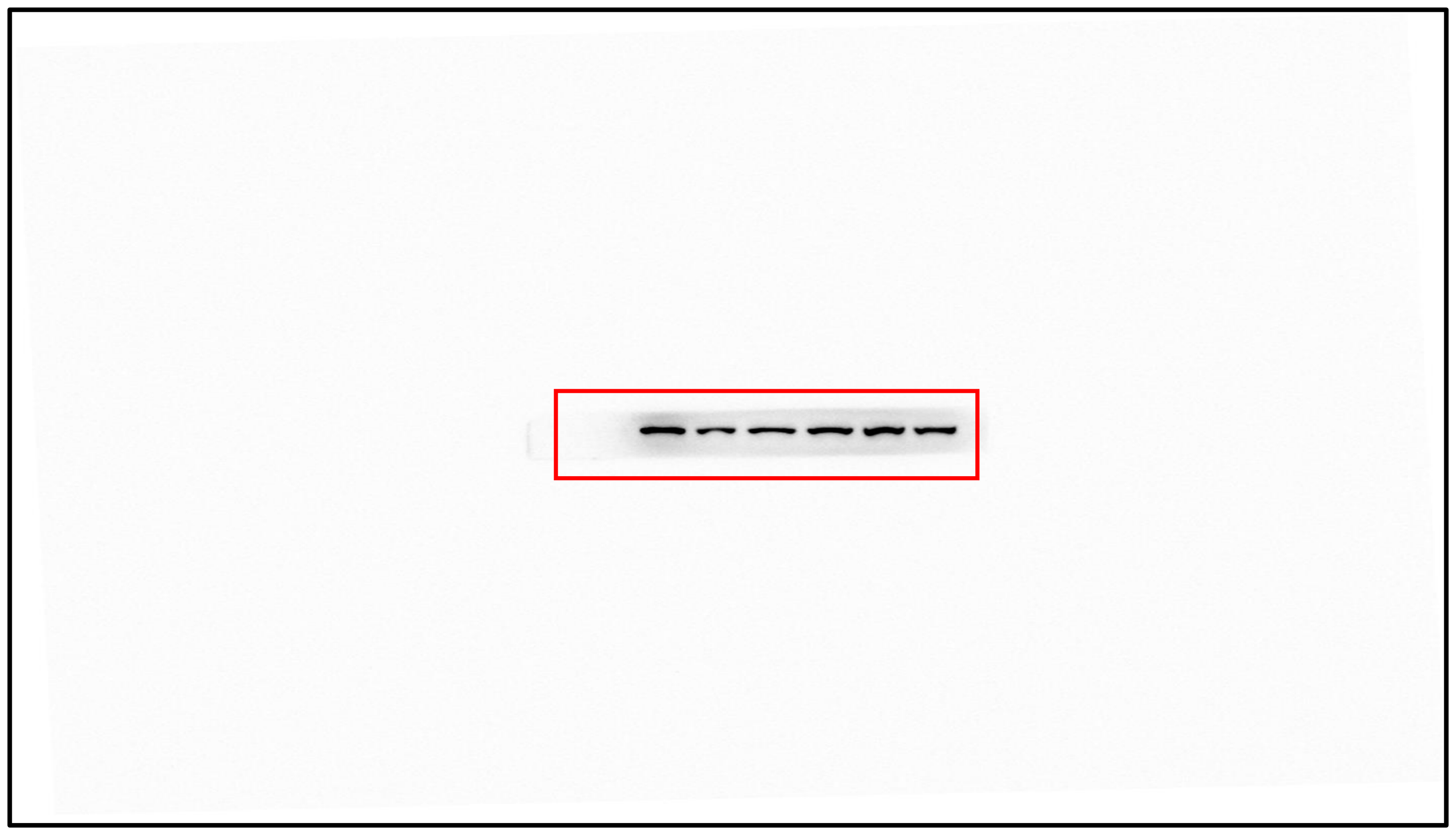

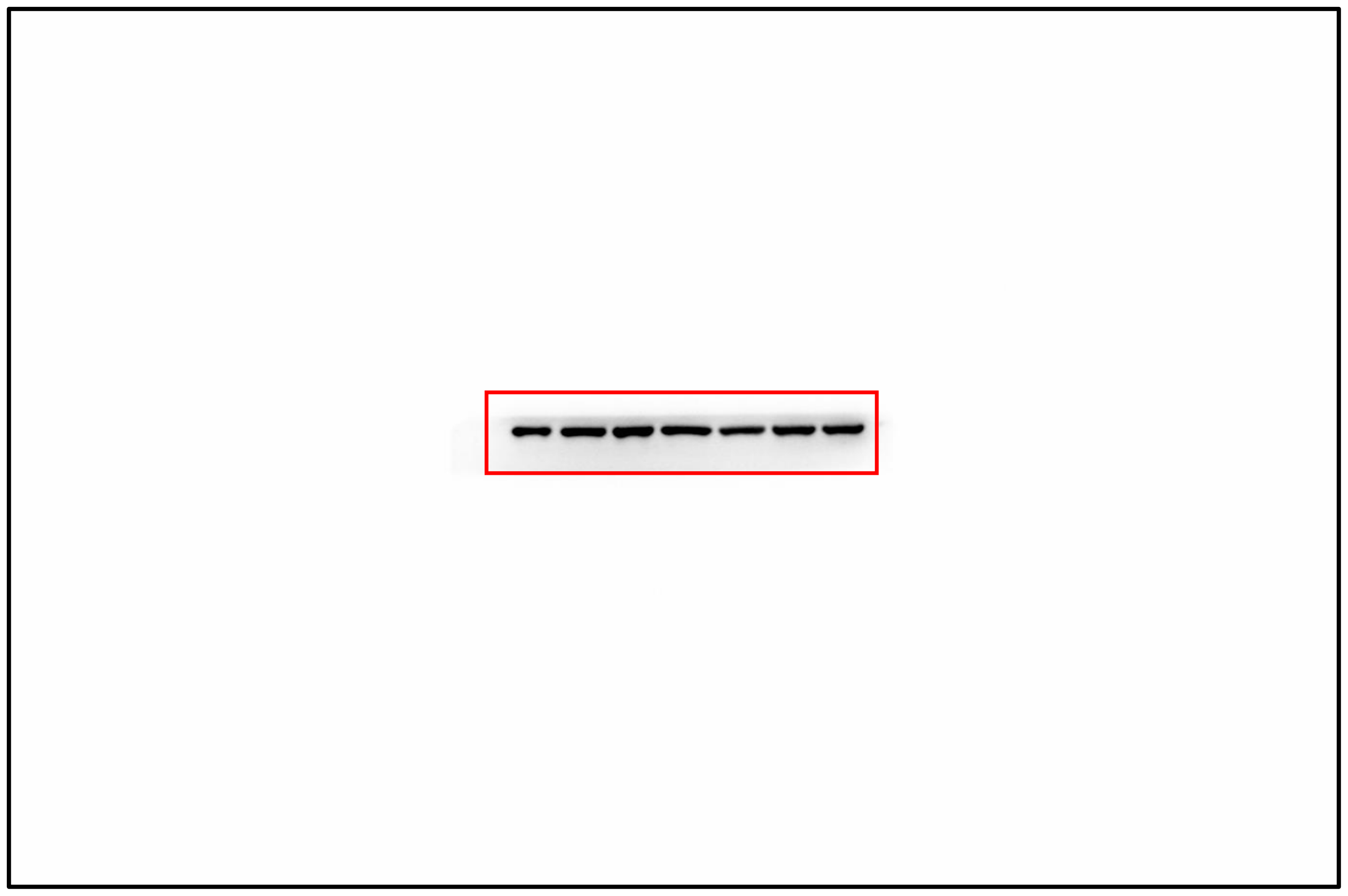

Fig3B

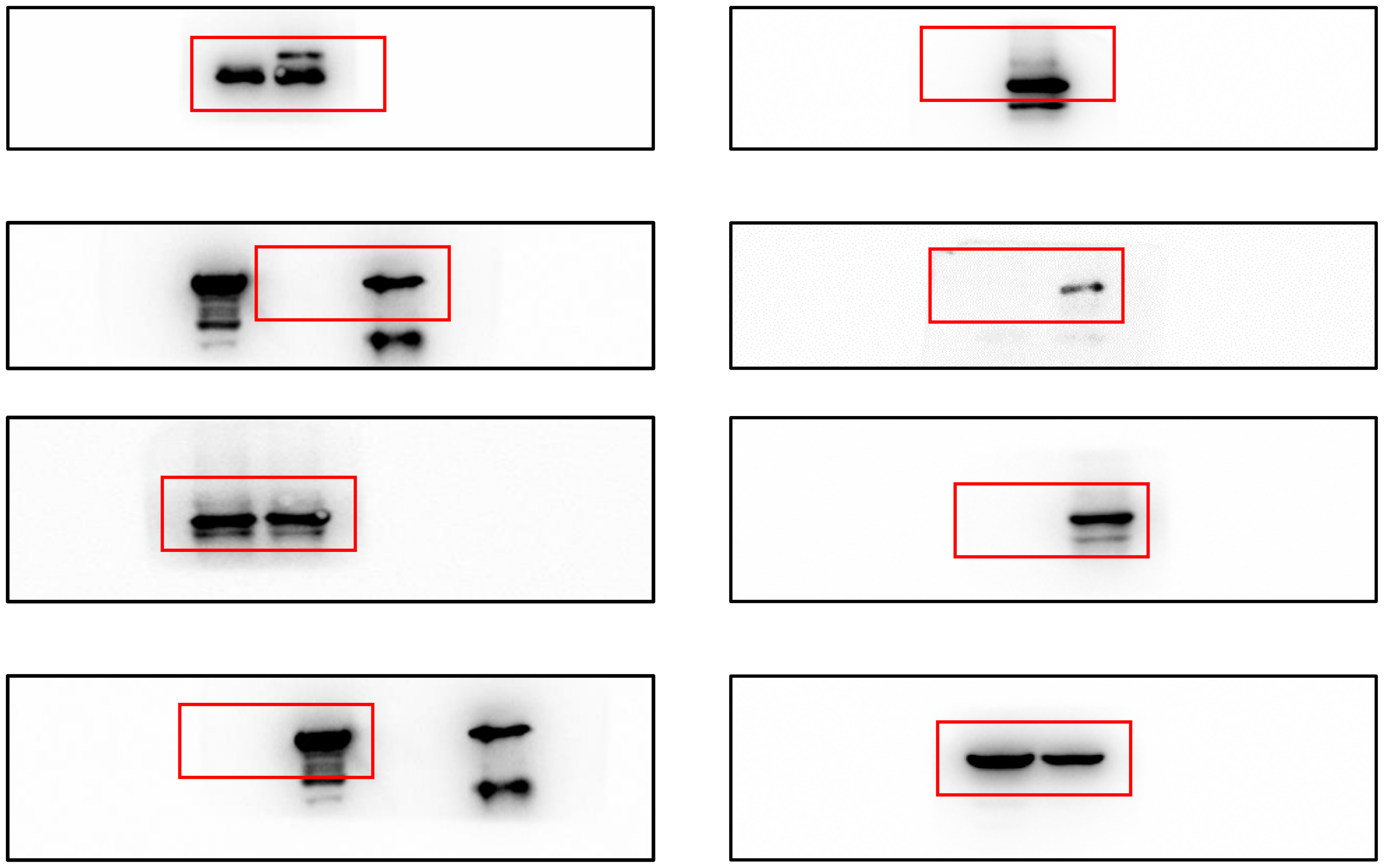

Fig3C

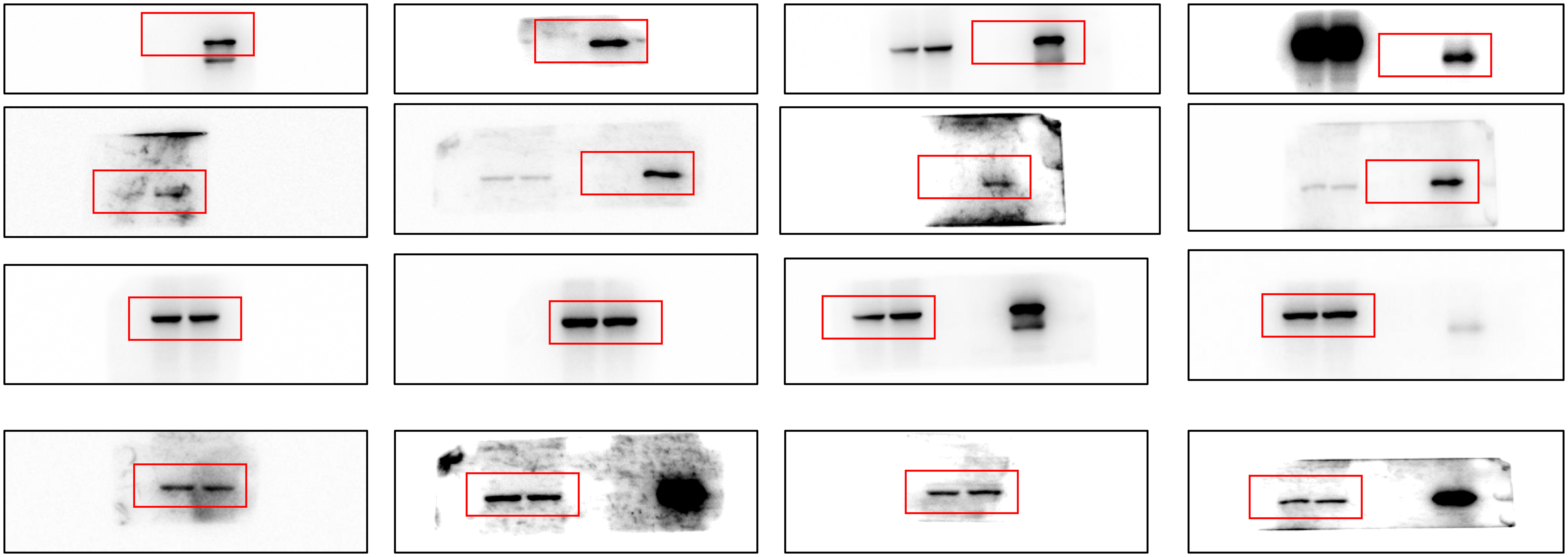

Fig3F

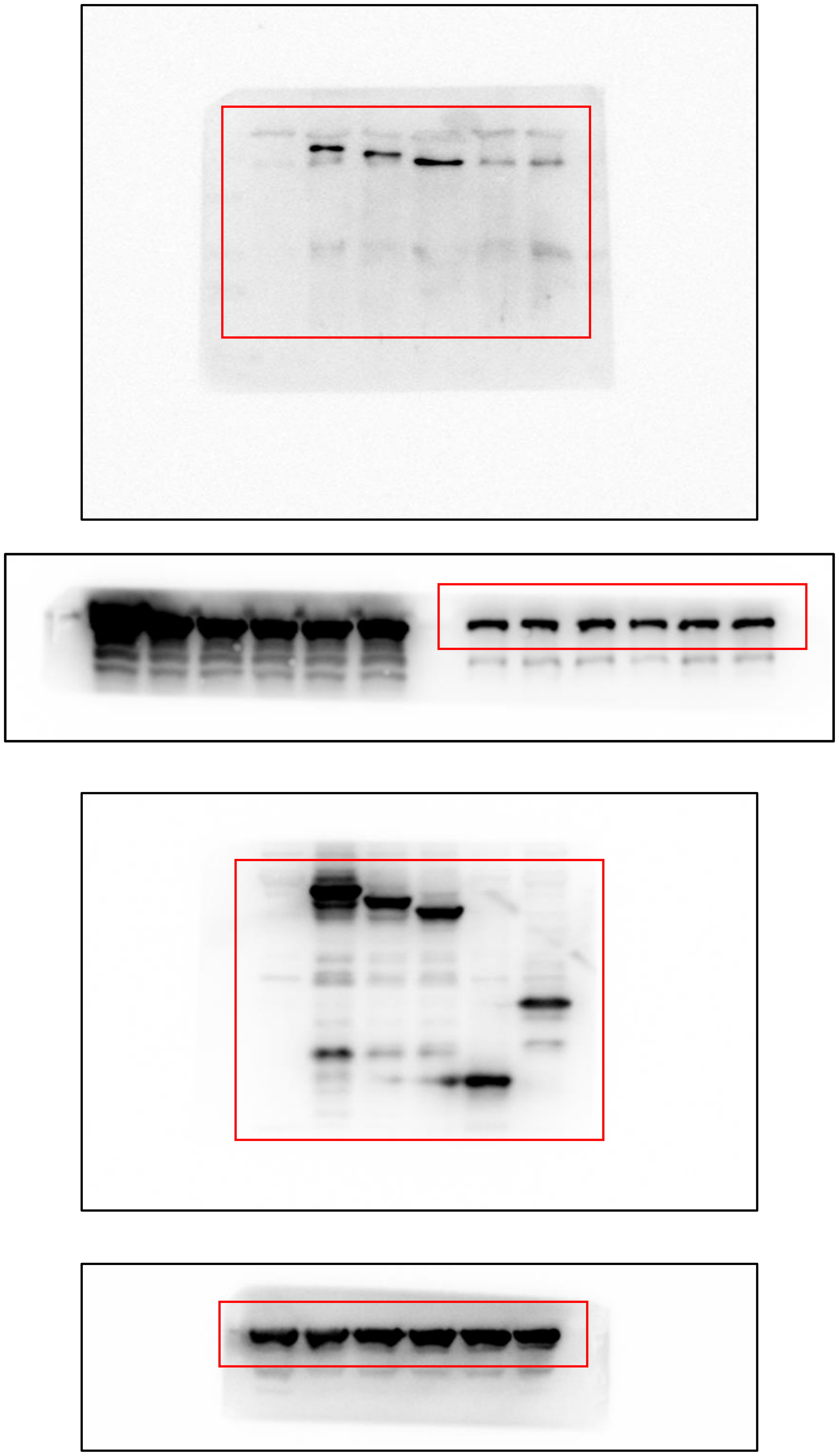

Fig3H

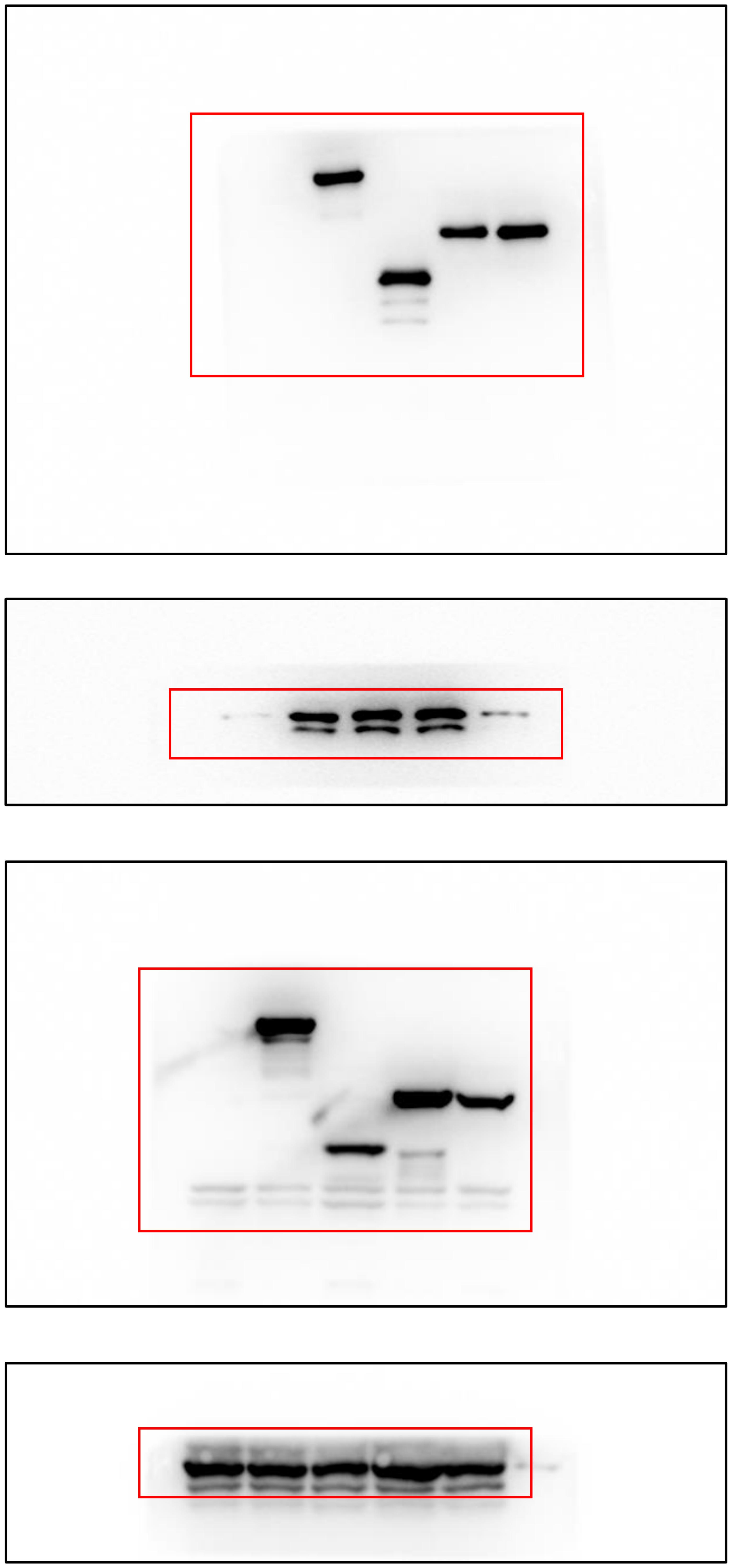

Fig4A

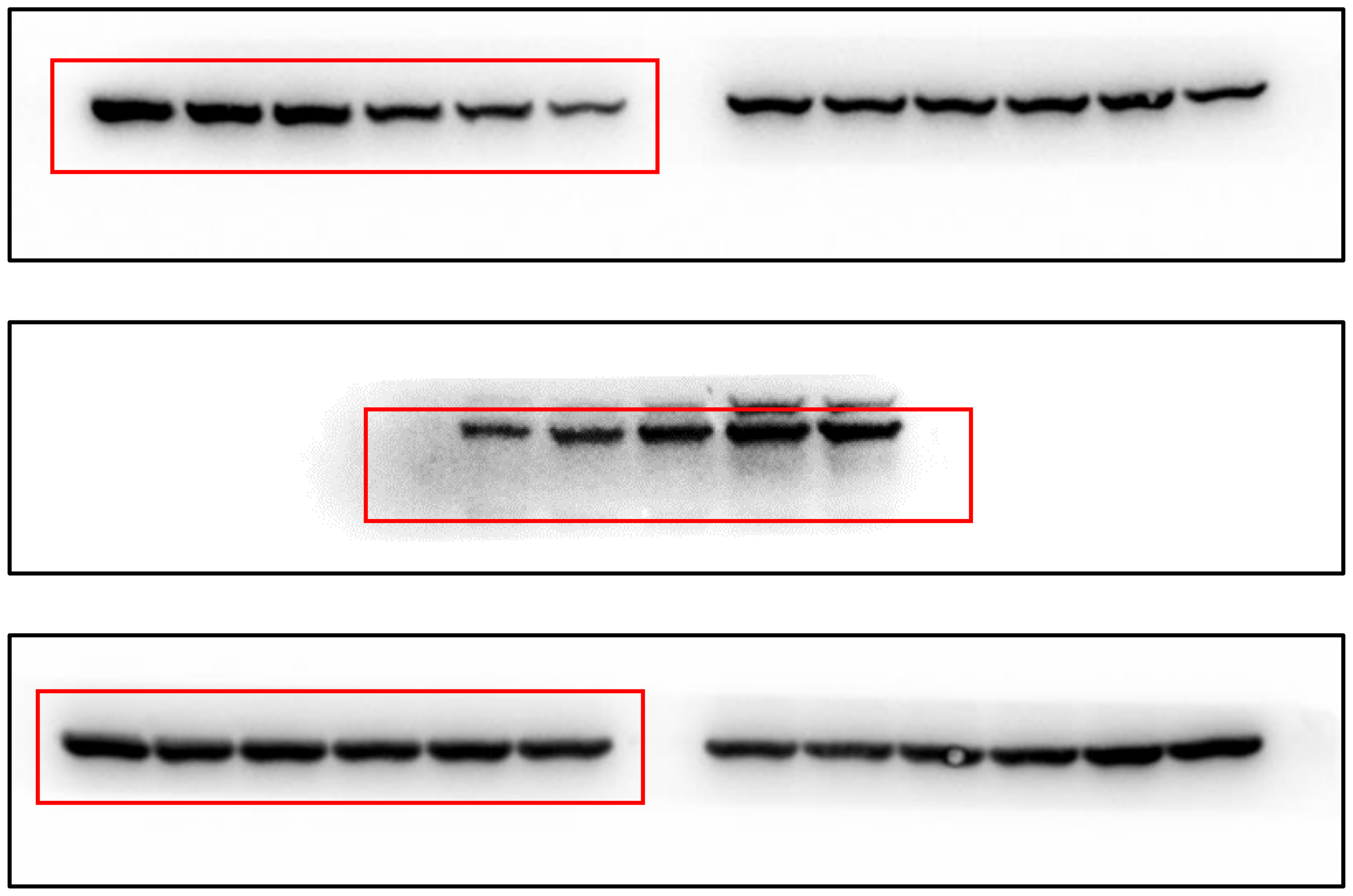

Fig4B

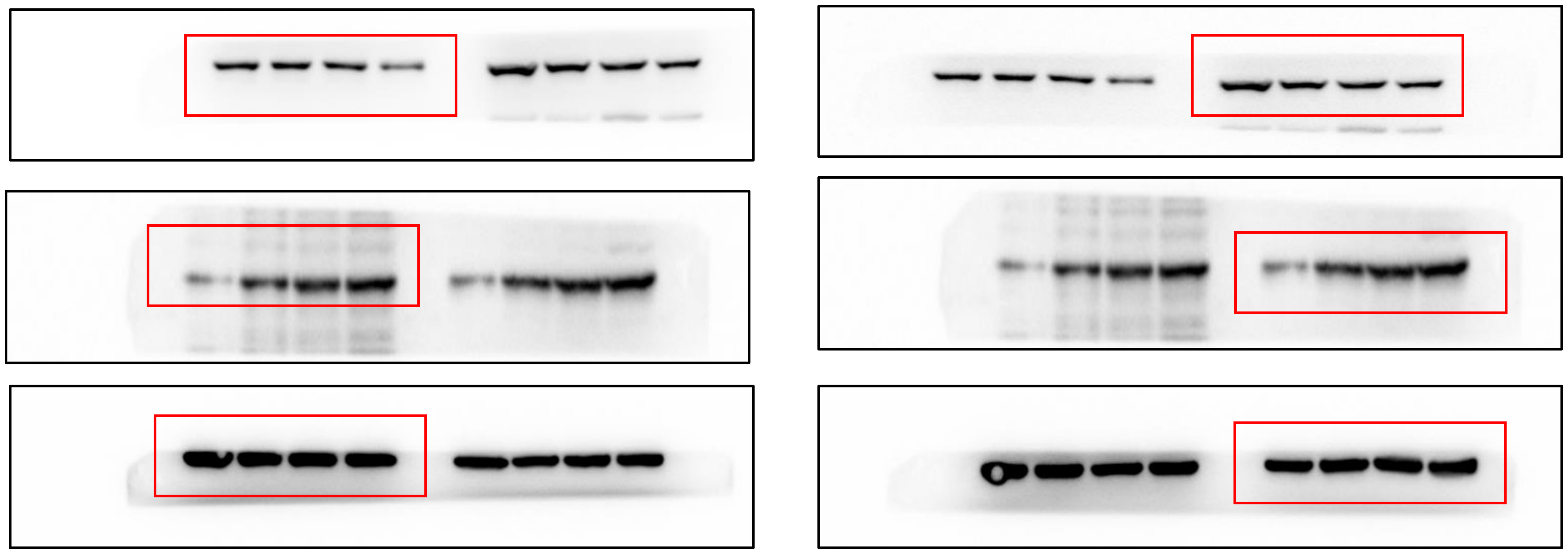

Fig4C

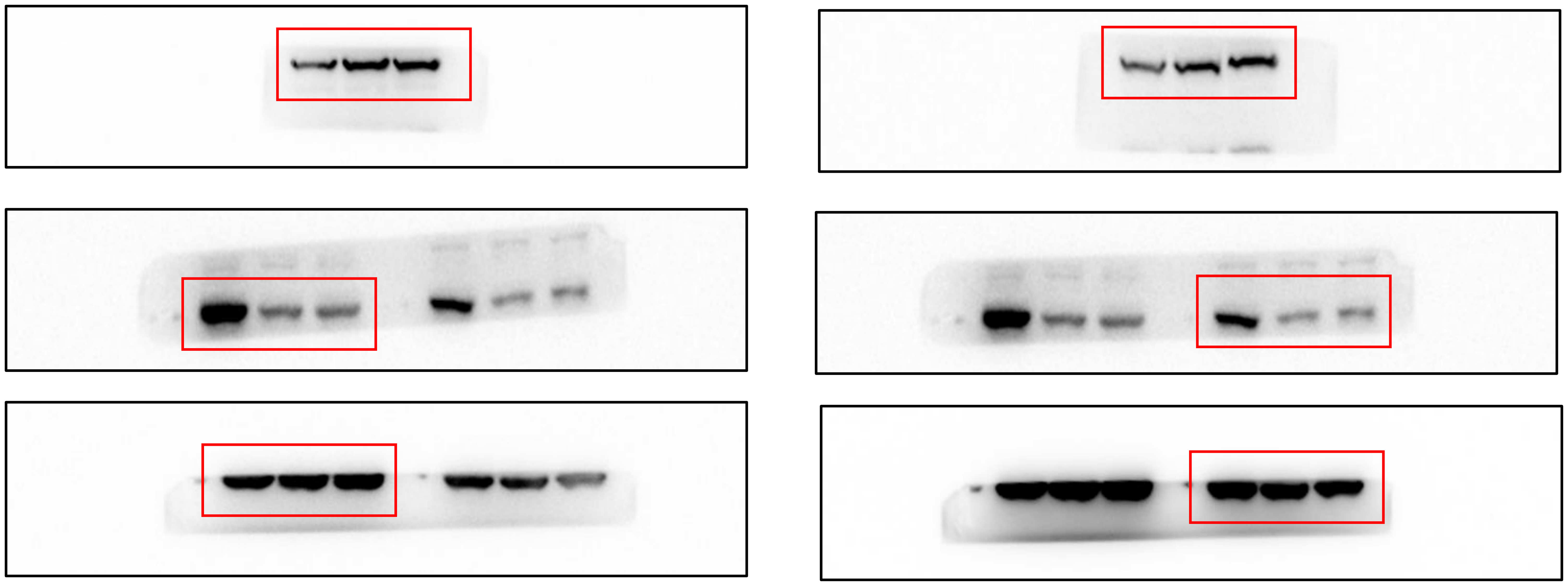

Fig4D

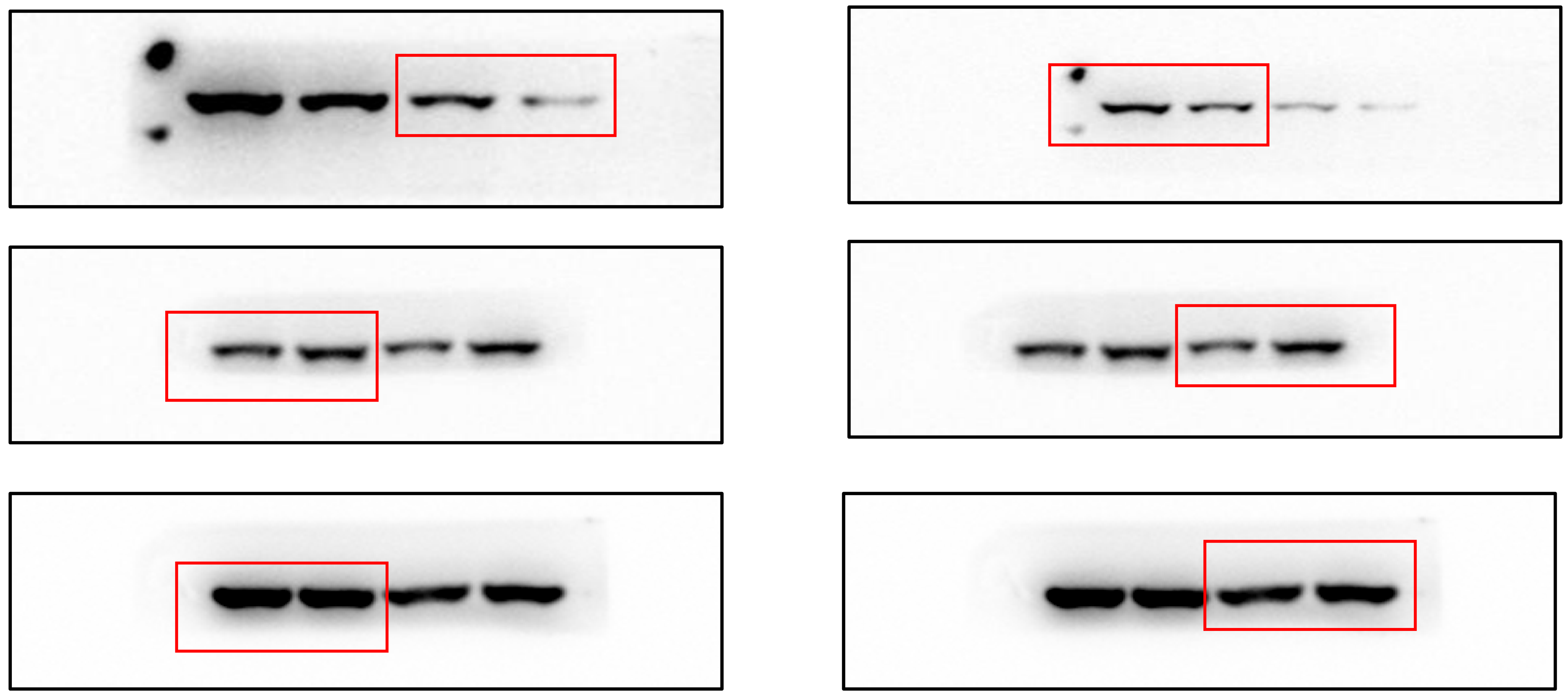

Fig4E

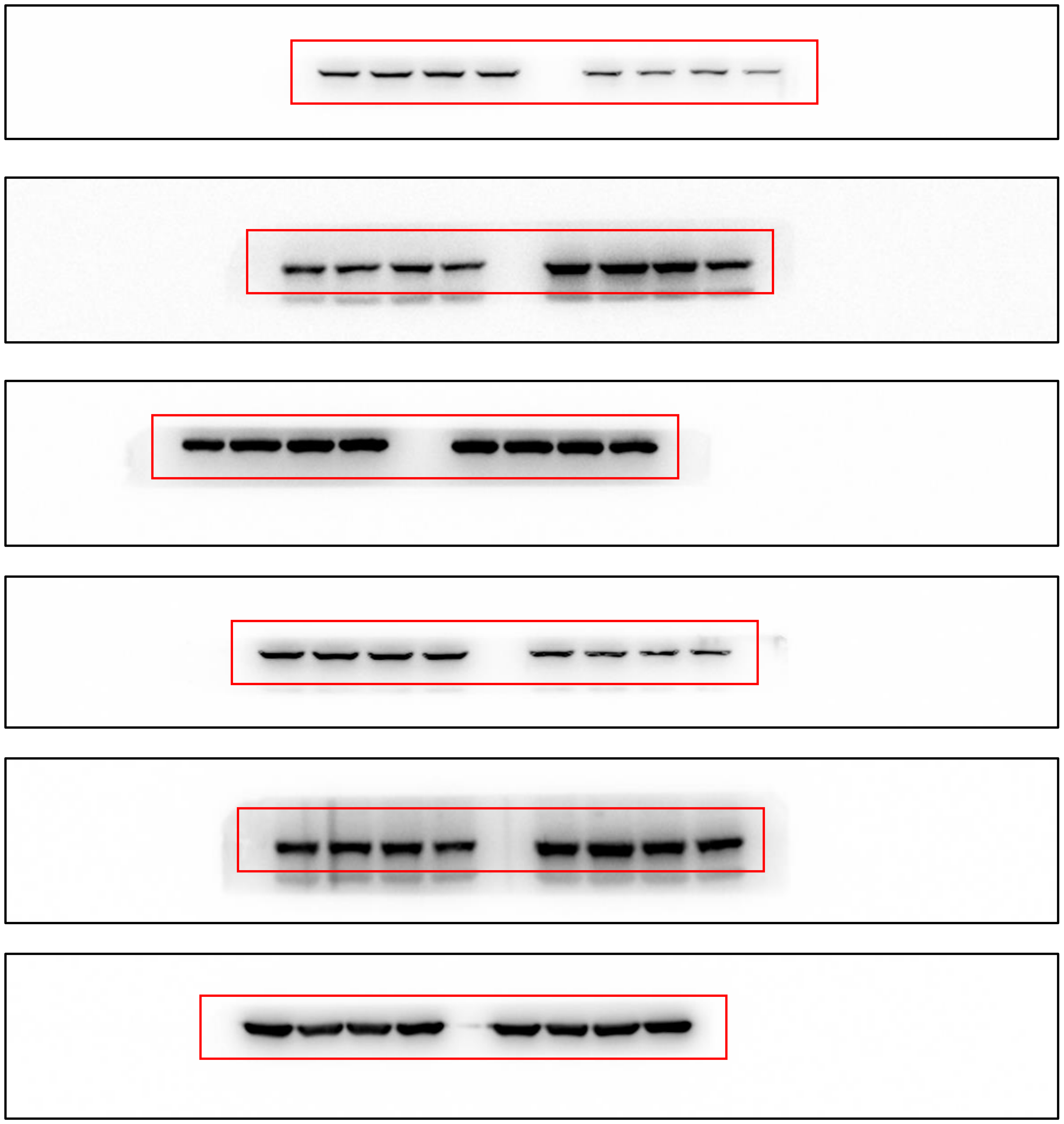

Fig4F

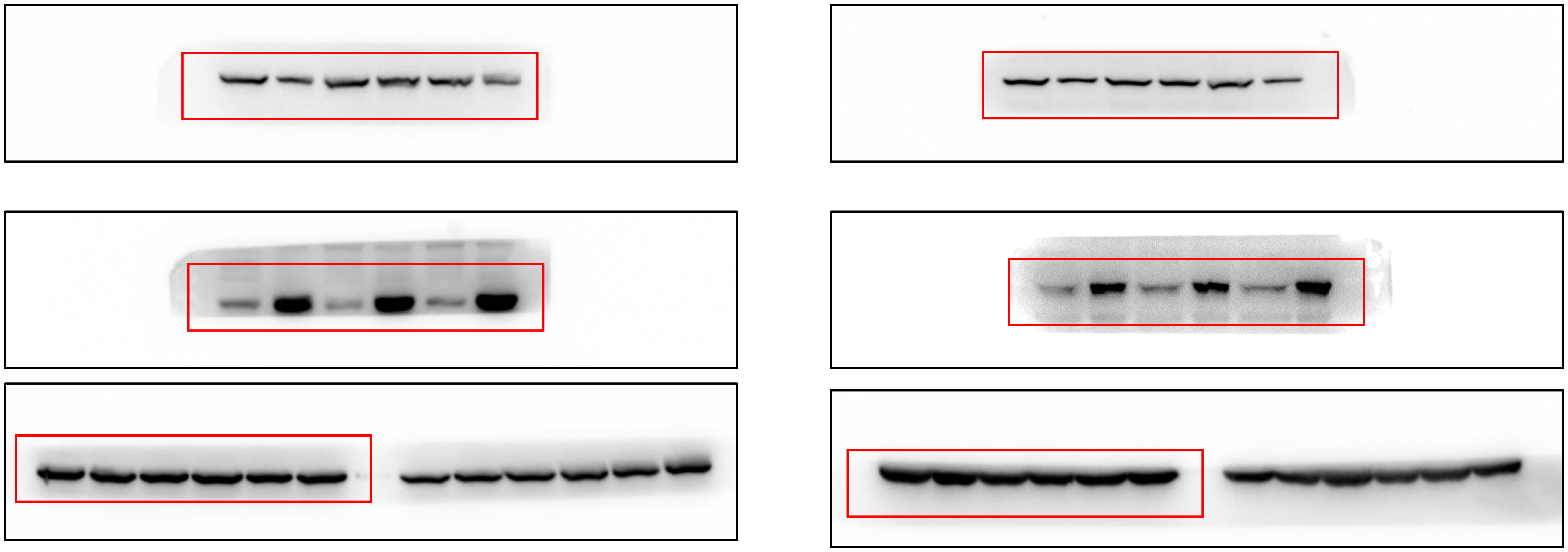

Fig4G

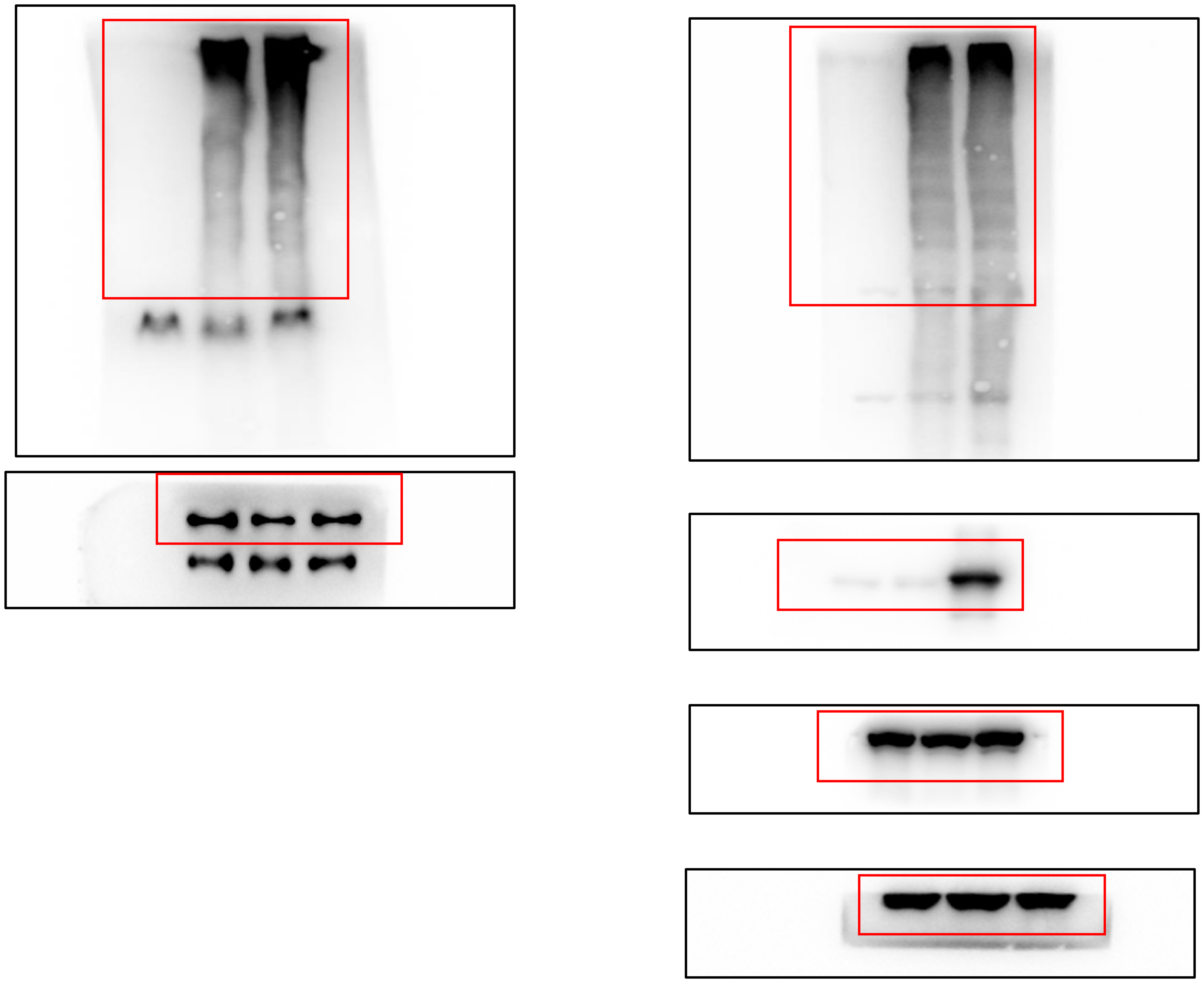

Fig4H

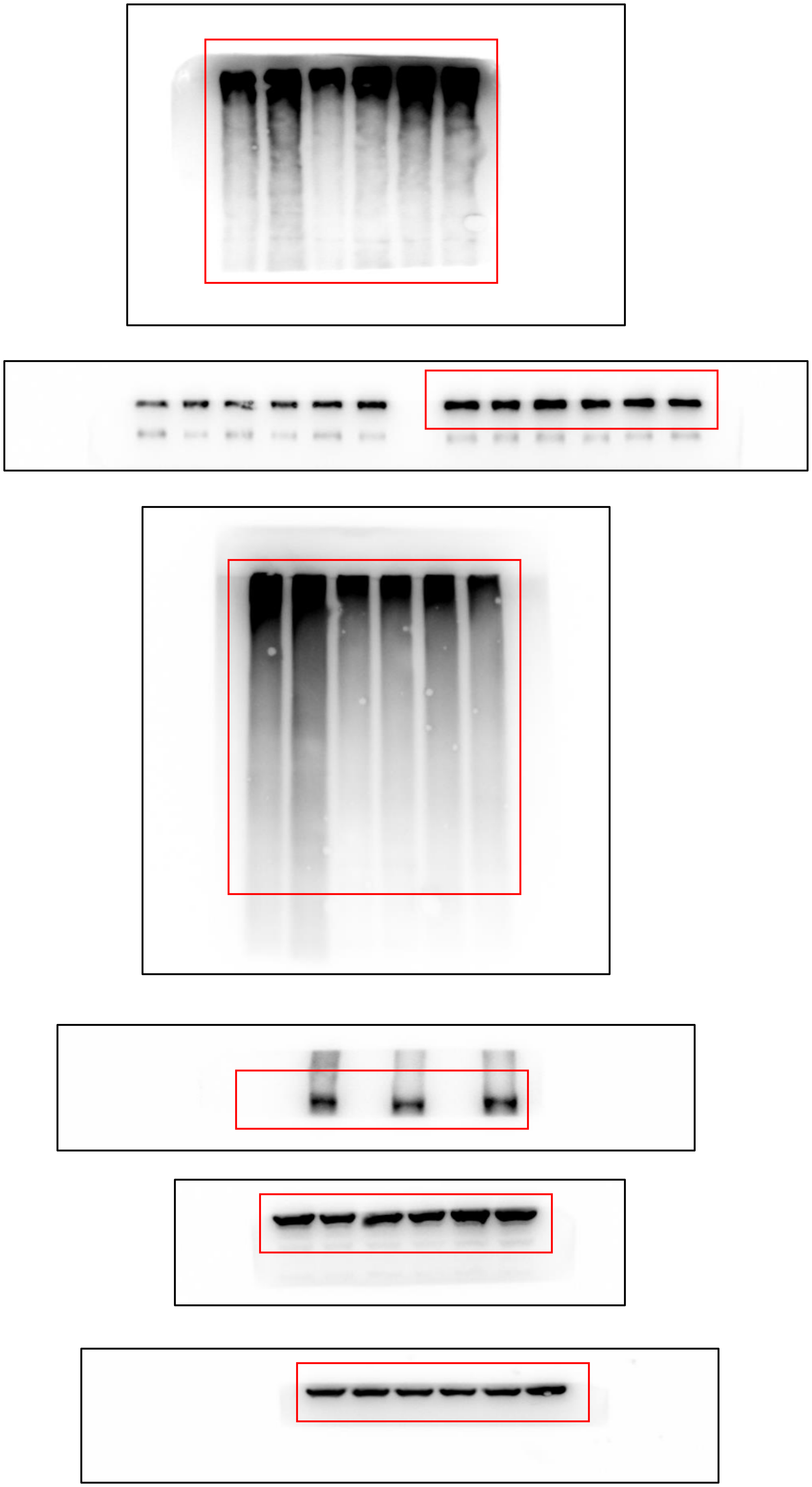

Fig7C

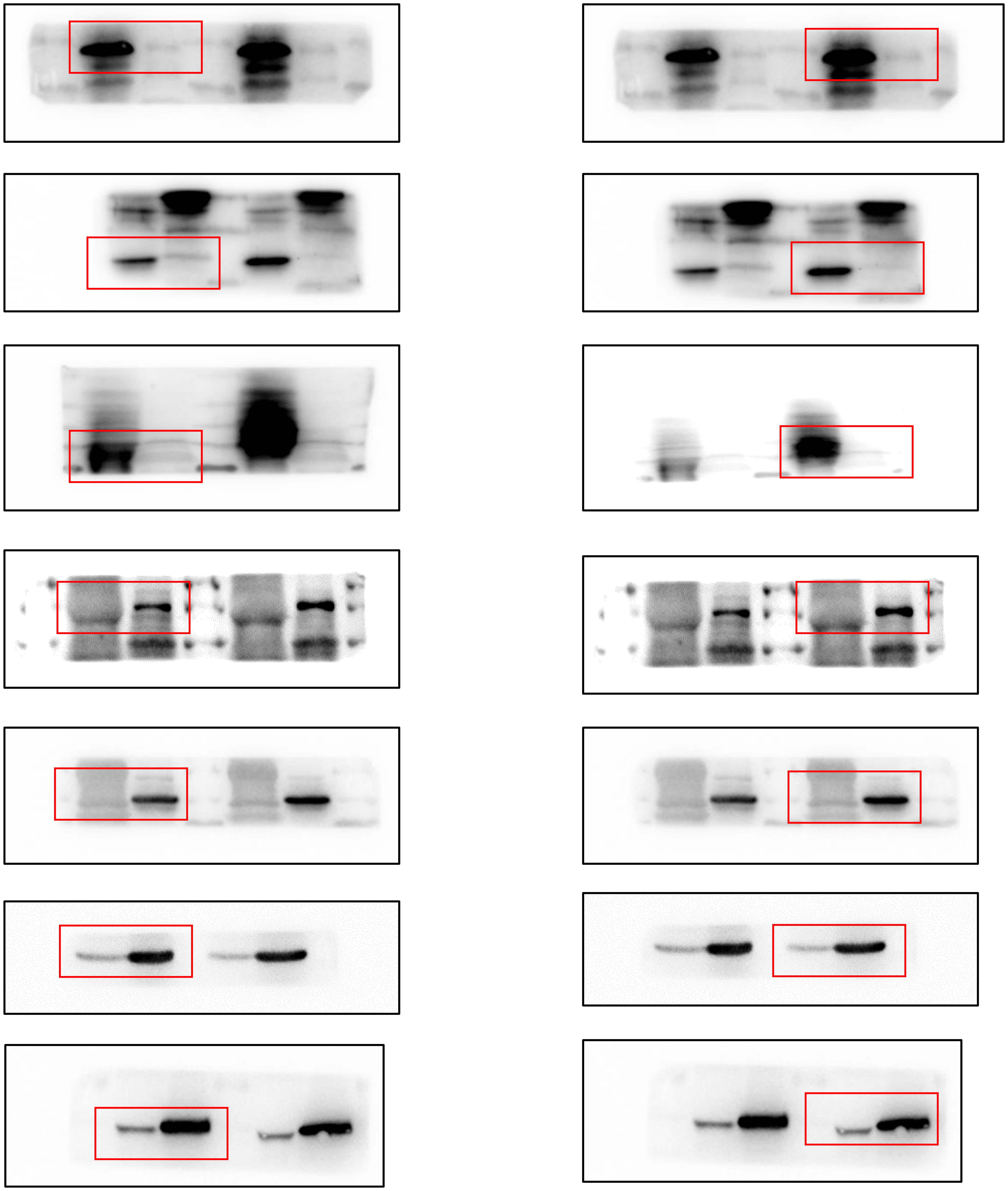

Fig7l

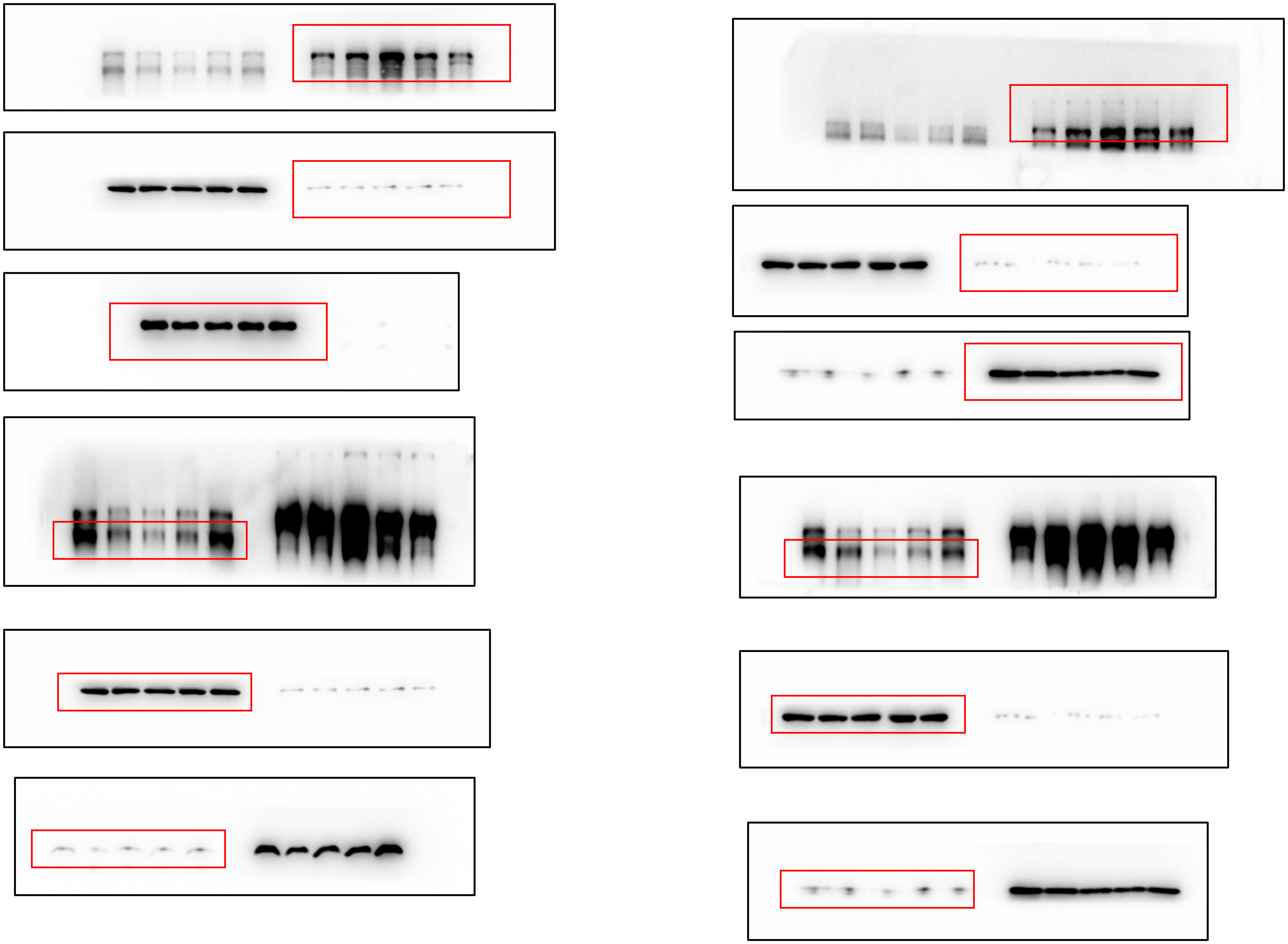

FigS2A

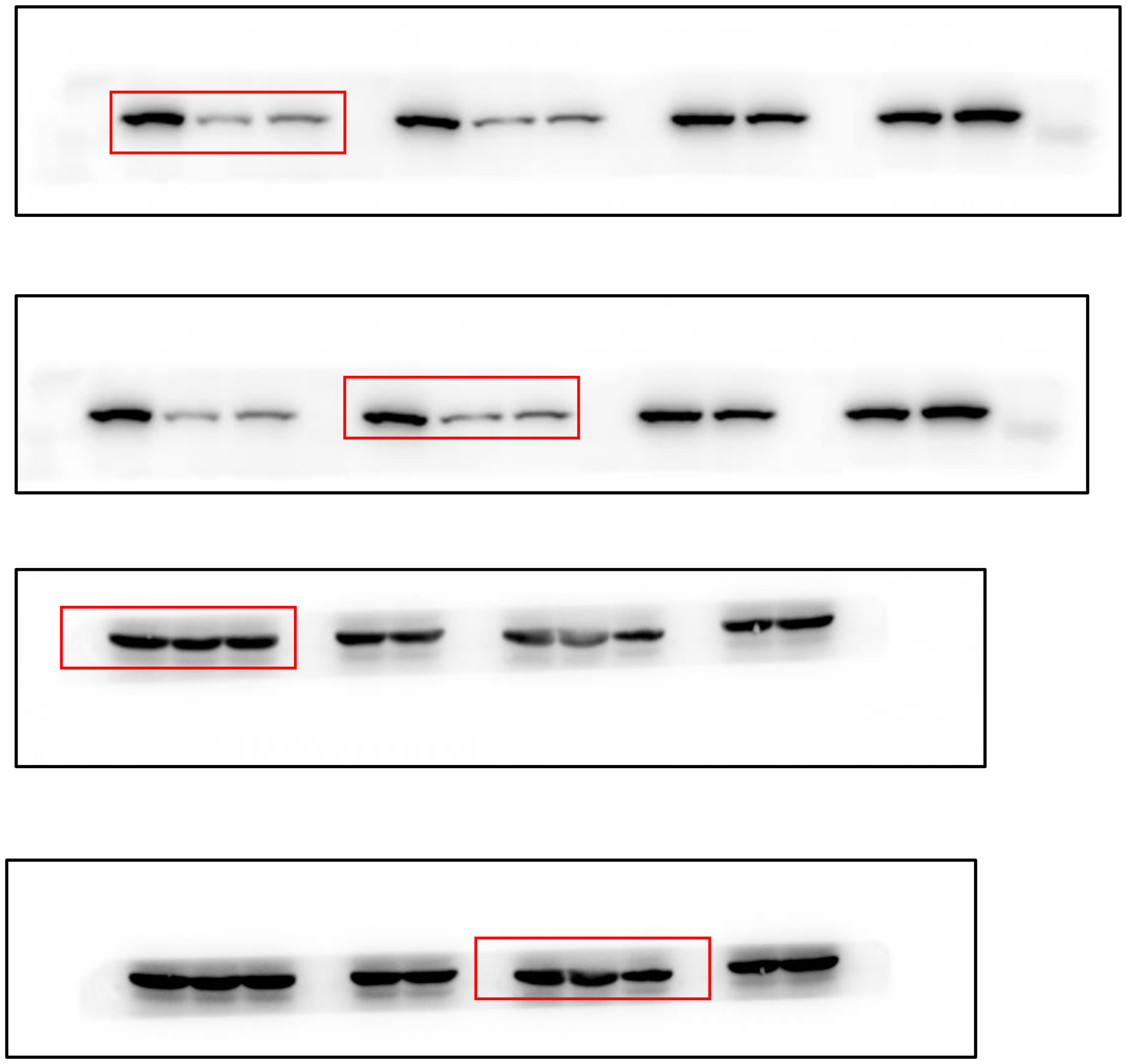

FigS2B

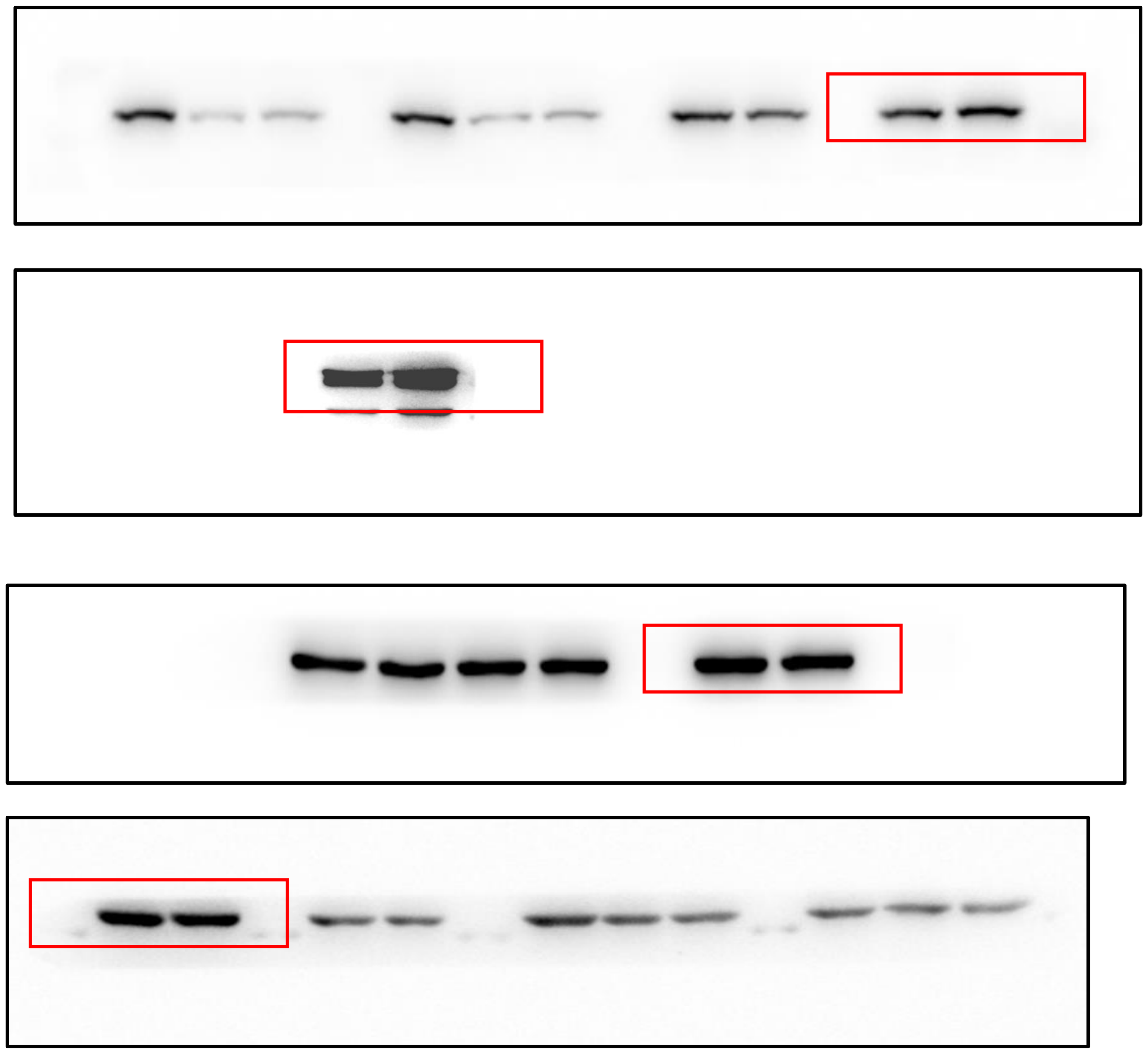

FigS2G

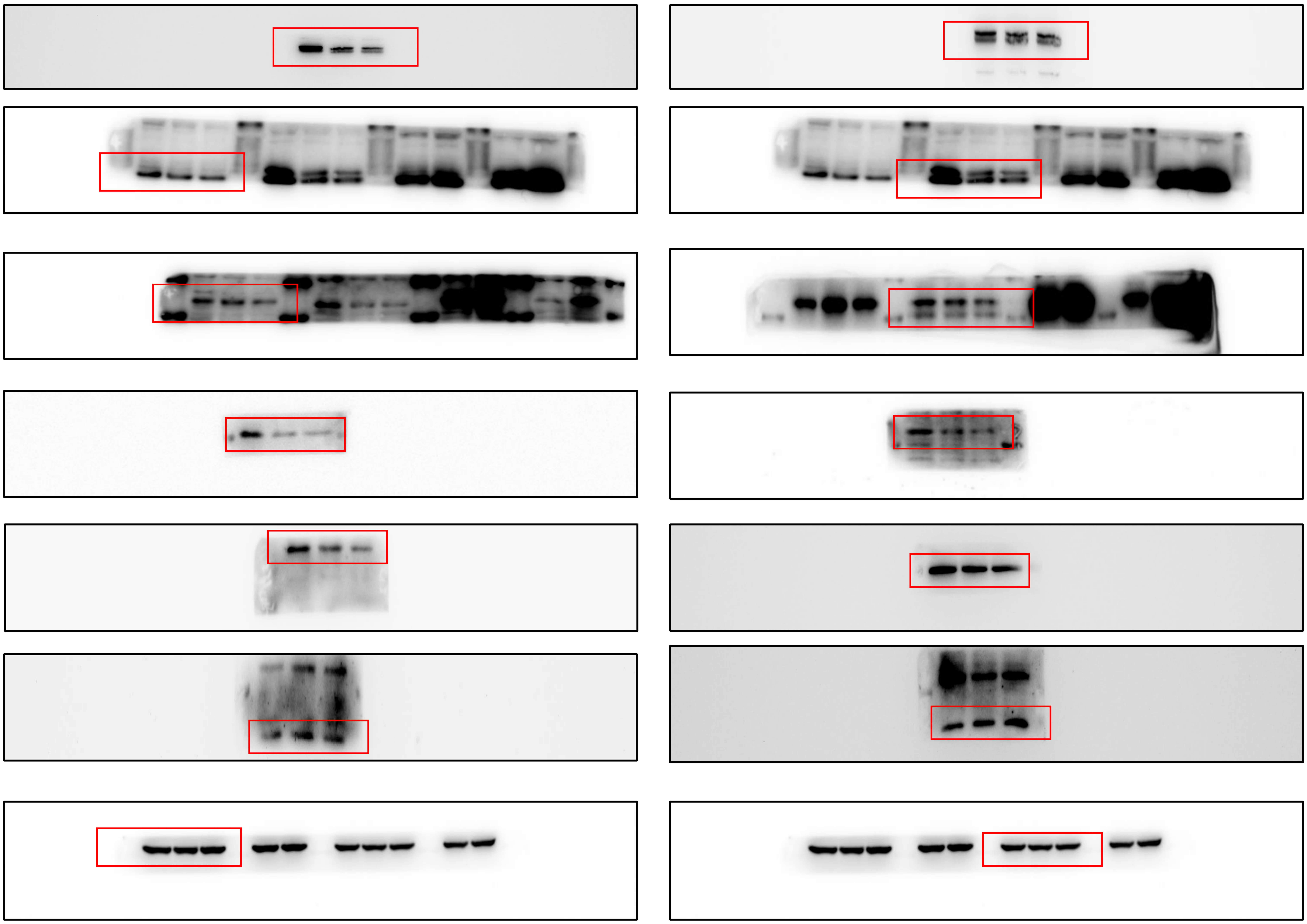

FigS2H

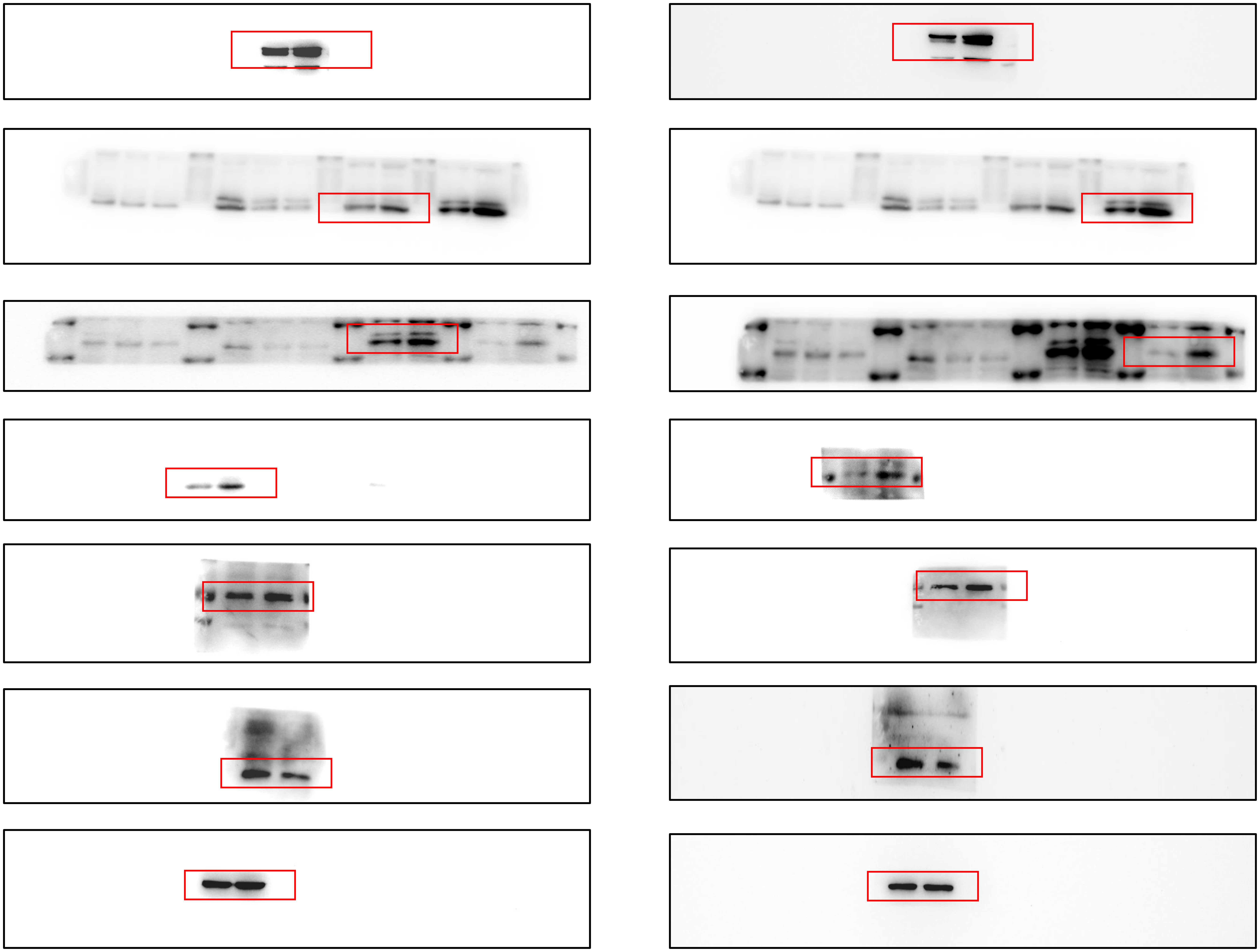

FigS2I

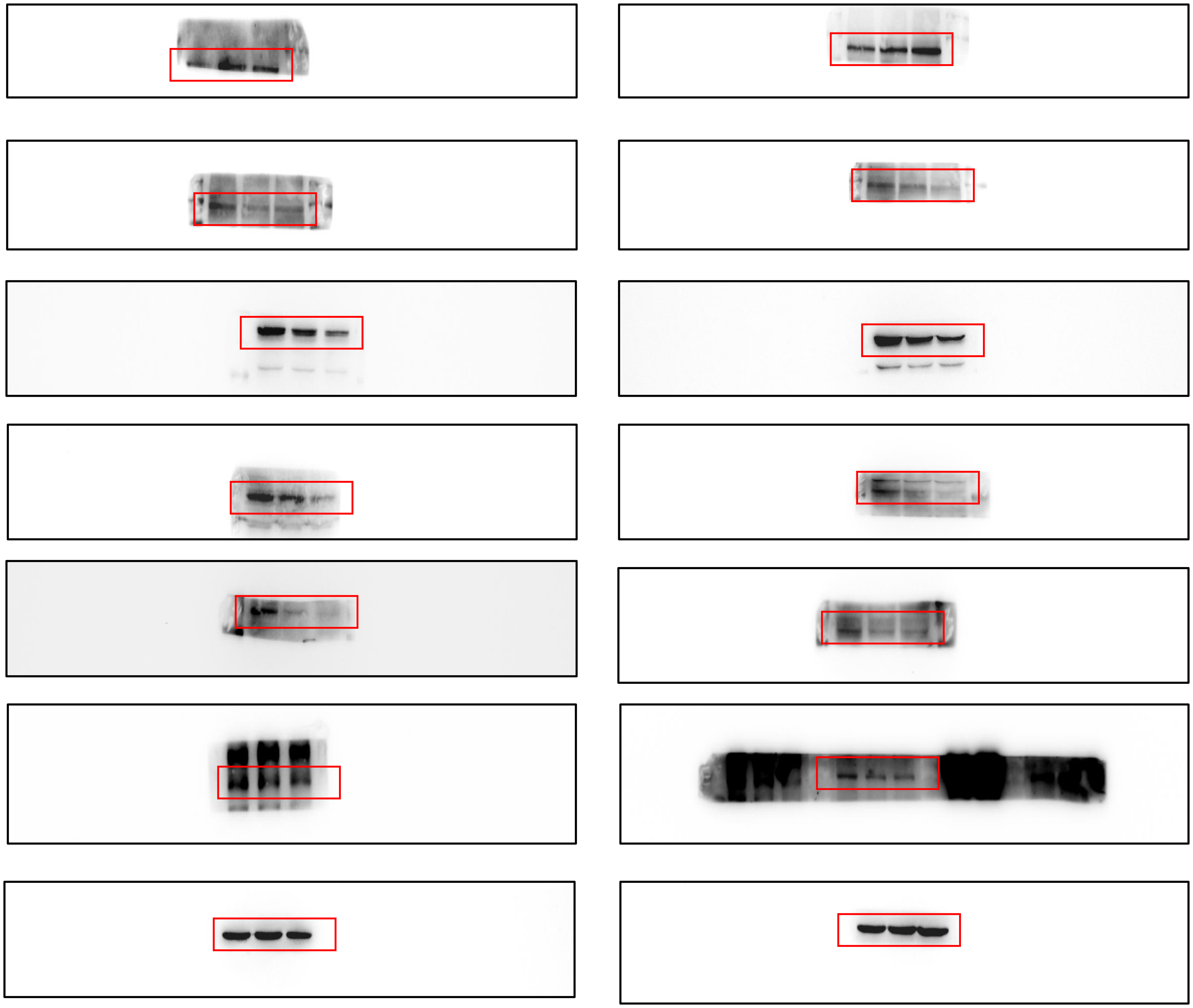

FigS2J

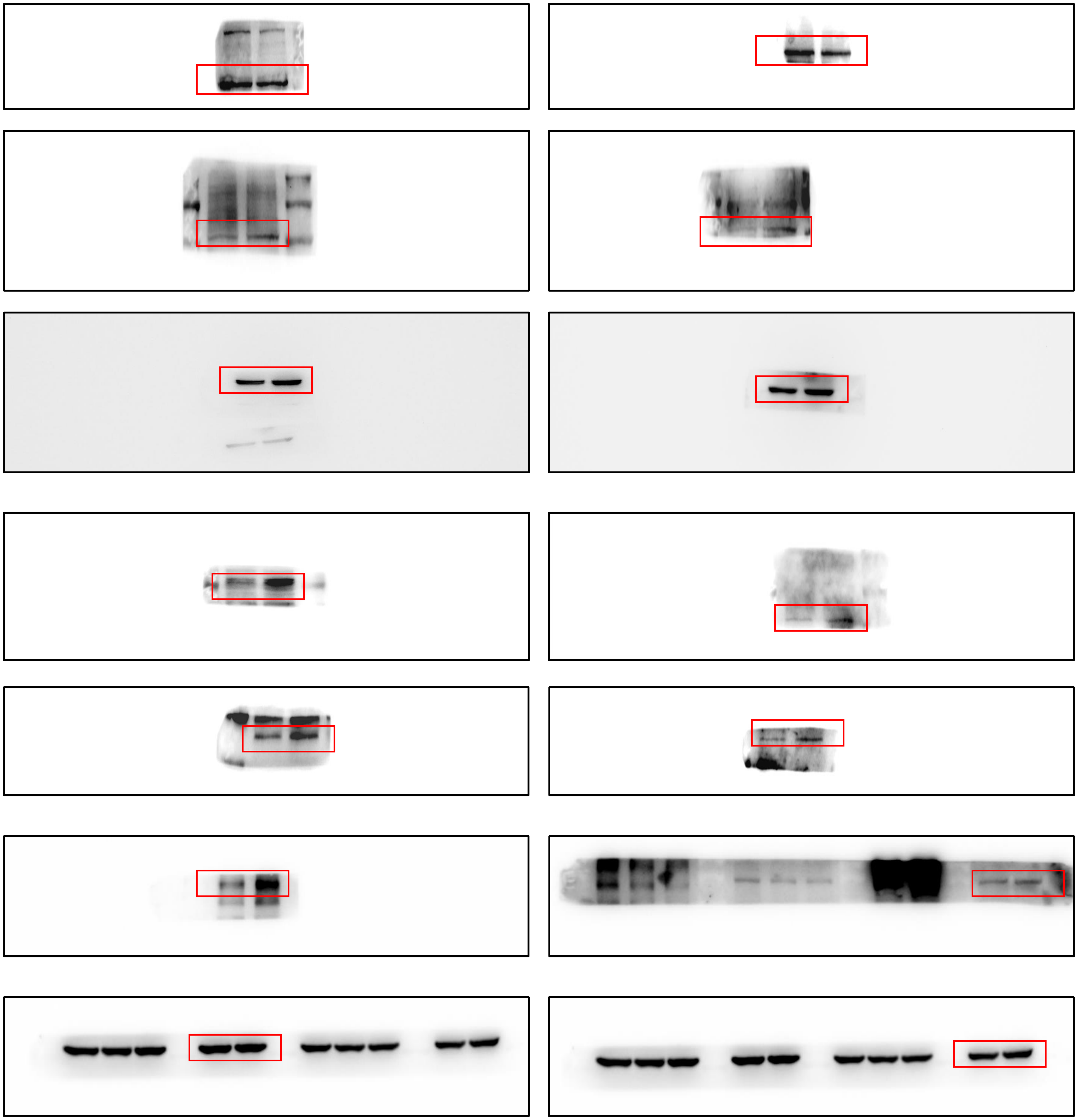

FigS3B

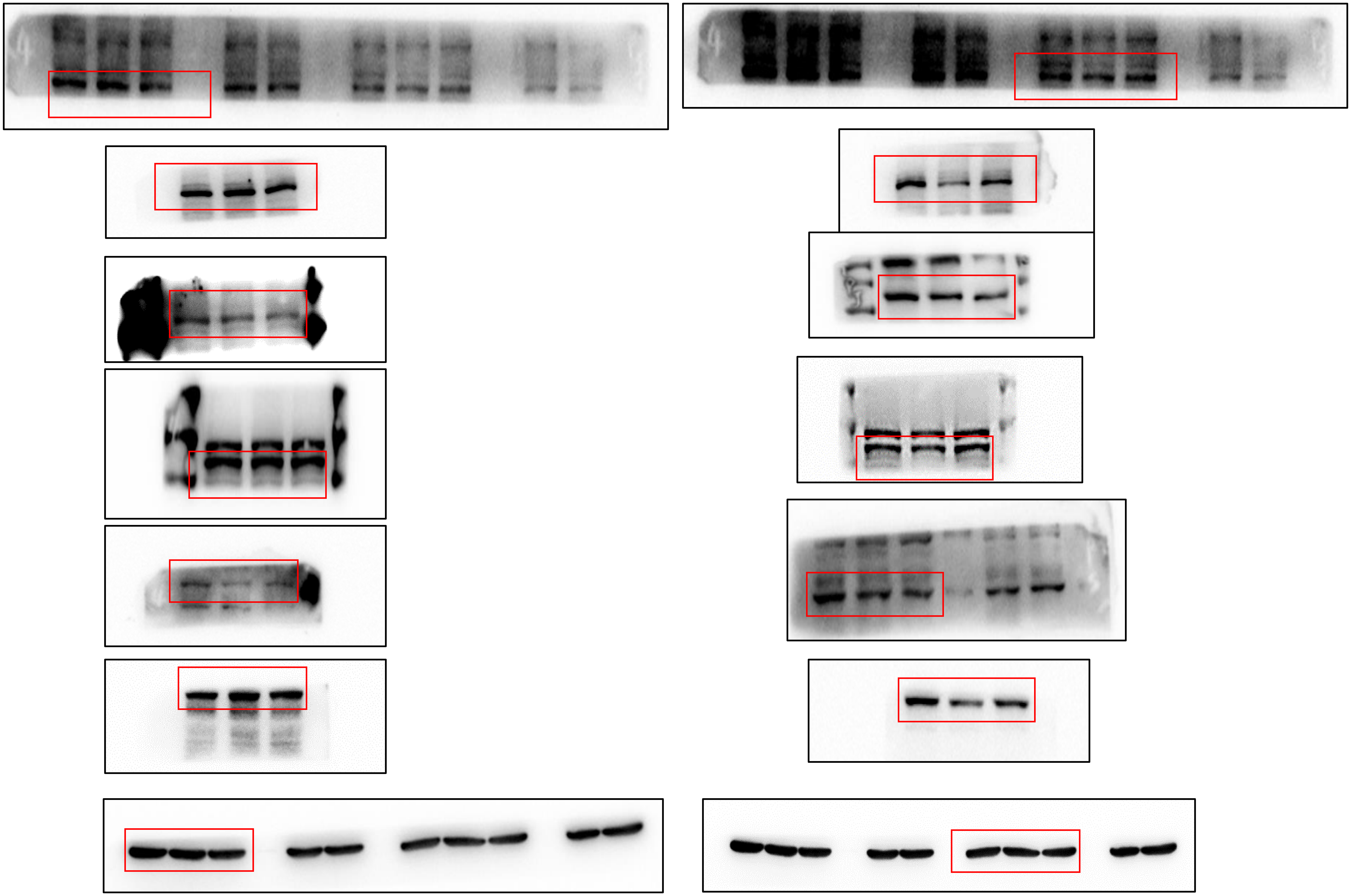

FigS3B

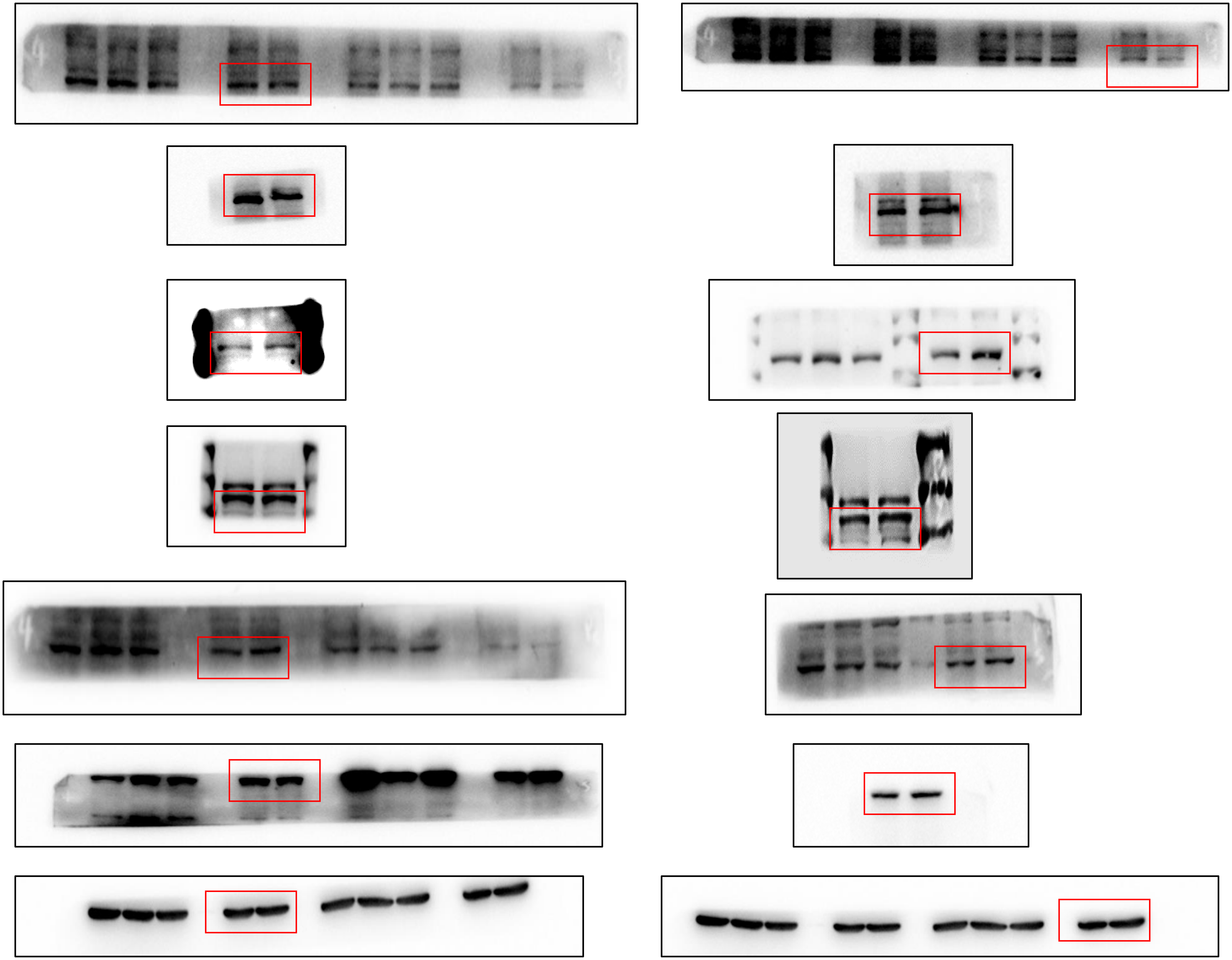

FigS3 H

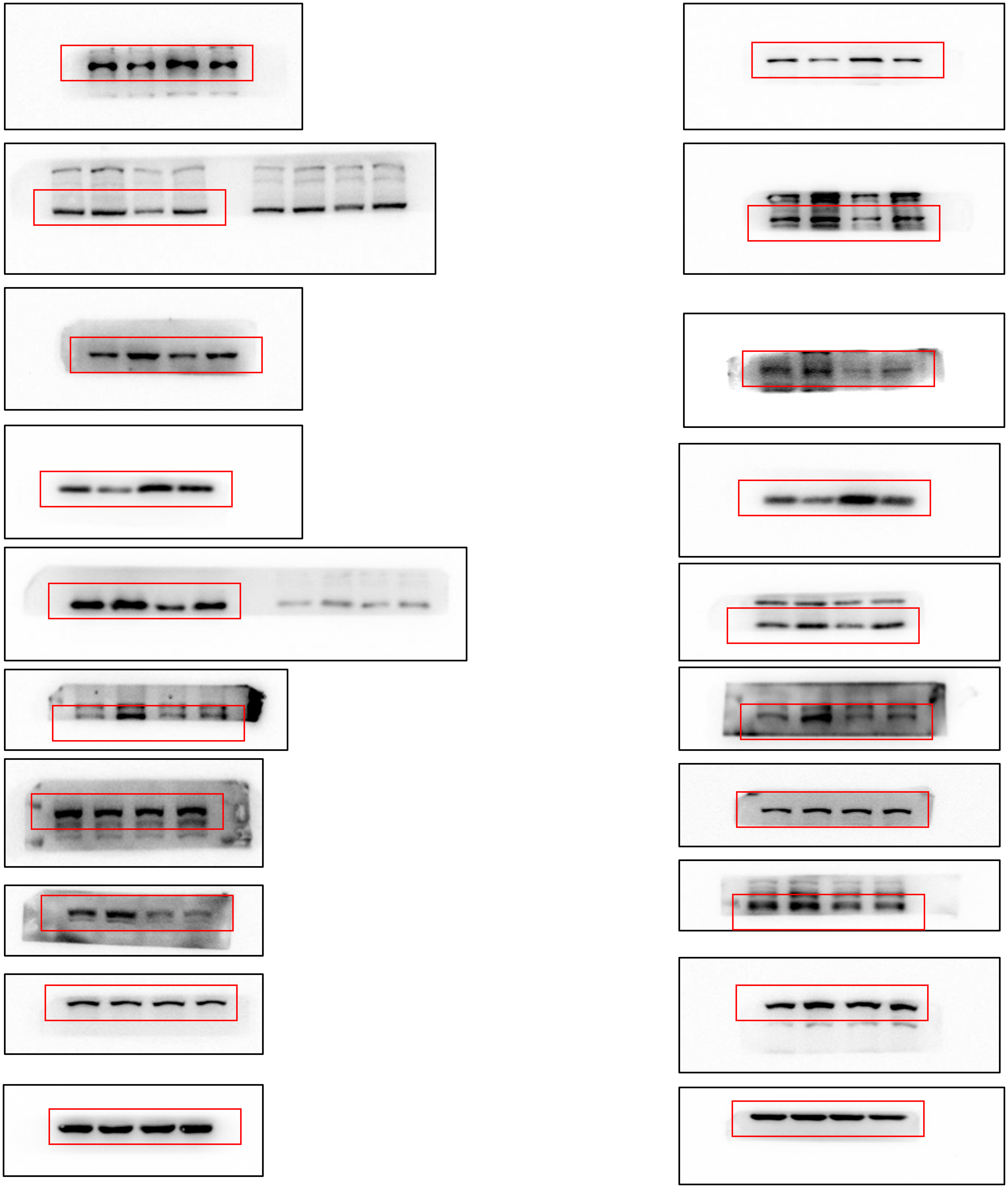

FigS4 A

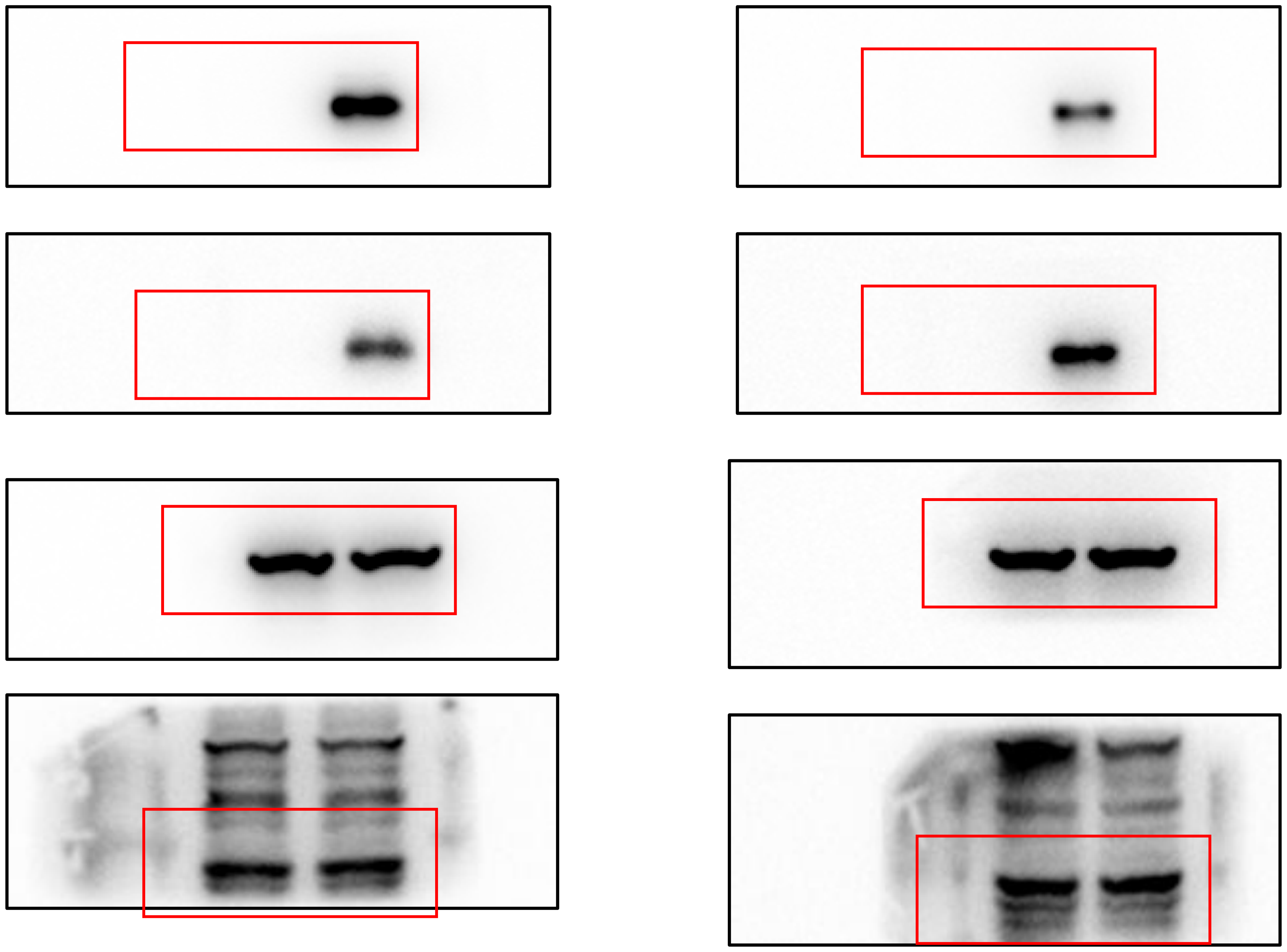

FigS4 C

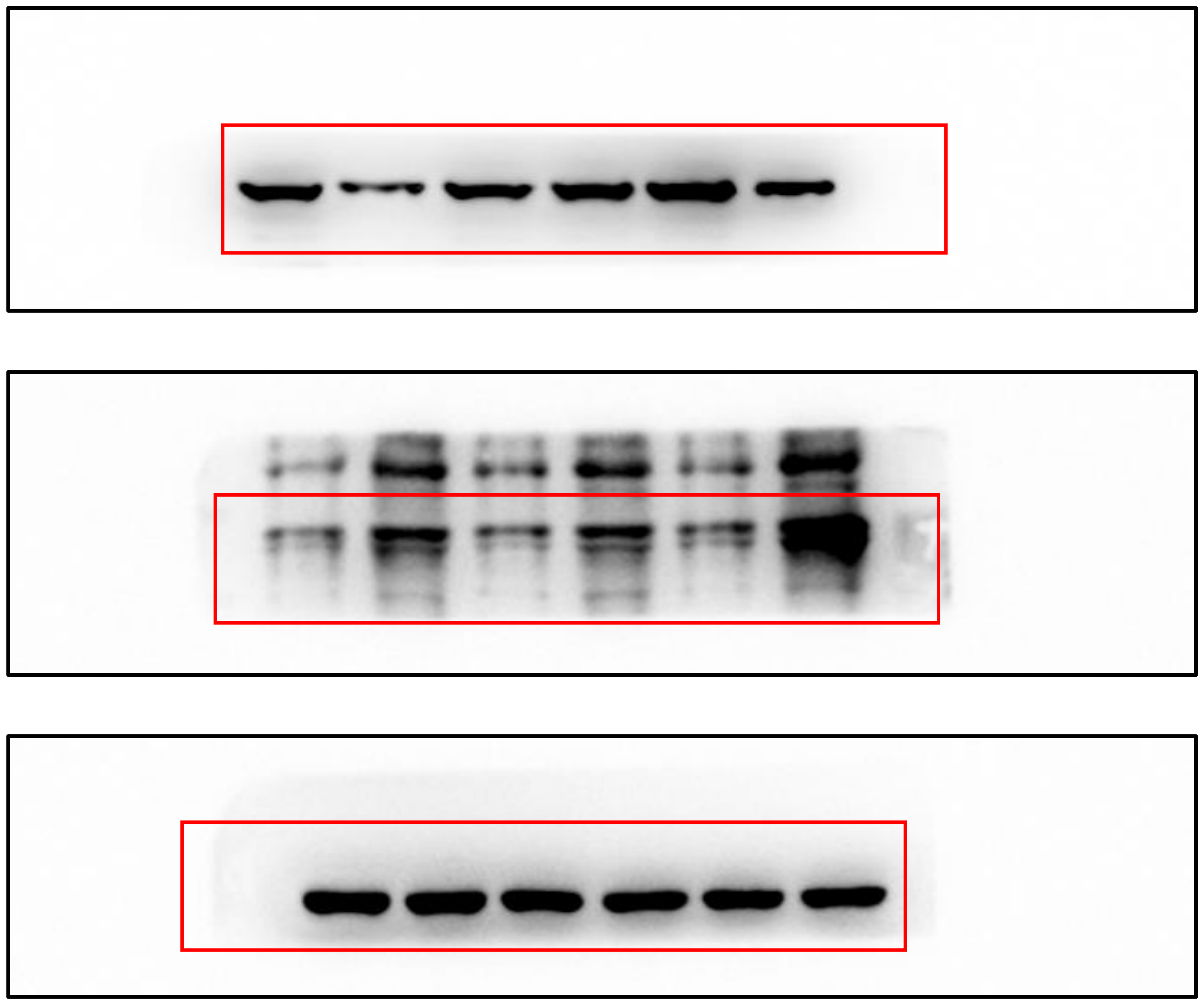

FigS4 B

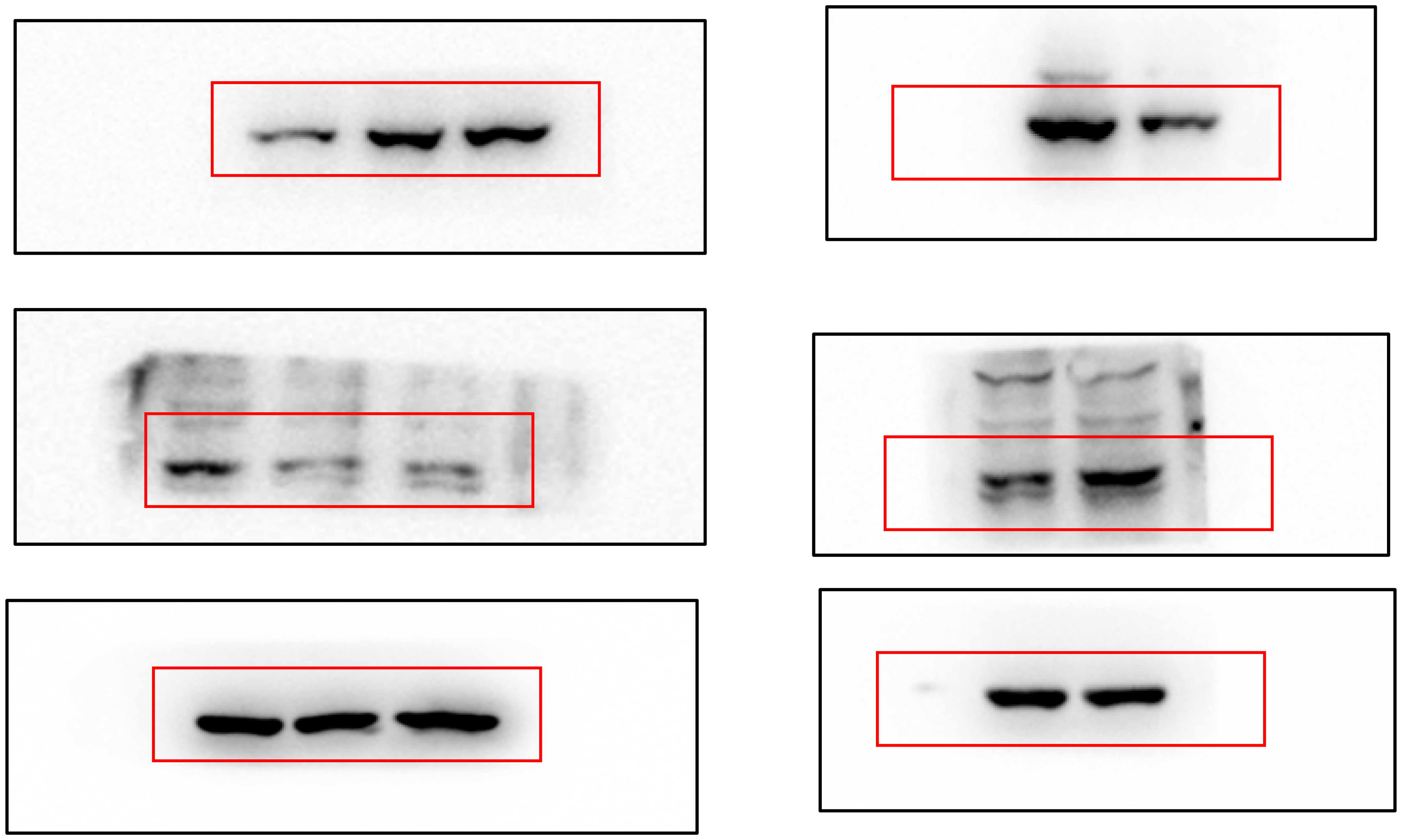

FigS5F

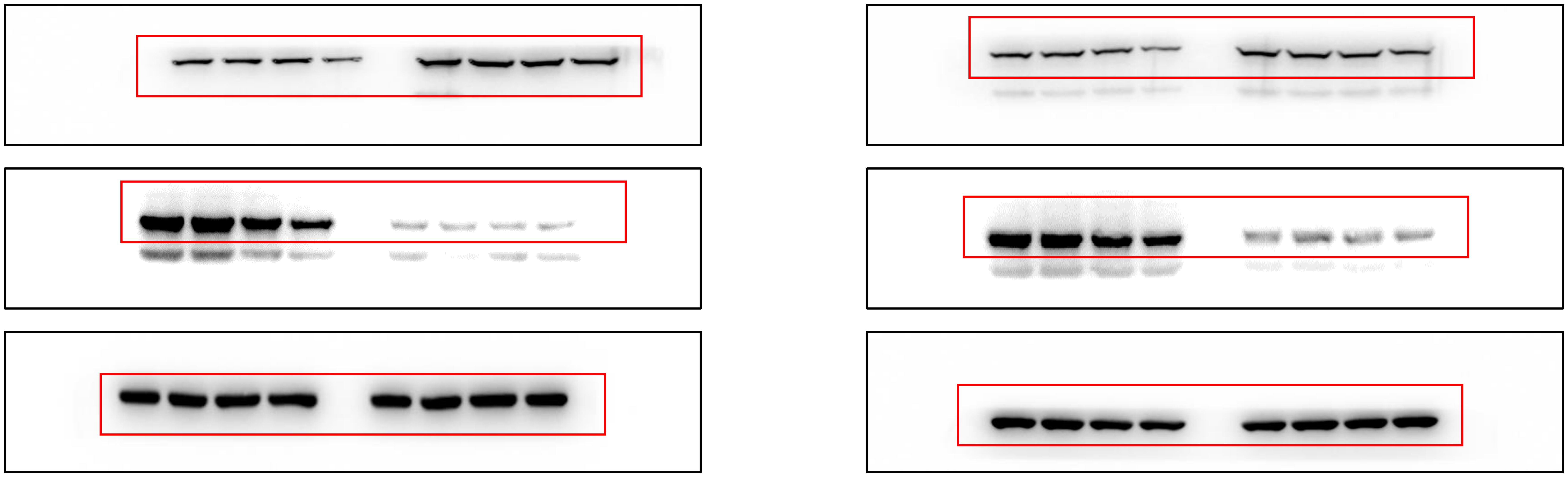

FigS5G

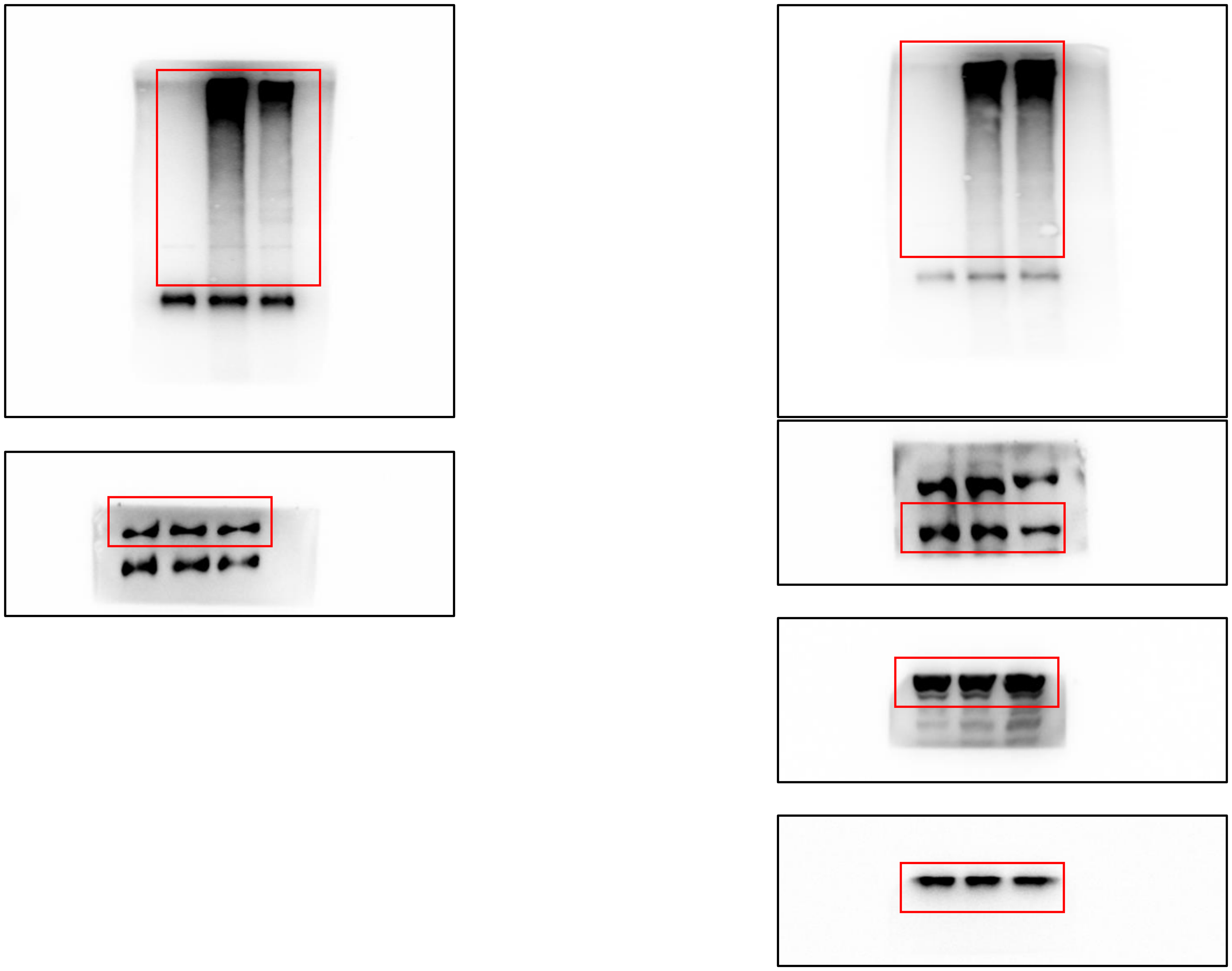

FigS5H

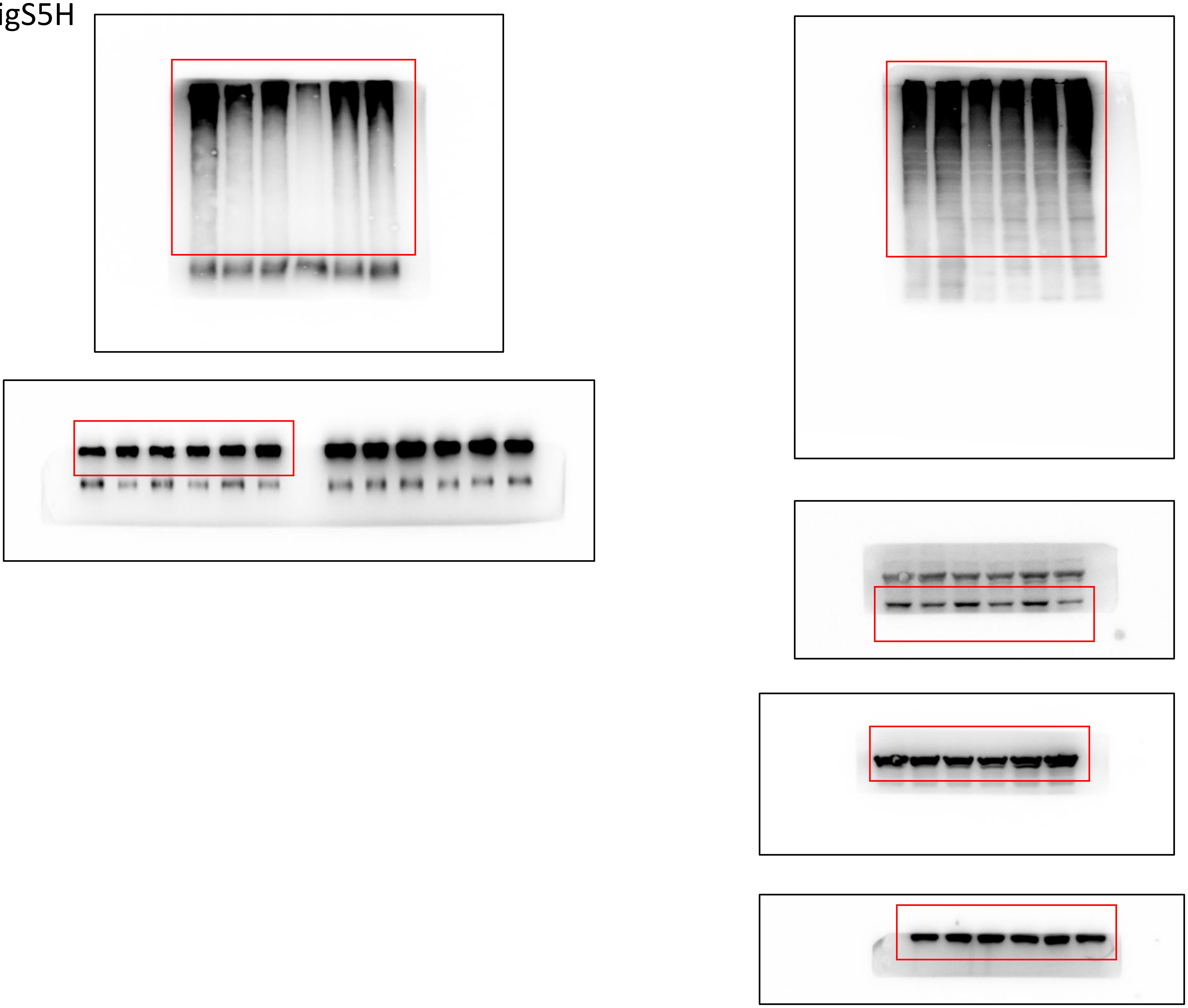

FigS8 F

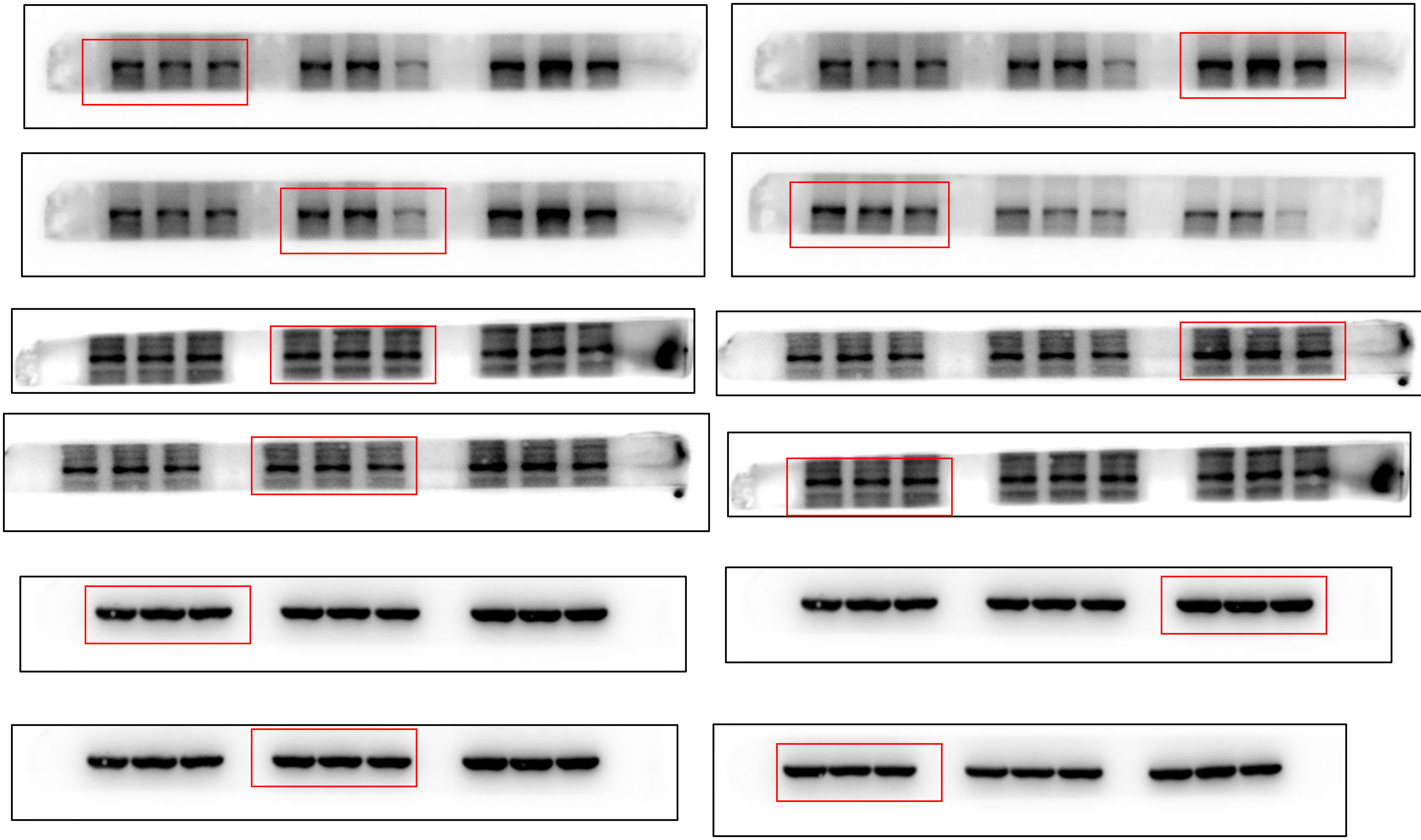

FigS9 A

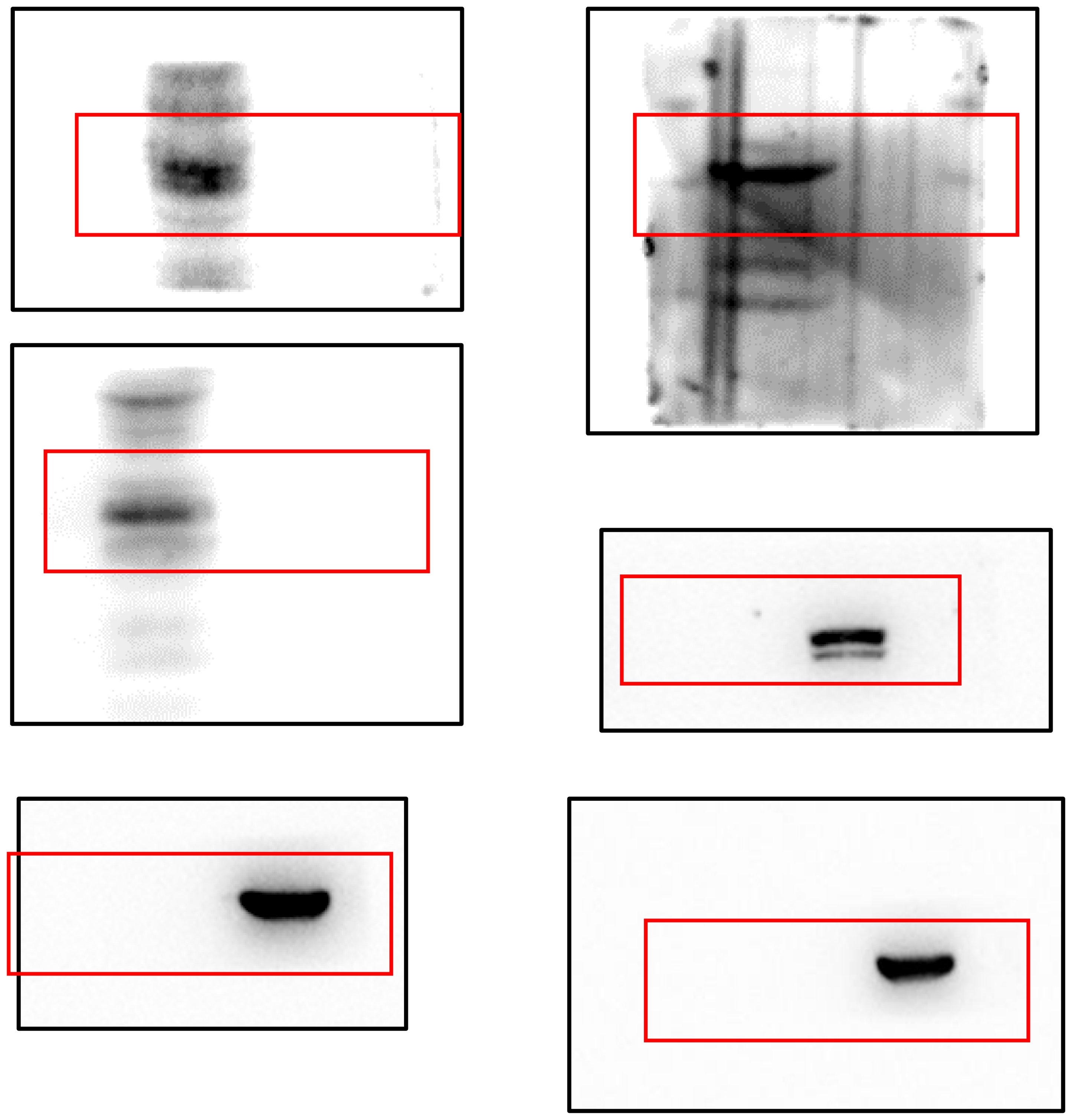

FigS9 B

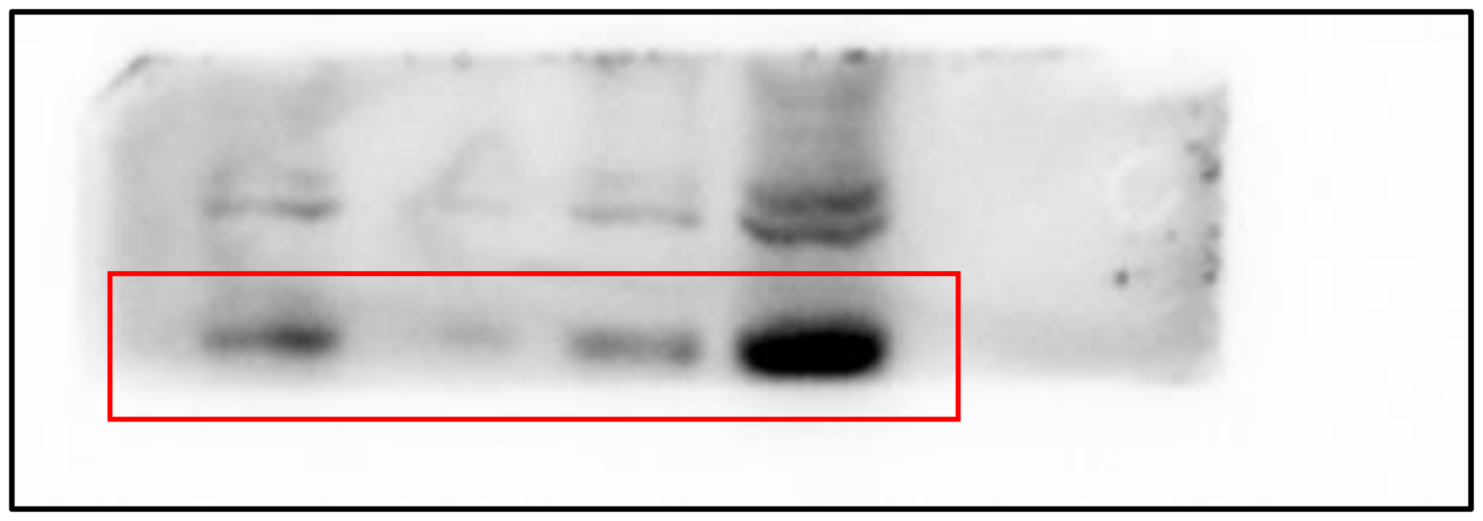

FigS9 D

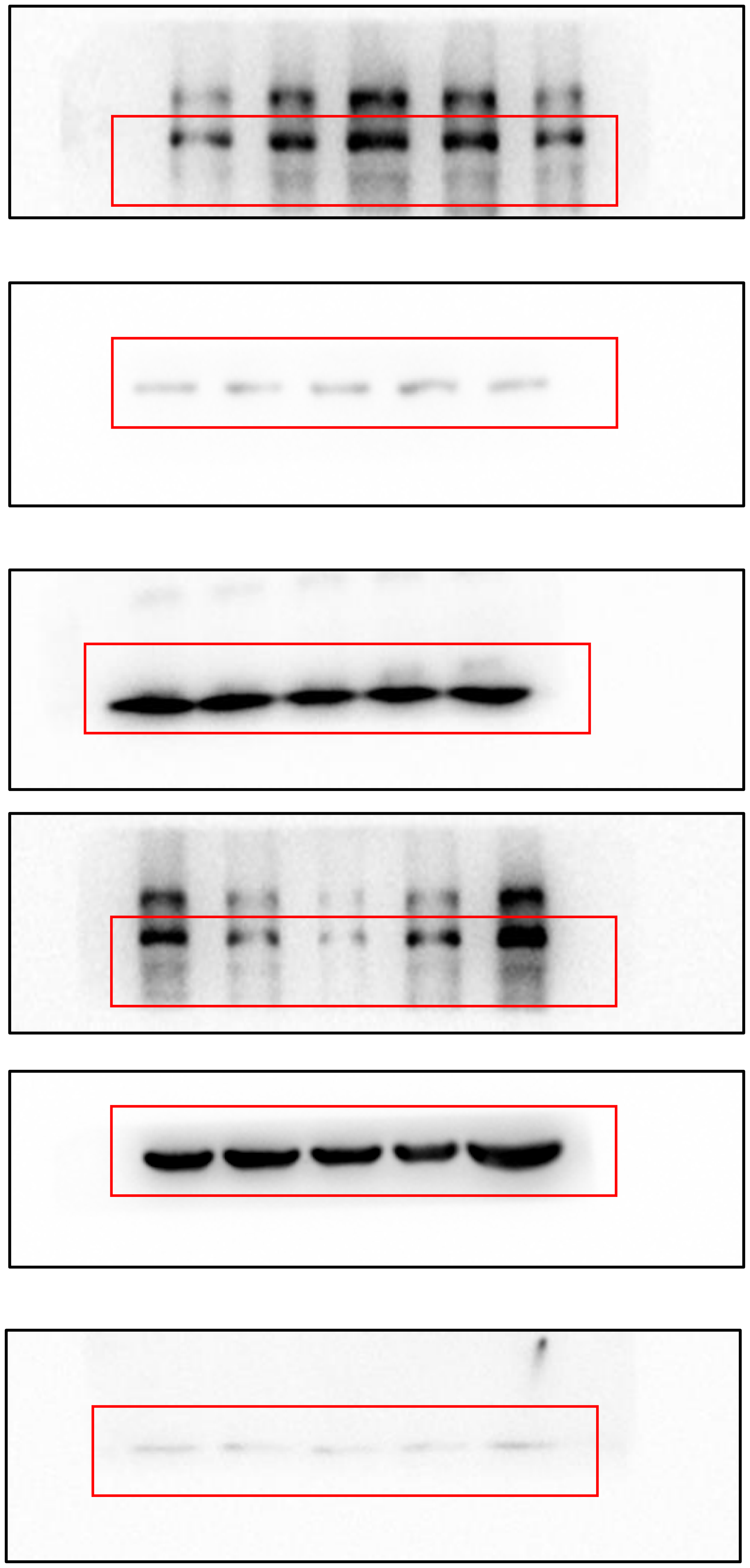

Supplement: Supplementary file 2 — supplementary WB [file 41419_2024_6944_MOESM2_ESM.pdf]
